# Caloric restriction causes a distinct reorganization of the lipidome in quiescent and non-quiescent cells of budding yeast

**DOI:** 10.18632/oncotarget.28133

**Published:** 2021-11-23

**Authors:** Karamat Mohammad, Emmanuel Orfanos, Vladimir I. Titorenko

**Affiliations:** ^1^Department of Biology, Concordia University, Montreal, Quebec H4B 1R6, Canada

**Keywords:** cellular aging, cellular quiescence, caloric restriction, geroprotectors, lipids

## Abstract

After budding yeast cells cultured in a nutrient-rich liquid medium with 0.2% glucose (under caloric restriction conditions) or 2% glucose (under non-caloric restriction conditions), ferment glucose to ethanol and then consume ethanol, they enter the stationary phase. The process of their chronological aging begins. At that point, the yeast culture starts to accumulate quiescent and non-quiescent cells. Here, we purified the high- and low-density populations of quiescent and non-quiescent cells from the yeast cultures limited in calorie supply or not. We then employed mass spectrometry-based quantitative lipidomics to assess the aging-associated changes in high- and low-density cells’ lipidomes. We found that caloric restriction, a geroprotective dietary intervention, alters the concentrations of many lipid classes through most of the chronological lifespan of the high- and low-density populations of quiescent and non-quiescent cells. Specifically, caloric restriction decreased triacylglycerol, increased free fatty acid, elevated phospholipid and amplified cardiolipin concentrations. Based on these findings, we propose a hypothetical model for a caloric restriction-dependent reorganization of lipid metabolism in budding yeast’s quiescent and non-quiescent cells. We also discovered that caloric restriction creates lipidomic patterns of these cells that differ from those established by two other robust geroprotectors, namely the *tor1Δ* mutation and lithocholic acid.

## INTRODUCTION

Culturing budding yeast *Saccharomyces cerevisiae* aerobically in a nutrient-rich liquid medium with 2% glucose as a single carbon source provides yeast enough calories to proliferate and survive [1]. The term “non-caloric restriction (non-CR)” was coined to describe these conditions of cell culturing [2]. At some point of culturing under non-CR conditions, *S. cerevisiae* cells consume exogenous glucose and slow their growth [2]. These cells enter a diauxic shift period, during which they produce ethanol and catabolize it aerobically [1]. After consuming ethanol, yeast culture’s growth is further decelerated [2]. The culture enters a post-diauxic shift marked by active mitochondrial respiration [2]. At that point, the cell cycle of yeast in the culture is arrested in the late G_1_ phase [3]. This cell cycle arrest occurs at the nutrient-dependent checkpoint called “START A” [3]. The cell cycle arrest at the “START A” checkpoint coincides with the appearance of two cell populations in the yeast culture under non-CR conditions. These populations are quiescent (Q) and non-quiescent (NQ) cells [4]. The physical, morphological, reproductive, biochemical and physiological properties of the Q and NQ cell populations are different [4]. These properties are developed under the control of a nutrient-sensing signaling network that integrates several signaling pathways and protein kinases [5].

After the post-diauxic shift on the non-CR culture is completed, it enters the stationary (ST) culturing phase [6]. At that point, the cultured yeast cells enter a G_0_ state of quiescence, and their chronological aging begins [4, 6]. The length of time during which a yeast cell maintains a G_0_ state of quiescence is traditionally used to measure the pace of chronological aging in *S. cerevisiae* [6, 7]. The maintenance of quiescence is monitored with the help of a clonogenic plating assay. In this assay, a yeast cell from an ST-phase liquid culture that consumed nutrients is plated on the surface of a solid medium rich in nutrients. If the plated cell can form a colony on this solid medium’s surface, this cell is considered viable [6, 7]. The yeast chronological aging assay measures a time-dependent loss in quiescent cells’ viability within the ST-phase cell culture [6, 7]. It is commonly accepted that the chronological aging in yeast mimics the aging of post-mitotic quiescent cells like neurons or myocytes in multicellular eukaryotes [6, 7].

Noteworthy, a multi-step cellular quiescence program operates in and defines properties of the Q and NQ yeast cells under non-CR conditions [4, 8–10]. The program begins when yeast cells enter the diauxic period of growth and continues during the ST phase of culturing [4, 10]. This cellular quiescence program is an essential contributor to the chronological aging of budding yeast not limited in calorie supply [4, 10]. The major properties of the cellular quiescence program operating in yeast under non-CR conditions have been described as follows [4, 5, 11]: 1) the program is initiated in response to nutrient deprivation and the onset of chronological aging, 2) the program integrates a series of cellular events; these events follow each other in a particular order and are under the tight control of a signaling network of yeast quiescence, 3) specific genetic manipulations that alter the information flow down the program’s consecutive steps can accelerate or decelerate the program, and 4) akin to other programmed biological events, the cellular quiescence program is beneficial for the survival and stress resistance of a yeast cell’s population.

CR is a low-calorie diet without malnutrition that slows aging, extends longevity, and delays the onset of aging-associated pathologies in budding yeast and many other eukaryotic organisms [12]. Nutrient-rich complete media or nutrient-limited synthetic minimal media supplemented with 0.2% or 0.5% glucose are traditionally used to assess how CR delays budding yeast’s chronological aging [6, 7]. It has been shown that the use of a nutrient-rich complete medium supplemented with 0.2% or 0.5% glucose provides all nutrients needed for the growth and survival of budding yeast [13]. Moreover, the use of this medium is beneficial for studying the effects of CR on budding yeast’s chronological aging [2].

Percoll density gradient centrifugation was used to purify the Q and NQ cell populations from budding yeast cultures incubated in a nutrient-rich medium under non-CR [3] or CR [14] conditions. The properties of these cell populations were compared, and the effects of CR on Q cells’ properties were assessed [14]. As outlined below, these studies provided conclusive evidence that the CR-dependent changes of Q cells’ properties are essential contributors to the CR-driven slowdown of budding yeast’s chronological aging [14].

Under non-CR conditions, the budding yeast cells that arrest their cell cycle and enter a G_0_ state become a Q cell population [4]. In contrast, the budding yeast cells under the same culturing conditions do not undergo cell cycle arrest; these cells form three different populations of NQ cells [4]. The Q and NQ cell populations within non-CR cultures differ in their properties. Some properties of the Q cells in these cultures are well established [3, 4, 8–10]. The population of Q cells in non-CR cultures includes unbudded cells of a uniform diameter; these cells have a thick cell wall, refract light if examined by phase-contrast microscopy and exhibit a high buoyant density. Q cells under non-CR conditions are highly metabolically active. They stockpile significant amounts of glycogen and trehalose. The mitochondria in these Q cells are respiratory functional and exhibit a high membrane potential. The concentration of reactive oxygen species (ROS) and extent of oxidative macromolecular damage are low in Q cells under non-CR conditions. These Q cells exhibit both characteristic properties of quiescence. They maintain the clonogenicity (*i.e.*, the ability of a Q cell to form a colony on the surface of a nutrient-rich solid medium after being transferred from a nutrient-depleted liquid medium) and synchronously re-enter mitosis if transferred from a nutrient-depleted to a nutrient-rich liquid medium. Q cells under non-CR conditions are stress-resistant and exhibit a low DNA mutation rate. The aging-associated onsets of the apoptotic and necrotic forms of cell death are postponed in these cells.

Recent studies have shown that several heat-shock proteins move from the cytosol to the nucleus in Q cells under non-CR conditions [11]. Yet, other heat-shock proteins and various enzymes accumulate in the numerous cytosolic foci or filaments found in these Q cells [11]. Nuclear morphology, chromosome positioning in the nucleus and nuclear gene transcription undergo significant changes in the Q cells cultured under non-CR conditions [11]. Because these cells amass cytosolic P-bodies and stress granules, displace dysfunctional proteasome subunits from the nucleus to the insoluble protein deposits, and relocate functional proteasome subunits from the nucleus to the proteasome storage granules, they excel in maintaining cellular proteostasis [11]. Actin cytoskeleton and microtubules, including stable microtubules in the nucleus, undergo significant remodeling in Q cells under non-CR conditions [11]. Fragmentation of the dynamic mitochondrial network in these cells results in numerous small and globular mitochondria at the cell periphery [11].

A body of evidence indicates that some of the above properties of Q cells under non-CR conditions play essential roles in these cells’ abilities to exit a quiescent state, return to the cell cycle and restart proliferation [4, 11, 14]. Some of these properties are also essential for longevity assurance in chronologically aging budding yeast [3, 4, 8–11, 14]. It is presently unknown how Q cells under non-CR conditions control the establishment of the numerous properties described above. The mechanistic links between these properties and quiescence exit, cell cycle re-entry, proliferation reinstatement and longevity assurance remain determined.

Three NQ cell populations can be found in a budding yeast culture under non-CR conditions [3, 4, 8–10]. The properties of these NQ cell populations are described below.

NQ cell population 1 includes mainly the mother cells of the first and higher generations with one or more bud scars on their surface [4]. These bud scars appear on the mother cell’s surface after a new daughter cell (bud) separates from it [15]. NQ cells in this population exhibit a high rate of metabolism and are clonogenic [3, 4, 8–10]. The characteristic features of NQ cells in this population are their low buoyant density, reduced mitochondrial respiration, elevated ROS, and high frequencies of mutations in the nuclear and mitochondrial DNA [4].

NQ cell population 2 also contains various generations of mother cells that are metabolically active [3, 4, 8–10]. A characteristic feature of NQ cells in this population is that they are not clonogenic [4]. Therefore, it is commonly accepted that these NQ cells are descendants of NQ cells from population 1 [4].

NQ cell population 3 consists of cells that are not clonogenic [3, 4, 8–10]. Because these cells exhibit markers of apoptosis and necrosis, it is believed that their predecessors are NQ cells from population 2 [4].

The distinct features exhibited by NQ cells of populations 1, 2 and 3 suggest that the order of their stepwise conversion during chronological aging is NQ 1 → NQ 2 → NQ 3 [4, 16]. It is also tempting to speculate that NQ 1 cells are descendants of Q cells [4, 16]. It remains unclear how yeast’s chronological aging promotes Q cells’ conversion into NQ 1 cells.

The purification of the Q and NQ cells and their comparative analyses have been reported for budding yeast cultures that undergo chronological aging under CR conditions [14]. Below, we discuss how the low-calorie diet influences characteristic features exhibited by the Q and NQ cells and how it affects the age-related dynamics of their appearance during chronological aging in budding yeast.

Following glucose consumption by the yeast culture under non-CR conditions, cells enter the G_0_ state and give rise to the high-density Q cell population due to the cell cycle arrest in late G_1_ [3, 4, 8–10]. On the contrary, when the yeast culture under CR conditions consumes glucose, it enters the G_0_ state and develops the high-density Q cell population because the cell cycle arrest occurs in early G_1_ [14, 16, 17].

The percentage of low-density Q cells in the yeast culture under non-CR conditions reaches a maximum after the culture enters the ST growth phase [14]. In contrast, the yeast culture under CR conditions begins to accumulate the maximal percentage of low-density Q cells when it enters the post-diauxic growth phase [14].

CR affects both criteria of the chronological age-related quiescence decline by the populations of Q and NQ cells. Indeed, CR extends the time these cell populations retain their clonogenicity [14, 16, 17]. Besides, CR slows a chronological aging-associated decline in Q and NQ cells’ ability to synchronously divide if transferred from a nutrient-depleted to a nutrient-rich liquid medium [14, 16, 17]. CR increases glycogen and trehalose concentrations in both Q and NQ cells [14]. The high molecular mass branched polysaccharide glycogen and the nonreducing disaccharide trehalose store glucose in budding yeast cells [18]. Trehalose also maintains proteostasis in budding yeast cells and protects these cells from chronic stress-inflicted death [19].

CR causes a substantial decline in the neutral lipids triacylglycerols (TAG) concentrations within Q and NQ cells [14]. After the synthesis of TAG in the endoplasmic reticulum (ER), they are deposited in lipid droplets (LDs) [20]. TAG stored in LDs provide free (non-esterified) fatty acids that can be oxidized to produce energy or used to synthesize various phospholipids [20]. CR elicits a considerable rise in cardiolipins (CL) concentrations within both Q and NQ cells [14]. CL are synthesized and reside in the inner mitochondrial membrane (IMM) [20]. These diphosphatidylglycerol lipids are essential contributors to mitochondrial morphology and function [20].

CR stimulates mitochondrial respiration and increases the electrochemical potential across the inner mitochondrial membrane (ΔΨm) in both Q and NQ cells [14]. Both mitochondrial respiration and ΔΨm are known to play essential roles in regulating the longevity of chronologically aging yeast [21, 22]. CR decreases cellular ROS concentrations in chronologically “young” cells that did not enter the ST growth phase and increases these concentrations in chronologically “old” cells that enter the ST phase [14]. These effects of CR on cellular ROS concentrations are observed in both Q and NQ cells [14]. ROS are by-products of mitochondrial respiration [23] that play essential roles in the longevity assurance of budding yeast [2, 6, 21–24].

CR reduces the extent of oxidative damage to cellular macromolecules (including proteins, lipids and DNA) during chronological aging of both Q and NQ cells [14]. This type of macromolecular damage is an essential contributor to budding yeast’s chronological aging [21, 22, 24, 25].

CR makes both Q and NQ cells more resistant to chronic thermal and oxidative stresses [14]. A rise in yeast cells’ resistance to these stresses contributes to the delay of budding yeast’s chronological aging [2, 6, 21, 22, 24].

CR delays the chronological age-related onset of the apoptotic and necrotic forms of regulated cell death (RCD) in both Q and NQ cells [14]. CR also makes both Q and NQ cells more resistant to the apoptotic and necrotic forms of RCD inflicted by exogenous chemical interventions [14]. These RCD forms are known to terminate the life of chronologically “old” budding yeast cells [6, 26–28].

**Figure 1:**
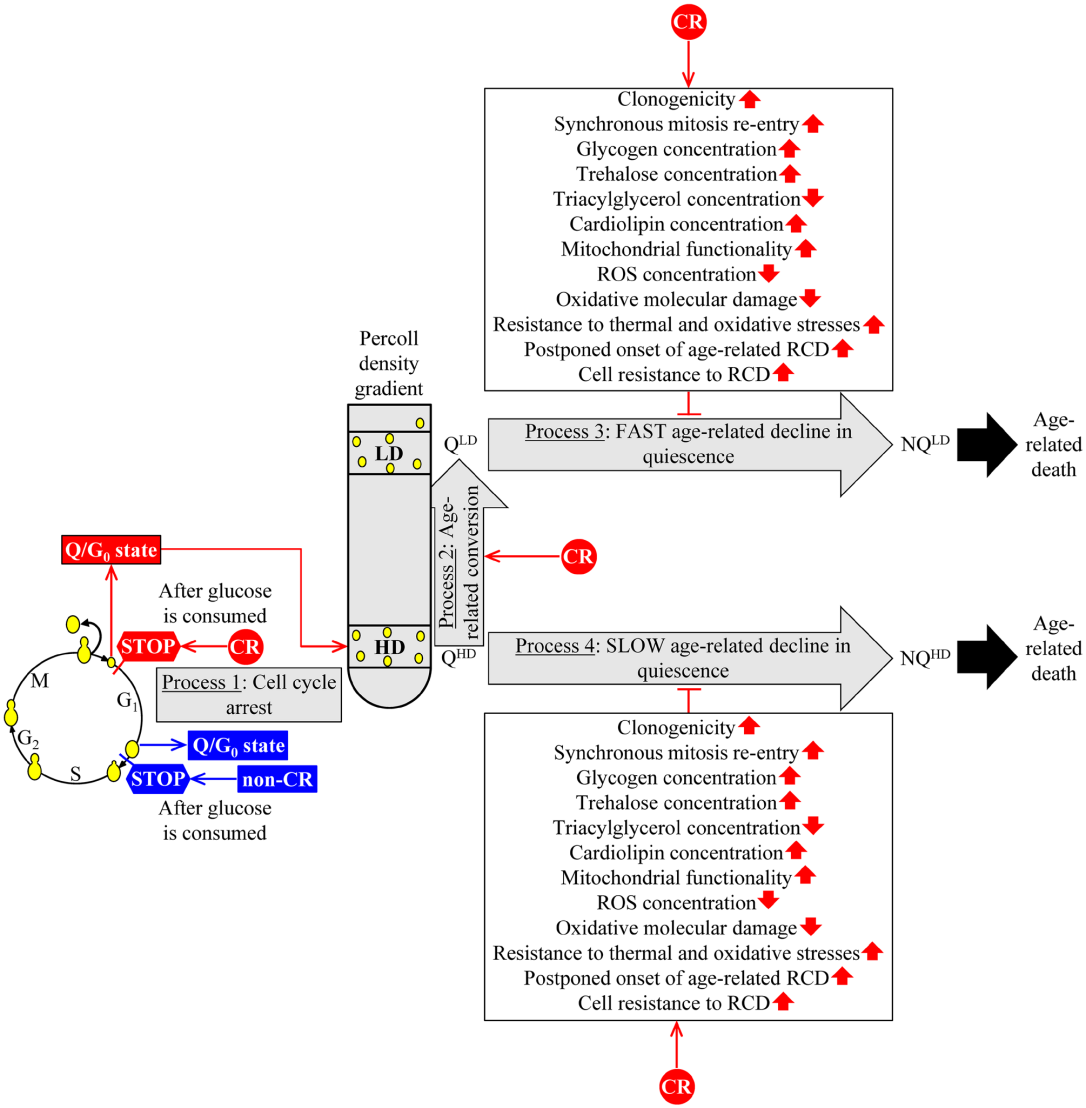
A hypothetical mechanism through which CR could slow yeast chronological aging by affecting the characteristic features of Q cells and their age-related dynamics. The proposed mechanism’s central tenet is that the CR-dependent delay of yeast chronological aging is due to the low-calorie diet’s ability to affect four essential processes within Q cells. More details are provided in the text. Abbreviations: HD: high-density cells; LD: low-density cells; Q^HD^: quiescent cell of high density; Q^LD^: quiescent cell of low density; NQ^HD^: non-quiescent cell of high density; NQ^LD^: non-quiescent cell of low density; RCD: regulated cell death; ROS, reactive oxygen species. ↑ Increased by CR; ↓ Decreased by CR; ˧ Slows the conversion of Q^LD^ cells into NQ^LD^ cells or the Q^HD^-into-NQ^HD^ cell conversion.

Based on our analysis of how CR influences the properties of high- and low-density Q cells and their age-related conversion into high- and low-density NQ cells, we proposed a hypothesis on the mechanism by which CR could decelerate the chronological aging of budding yeast because it influences the characteristic features of Q cells and their aging-associated dynamics [14, 16, 17]. This hypothesis is briefly discussed below and schematically depicted in Figure 1. The central tenet of the hypothesis is that the CR-dependent delay of yeast chronological aging is due to the low-calorie diet’s ability to affect several essential processes within Q cells.

CR arrests the cell cycle in early G_1_ and creates small Q^HD^ cells (Figure 1, process 1). The hypothesis posits that these small Q^HD^ cells exhibit the improved pro-longevity features named in Figure 1. These features include an increase in Q cell’s clonogenicity (*i.e.*, the ability of a Q cell to form a colony on the surface of a nutrient-rich solid medium after being transferred from a nutrient-depleted liquid medium), a rise in Q cell’s ability to synchronously re-enter mitosis if transferred from a nutrient-depleted liquid medium to a nutrient-rich liquid medium, a decline in TAG concentration inside of a Q cell, an elevation in CL concentration within a Q cell and others.

CR accelerates Q^HD^ cells’ conversion into Q^LD^ cells during budding yeast’s chronological aging (Figure 1, process 2). The hypothesis suggests that the accelerated Q^HD^-into-Q^LD^ transformation could contribute to the development of improved pro-longevity features termed in Figure 1.

CR slows a fast aging-associated decline in quiescence, *i.e.*, the losses of clonogenicity and synchronous cell cycle re-entry, resulting in converting long-lived Q^LD^ cells into short-lived NQ^LD^ cells (Figure 1, process 3). The hypothesis postulates that the decelerated Q^LD^-into-NQ^LD^ conversion could be an essential contributor to improving pro-longevity features named in Figure 1.

CR decelerates a slow aging-associated deterioration in quiescence, *i.e.*, the losses of clonogenicity and synchronous cell cycle re-entry leading to a transformation of long-lived Q^LD^ cells into short-lived NQ^LD^ cells (Figure 1, process 4). The hypothesis posits that by decelerating the Q^LD^-into-NQ^LD^ conversion, CR could advance the enhanced pro-longevity features mentioned in Figure 1.

**Figure 2:**
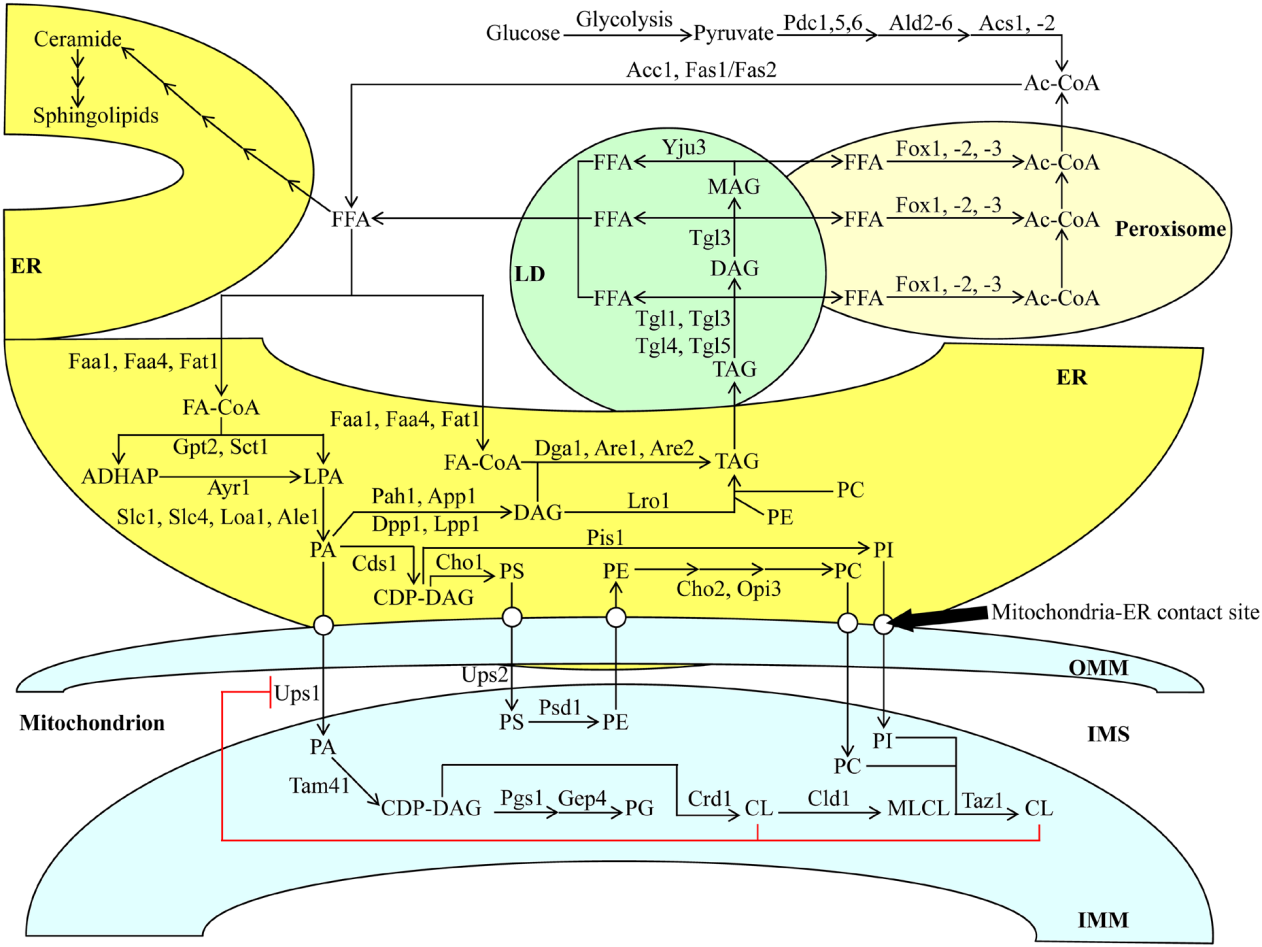
Metabolic and interorganellar transport processes catalyzed by enzymes residing in the cytosol, ER, mitochondria, LDs, and peroxisomes alter the concentrations of various lipid classes in yeast cells. A T-bar in red depicts the CL-dependent negative feedback on the transport of PA from the ER to the IMM. See text for further details. Other abbreviations not specified in the text include: Ale1, acyltransferase for lysophosphatidylethanolamine 1; App1, actin patch protein 1; Are1/2, acyl-coenzyme A: cholesterol acyl transferase-related enzymes 1 and 2; Ayr1, acyl-dihydroxyacetone-phosphate reductase 1; Cds1, CDP-diacylglycerol synthase 1; Cho1/2, choline requiring 1 and 2; Cld1, cardiolipin-specific deacylase 1; Crd1, cardiolipin synthase 1; Dga1, diacylglycerol acyltransferase 1; Dpp1, diacylglycerol pyrophosphate phosphatase 1; Faa1 and Faa4, fatty acid activation proteins 1 and 4; Fas1 and Fas2, fatty acid synthetases 1 and 2; Fat1, fatty acid transporter 1; Fox1, Fox2 and Fox3, fatty acid oxidation proteins 1, 2 and 3; Gep4, genetic interactor of prohibitins protein 4; Gpt2, glycerol-3-phosphate acyltransferase; Loa1, lysophosphatidic acid: oleoyl-CoA acyltransferase 1; Lpp1, lipid phosphate phosphatase 1; Lro1, lecithin cholesterol acyl transferase related open reading frame 1; Opi3, overproducer of inositol 3; Pah1, phosphatidic acid phosphohydrolase 1; Pgs1, phosphatidylglycerolphosphate synthase 1; Pis1, phosphatidylinositol synthase 1; Psd1, phosphatidylserine decarboxylase 1; Sct1, suppressor of choline-transport mutants 1; Slc1 and Slc4, sphingolipid compensation proteins 1 and 4; Tam41, translocator assembly and maintenance protein 41; Taz1, tafazzin protein 1; Tgl1, Tgl3, Tgl4, Tgl5, triglyceride lipases 1, 3, 4 and 5; Ups1 and Ups2, unprocessed proteins 1 and 2; Yju3, monoglyceride lipase.

As mentioned above, the CR diet elicits a considerable decline in TAG concentration and a significant rise in CL concentration within HD and LD cells of the Q and NQ types (Figure 1; [14]). The intracellular concentrations of TAG and CL (as well as other lipid classes) depend on the intensity of metabolite flow throughout an elaborate network of pathways for lipid metabolism and interorganellar transport. This intricate network has been intensively reviewed [20, 29–32]. It is schematically depicted in Figure 2 and briefly described below.

If glucose is the only carbon source exogenously added to yeast cultures, it is initially converted to pyruvate in the cytosol via the ten-step glycolytic pathway (Figure 2). Pyruvate is then used to synthesize acetyl-CoA (Ac-CoA) via three consecutive reactions catalyzed by the following enzymes: 1) the cytosolic pyruvate decarboxylase isozymes Pdc1, 5 and 6, 2) the aldehyde dehydrogenases Ald2 and 6, and 3) the Ac-CoA synthetase isoforms Acs1 and 2 (Figure 2). Peroxisomal reactions also contribute to the cytosolic pool of Ac-CoA through free fatty acid (FFA) β-oxidation via Fox1-, 2- and 3-dependent chemical reactions (Figure 2). Following its synthesis, Ac-CoA acts as a substrate for FFA formation in the cytosolic via the Ac-CoA carboxylase Acc1 and fatty acid synthase complex Fas1/Fas2 (Figure 2). FFA can also be produced in LDs via the hydrolysis of TAG by the Tgl1, 3, 4 and 5 lipases and the lipolytic degradation of diacylglycerols (DAG) and monoacylglycerols (MAG), both of which are TAG-derived. Such processes are carried out by the lipases Tgl3 and Yju3, respectively, in LDs (Figure 2). Following the formation of FFA via the processes mentioned above, a portion of FFA is used for the synthesis of ceramides (CER) and then sphingolipids (SPH) in the ER (Figure 2).

The long-chain acyl-CoA synthetases Faa1, Faa4 and Fat1 in the ER catalyze reactions that convert an FFA pool outside of the ER into fatty acyl-CoA esters (FA-CoA) (Figure 2). The latter molecule is then used for the *de novo* synthesis of TAG, CL and glycerophospholipids (including phosphatidic acid (PA), phosphatidylserine (PS), phosphatidylethanolamine (PE), phosphatidylcholine (PC) and phosphatidylinositol (PI)) via enzymes confined to the ER and the mitochondria (Figure 2). This process begins in the ER, where glycerol-3-phosphate (G3P)/dihydroxyacetone phosphate acetyltransferases Sct1 and Gpt2 catalyze the formation of lysophosphatidic acid (LPA) or acyl-dihydroxyacetone phosphate (ADHAP) from FA-CoA and G3P or DHAP, respectively (Figure 2). ADHAP is also converted to LPA via an Ayr1-driven reaction (Figure 2). LPA formed from FA-CoA or ADHAP can then be converted to PA via the LPA acyltransferases Slc1, Slc4, Loa1 and Ale1 (Figure 2).

After PA synthesis in the ER, PA can enter the following three branches of lipid metabolite flow. First, a Cds1-driven reaction can convert PA into cytidine diphosphate (CDP)-DAG, a common precursor for other phospholipids (Figure 2). As such, a Pis1-dependent synthesis of PI from this precursor can occur in the ER (Figure 2). Alternatively, a Cho1-dependent reaction can convert CDP-DAG to PS (Figure 2). The latter can be shuttled to the outer mitochondrial membrane (OMM) through the mitochondria-ER contact sites and subsequently through the intermembrane space (IMS) to the IMM via Ups2-driven transport (Figure 2). PE is synthesized by Psd1 from PS within the IMM and is then shuttled back to the ER (Figure 2). A Cho2- and Opi3-dependent synthesis of PC occurs in the ER from the shuttled PE (Figure 2). Second, PA can also be converted to DAG in a reaction catalyzed by the PA phosphatases Pah1, App1, Dpp1 and Lpp1 in the ER (Figure 2). Subsequently, DAG can be acylated to TAG via an FA-CoA-dependent reaction driven by Dga1, Are1 and Are2, and in a PE- and PC-dependent catalyzed by Lro1 (Figure 2). Following the *de novo* synthesis of TAG in the ER, these molecules can be deposited in LDs and broken down into FFA, as mentioned above (Figure 2). Third, PA can be transported from the ER to the IMM similarly to PS, this time with the help of Ups1 (Figure 2). Within the IMM, PA can be converted into CDP-DAG using Tam41 (Figure 2). Then, CDP-DAG can either be converted into phosphatidylglycerol (PG) using the Pgs1 and Gep4 enzymes, respectively or into CL and then monolysocardiolipin (MLCL) via Crd1 and Cld1, respectively (Figure 2). PC can also be converted to CL in the IMM via the Taz1 enzyme (Figure 2). Finally, the accumulation of CL in the IMM creates a negative feedback loop on the Ups1-dependent transport of PA into the IMM from the ER, ultimately inhibiting the buildup of PA in the mitochondria (Figure 2).

The studies described here had the following two objectives. First, we sought to investigate how CR influences the concentrations of all lipid classes (not only TAG and CL concentrations) within HD and LD cells of the wild-type (WT) strain on different stages of the chronological aging process. As we mentioned earlier, an HD cell population contains both Q and NQ cells; the percentage of NQ cells in the HD cell population slowly increases during chronological aging [14, 16, 17]. An LD cell population also contains both Q and NQ cells; however, the percentage of NQ cells in the LD cell population increases quicker during chronological aging than in the HD cell population [14, 16, 17]. To attain our first objective, we used Percoll density gradient centrifugation to purify the HD and LD cell populations from WT yeast cultured under CR or non-CR conditions and recovered on different days of culturing. We then employed liquid chromatography coupled with tandem mass spectrometry (LC-MS/MS) to identify and quantify various lipid classes in these cell populations. Noteworthy, our study is the first attempt to assess the dynamics of changes in the entire lipidome of Q and NQ cells at different stages of the chronological aging process. Therefore, it was unclear if these age-related changes in the lipidomes of Q and NQ cells are permanent through the chronological aging process or there are some critical periods of the aging process at which the specific changes occur.

Our second objective was to compare the effects of CR, the *tor1Δ* mutation and lithocholic acid (LCA) on the lipidomes of the HD and LD cell populations at different stages of chronological aging. These geroprotectors significantly increase budding yeast’s chronological lifespan (CLS) [33]. There were three reasons for choosing CR, the *tor1Δ* mutation and LCA for these comparative studies. The first reason was that these three geroprotectors differ from each other in applying them to slow budding yeast’s chronological aging in a nutrient-rich complete medium. Indeed, CR is a dietary geroprotective intervention [2], the *tor1Δ* mutation is a genetic geroprotective intervention [34] and LCA is a pharmacological geroprotective intervention [34]. The second reason was that these three geroprotectors delay aging by targeting different cellular processes. CR is a geroprotective dietary regimen that slows budding yeast’s chronological aging by affecting carbohydrate and lipid metabolism, protein import into various organelles, different aspects of mitochondrial functionality, protein synthesis in the cytosol and mitochondria, cellular proteostasis, autophagic degradation of aged, dysfunctional and damaged macromolecules and organelles, cell resistance to various chronic (long-lasting) stresses, and cell susceptibility to the apoptotic and necrotic forms of RCD [6, 7]. The *tor1Δ* mutation inactivates the TORC1 protein complex; TORC1 is known to accelerate budding yeast’s chronological aging by influencing protein synthesis in the cytosol and mitochondria, autophagic degradation of aged, dysfunctional and damaged macromolecules and organelles, and cell resistance to different chronic stresses [6, 7]. LCA is a pharmacological geroprotective agent that accumulates in the inner and outer mitochondrial membranes and alters mitochondria’s number, size, and functional state [35, 36]. The third reason for choosing CR, the *tor1Δ* mutation and LCA for these studies was that LCA delays budding yeast’s chronological aging and extends longevity significantly more efficiently than the two other geroprotective interventions [33]. Thus, it was plausible that LCA affects the lipidomes of the HD and LD cell populations differently than the two other geroprotectors.

The effects of CR, the *tor1Δ* mutation and LCA on the cellular lipidome were studied in budding yeast cultured in the nutrient-rich YP (1% yeast extract and 2% peptone) medium supplemented with glucose as a sole carbon source. As discussed earlier, the use of this medium is advantageous for studying the effects of CR on budding yeast’s chronological aging and makes this yeast a beneficial model system for elucidating the chronological aging of multicellular eukaryotes [2]. A WT strain culture in the YP medium initially containing 2% (w/v) glucose served as a control non-CR culture, whereas a WT strain culture in the same medium initially containing 0.2% (w/v) glucose was used as a model system to study how CR affects the cellular lipidome. The effect of the *tor1Δ* mutation on the cellular lipidome was examined in the mutant yeast cells cultured in the YP medium initially containing 2% (w/v) glucose; under these cell culturing conditions, the *tor1Δ* mutation exhibits the highest longevity-extending effect [2]. The effect of LCA on the cellular lipidome was assessed in the WT strain cultured in the YP medium initially containing 0.2% (w/v) glucose and 50 μM LCA; under these cell culturing conditions, LCA exhibits the highest beneficial effect on budding yeast’s CLS [34].

In studies described here, we showed that CR changes the concentrations of many different lipid classes through most of the CLS of the HD and LD populations of Q and NQ cells. TAG concentrations are lowered, FFA concentrations are raised, phospholipid concentrations are increased and CL concentrations are enlarged by CR. These findings predict a hypothetical model for a CR-driven reorganization of lipid metabolism and transport in the ER, LDs and mitochondria of the Q and NQ cells. Our comparative analysis of the effects of CR, the tor1Δ mutation and LCA on the lipidomes of the HD and LD populations of Q and NQ cells provided the first evidence that the lipidomic patterns of these cell populations established by CR differ from those created by the two other geroprotectors.

## RESULTS

### Only CR, but not the *tor1Δ* mutation or LCA, decreases TAG concentration in HD and LD cells through most of the chronological lifespan

**Figure 3:**
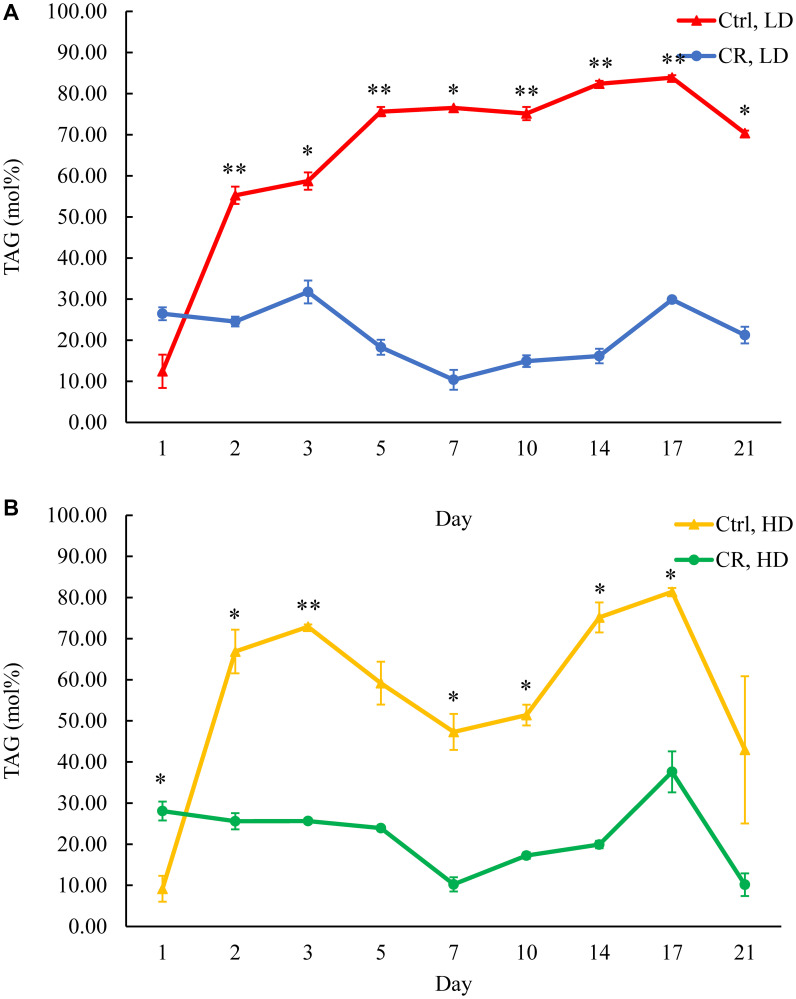
CR decreases TAG concentration in both HD and LD cells through the most chronological lifespan. Samples of WT yeast cultured in YP medium initially containing 0.2% glucose (CR conditions) or 2% glucose (control non-CR conditions) were recovered on different days of culturing and subjected to centrifugation in Percoll density gradient to purify HD and LD cell populations. TAG concentrations were measured by LC-MS/MS. TAG concentrations in LD (**A**) and HD (**B**) cells are shown. Data are presented as means ± SD (*n* = 2; ^*^ *p* < 0.05; ^**^ *p* < 0.01). Abbreviation: Ctrl: control.

The previous study has shown that CR reduces TAG concentration in HD and LD cell populations through the most chronological lifespan [14]. A mass spectrometric quantitative assessment of the yeast lipidome in these experiments was performed using the direct-injection method [14]. We recently developed a novel LC-MS/MS method of quantitative lipidomics [37]. Using this novel method, we confirmed that CR lowers TAG concentration in both HD and LD cells through most of the chronological lifespan (Figure 3A and 3B), except for day 1 of culturing. Yet, neither the *tor1Δ* mutation nor LCA elicited long-lasting changes in TAG concentrations within HD or LD cells (Supplementary Figures 1A, 1B, 2A and 2B, respectively). Of note, the *tor1Δ* mutation increased TAG concentration in HD cells between days 5 and 10 of cell culturing (Supplementary Figure 1B), whereas LCA decreased TAG concentration in HD cells between days 10 and 17 of cell culturing (Supplementary Figure 2B).

Based on these observations, we concluded that, while CR creates a continuing trend of decreasing TAG concentration in both HD and LD cells through the most chronological lifespan, neither the tor1Δ mutation nor LCA exhibits a similar long-lasting effect on TAG concentration. Future research will address the importance of a temporary TAG rise in HD cells carrying the *tor1Δ* mutation (Supplementary Figure 1B). The significance of a temporary decline in TAG concentration within HD cells of WT treated with LCA remains unclear (Supplementary Figure 2B).

### Only CR, but not the *tor1Δ* mutation or LCA, increases FFA concentration in HD and LD cells through most of the chronological lifespan

**Figure 4:**
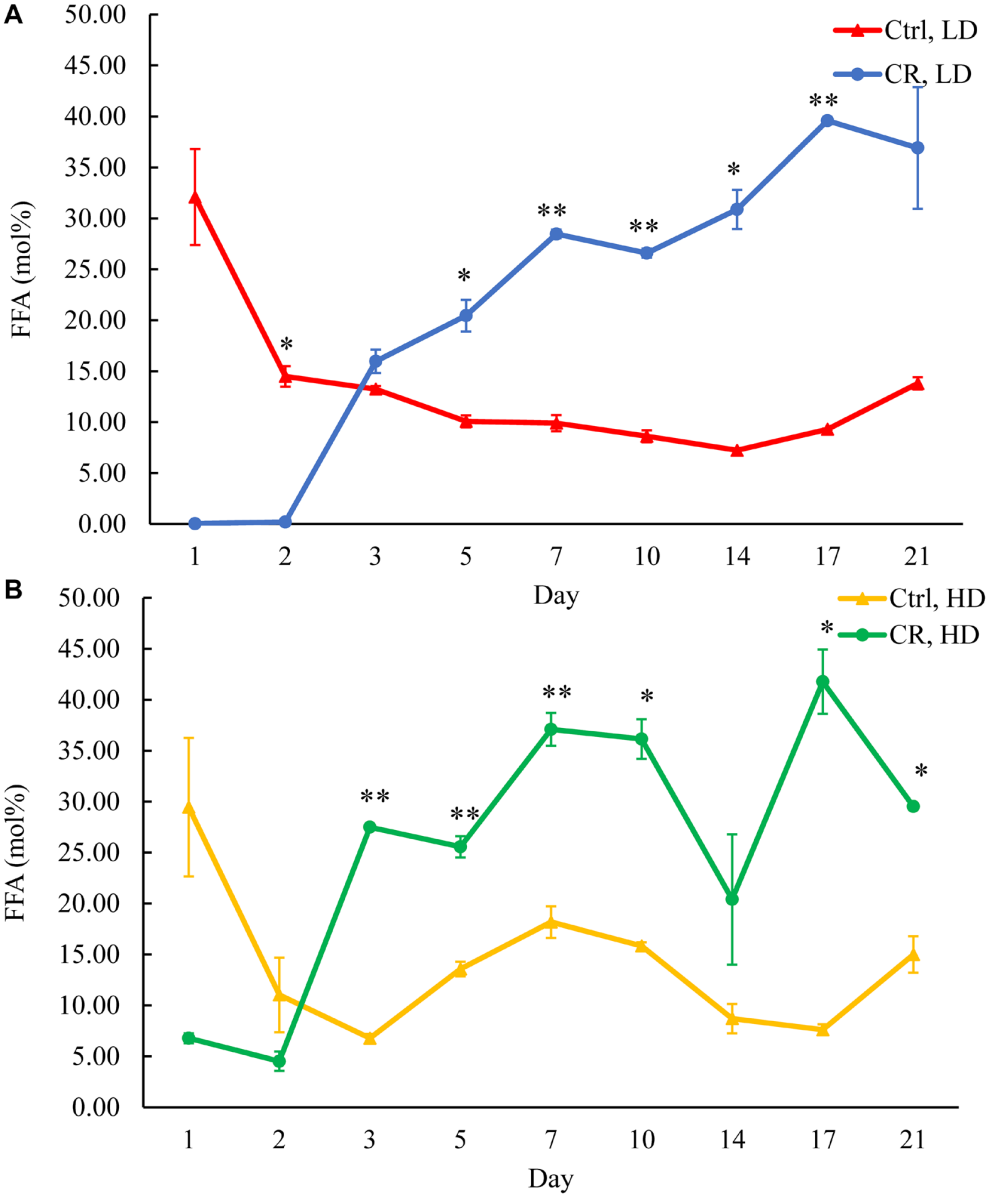
CR increases FFA concentration in HD and LD cells after day 2 of the chronological lifespan. Samples of WT yeast cultured in YP medium initially containing 0.2% glucose (CR conditions) or 2% glucose (control non-CR conditions) were recovered on different days of culturing and subjected to centrifugation in Percoll density gradient to purify HD and LD cell populations. FFA concentrations were measured by LC-MS/MS. FFA concentrations in LD (**A**) and HD (**B**) cells are shown. Data are presented as means ± SD (*n* = 2; ^*^ *p* < 0.05; ^**^ *p* < 0.01). Abbreviation: Ctrl: control.

One way of decreasing TAG concentration in HD and LD cells under CR conditions is by stimulating TAG lipolysis within LDs (Figure 2). The stimulation of TAG lipolysis alone results in a rise of FFA (Figure 2). Suppose such stimulation of TAG lipolysis and FFA formation within LDs does not coincide with a change in FFA oxidation rate within peroxisomes. In that case, TAG lipolysis stimulation is expected to increase the intracellular concentration of FFA (Figure 2). Indeed, we observed a rise in FFA concentration within HD and LD cells under CR conditions after day 2 of the chronological lifespan (Figure 4). Thus, CR intensifies TAG lipolysis in LDs without changing the rate of FFA oxidation in peroxisomes (Figure 2). The *tor1Δ* mutation decreased FFA concentration in HD cells between days 5 and 10 of cell culturing (Supplementary Figure 3A and 3B). Moreover, although LCA increased FFA concentration in LD cells and altered FFA concentration in HD cells through most of the chronological lifespan, LCA treatment effects on FFA concentration within HD and LD cells were not as dramatic as the ones for CR (Supplementary Figure 4A and 4B).

Based on these observations, we concluded that, while CR creates a long-lasting trend of rising FFA concentration in both HD and LD cells through the most chronological lifespan, neither the *tor1Δ* mutation nor LCA shows a similar continuing effect on FFA concentration.

### Neither CR, the *tor1Δ* mutation nor LCA has a long-lasting effect on DAG concentration in HD and LD cells through the chronological lifespan

The other way of decreasing TAG concentration in HD and LD cells under CR conditions is by lowering TAG synthesis from DAG within the ER (Figure 2). CR did not cause any statistically significant change in DAG concentration (neither in HD cells nor LD cells) through the chronological lifespan (Supplementary Figure 5). Therefore, we concluded that deterioration of TAG synthesis from DAG does not elicit the observed long-term decline in TAG concentration within these cells under CR conditions. Notably, neither the *tor1Δ* mutation nor LCA caused a significant continuing effect on DAG concentration in HD and LD cells (Supplementary Figures 6 and 7, respectively).

### Only CR, but not the *tor1Δ* mutation or LCA, increases CER concentration in LD cells through the entire chronological lifespan

**Figure 5:**
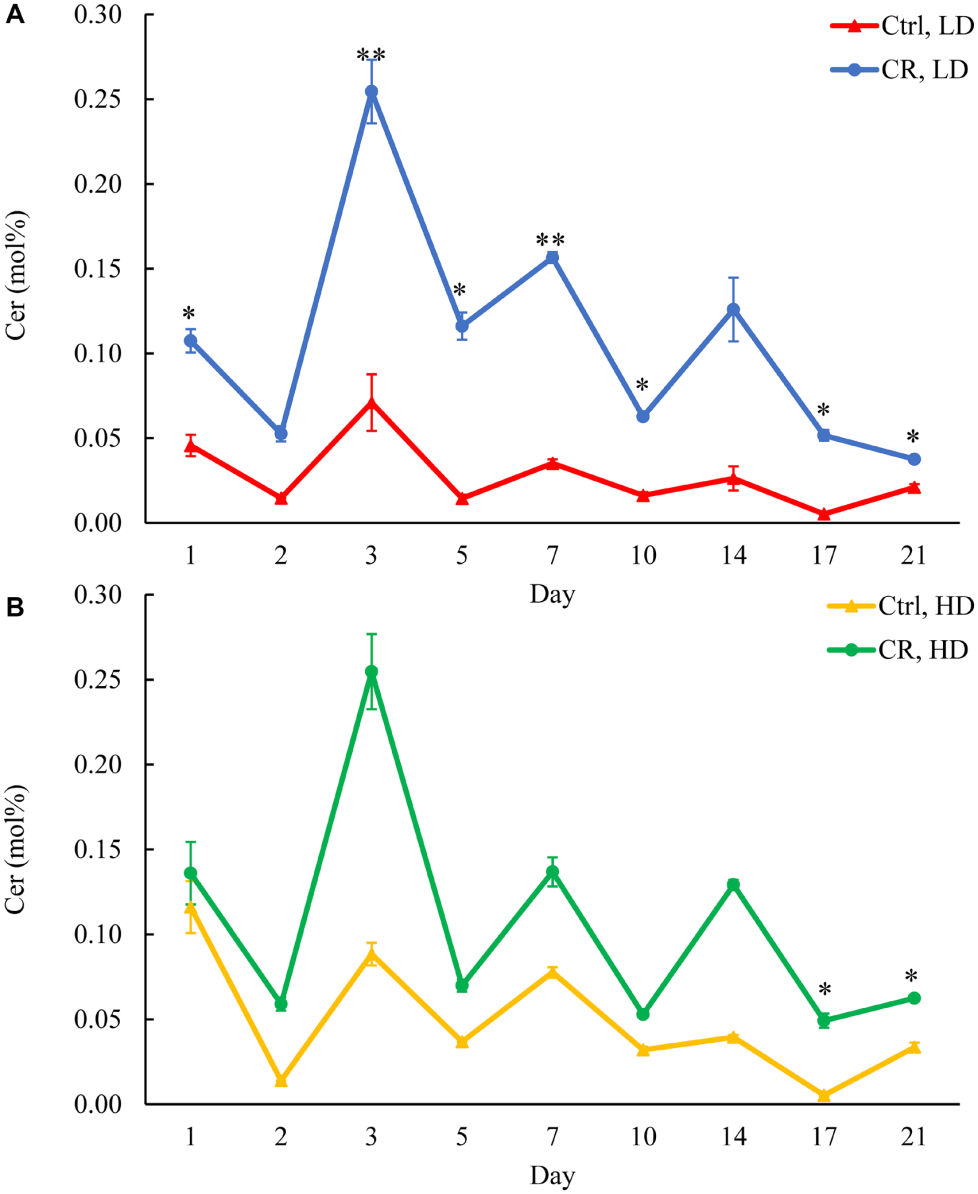
CR increases CER concentration in LD cells through the entire chronological lifespan. Samples of WT yeast cultured in YP medium initially containing 0.2% glucose (CR conditions) or 2% glucose (control non-CR conditions) were recovered on different days of culturing and subjected to centrifugation in Percoll density gradient to purify HD and LD cell populations. CER concentrations were measured by LC-MS/MS. CER concentrations in LD (**A**) and HD (**B**) cells are shown. Data are presented as means ± SD (*n* = 2; ^*^ *p* < 0.05; ^**^ *p* < 0.01). Abbreviation: Ctrl: control.

To assess the flow of the excessive amounts of FFA accumulating in HD and LD cells under CR conditions, we analyzed how CR influences CER concentrations in these cells. CER is formed in the ER from fatty acyl-CoA esters of palmitic acid and serine [38]. We found that CR increases CER concentration in LD cells through the chronological lifespan (Figure 5A). CR also raised CER concentration in HD cells through the chronological lifespan, but this rise was not statistically significant (Figure 5B). In contrast, the *tor1Δ* mutation decreased CER concentration in HD cells through the most chronological lifespan (Supplementary Figure 8A and 8B). LCA exhibited a similar effect on CER concentration in LD cells (Supplementary Figure 9A and 9B).

### Neither CR, the *tor1Δ* mutation nor LCA has a long-lasting effect on the concentrations of complex SPH in HD and LD cells through the chronological lifespan

SPH are the lipids generated in the ER from CER [38]. We found that, although CR significantly increases CER concentration in LD cells, this low-calorie diet does not considerably affect SPH concentrations in HD and LD cells through the chronological lifespan (Supplementary Figure 10). Noteworthy, neither the *tor1Δ* mutation nor LCA caused a significant long-lasting effect on SPH concentrations in HD and LD cells (Supplementary Figures 11 and 12, respectively).

### Only CR, but not the *tor1Δ* mutation or LCA, increases the concentrations of all ER- and mitochondria-synthesized phospholipids in HD and LD cells through most of the chronological lifespan

**Figure 6:**
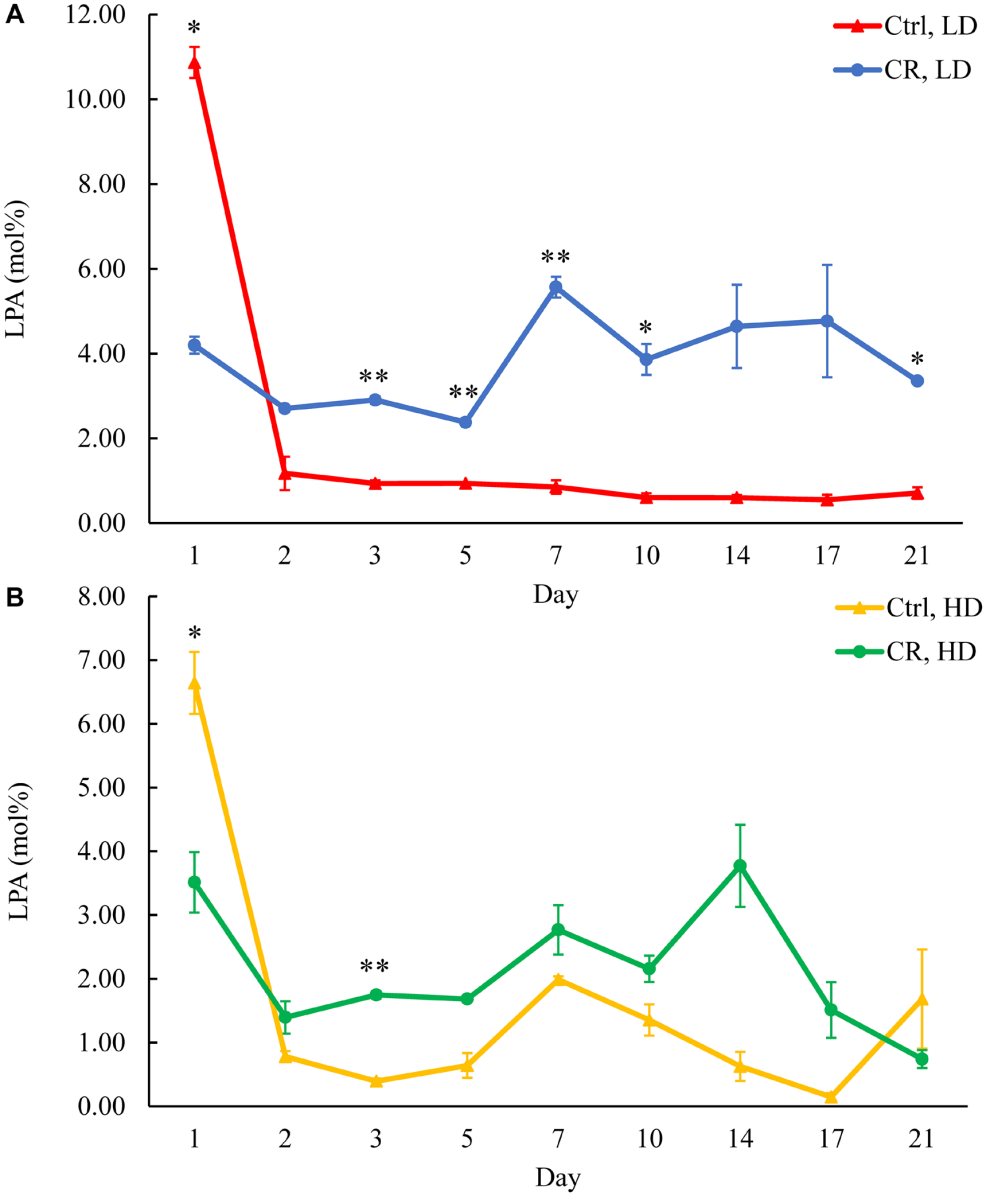
CR increases LPA concentration in HD and especially in LD cells after day 1 of the chronological lifespan. Samples of WT yeast cultured in YP medium initially containing 0.2% glucose (CR conditions) or 2% glucose (control non-CR conditions) were recovered on different days of culturing and subjected to centrifugation in Percoll density gradient to purify HD and LD cell populations. LPA concentrations were measured by LC-MS/MS. LPA concentrations in LD (**A**) and HD (**B**) cells are shown. Data are presented as means ± SD (*n* = 2; ^*^ *p* < 0.05; ^**^ *p* < 0.01). Abbreviation: Ctrl: control.

**Figure 7:**
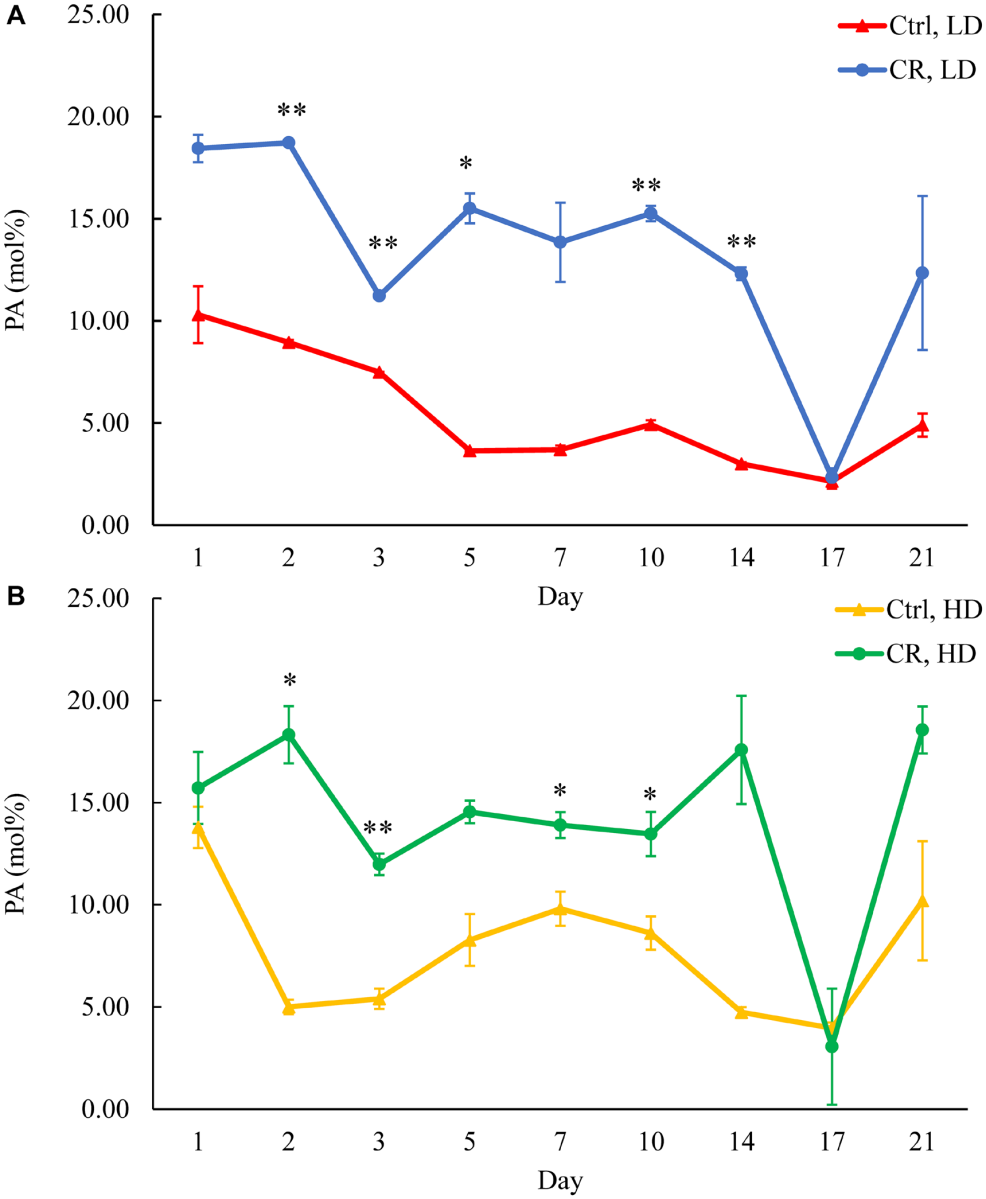
CR rises PA concentration in both HD and LD cells through the chronological lifespan. Samples of WT yeast cultured in YP medium initially containing 0.2% glucose (CR conditions) or 2% glucose (control non-CR conditions) were recovered on different days of culturing and subjected to centrifugation in Percoll density gradient to purify HD and LD cell populations. PA concentrations were measured by LC-MS/MS. PA concentrations in LD (**A**) and HD (**B**) cells are shown. Data are presented as means ± SD (*n* = 2; ^*^ *p* < 0.05; ^**^ *p* < 0.01). Abbreviation: Ctrl: control.

**Figure 8:**
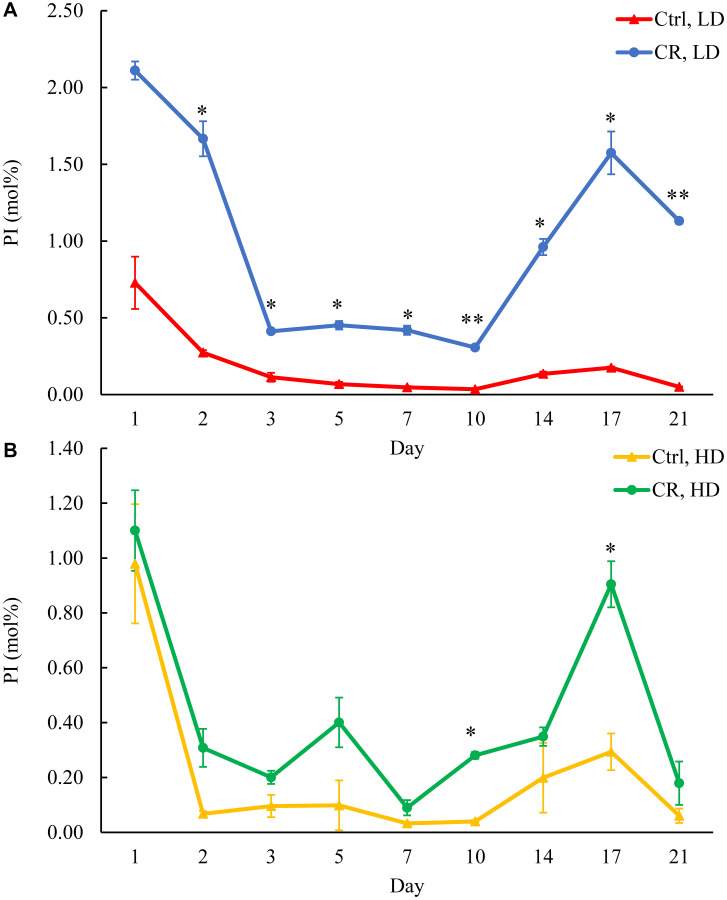
CR increases PI concentration in HD and especially in LD cells through the entire chronological lifespan. Samples of WT yeast cultured in YP medium initially containing 0.2% glucose (CR conditions) or 2% glucose (control non-CR conditions) were recovered on different days of culturing and subjected to centrifugation in Percoll density gradient to purify HD and LD cell populations. PI concentrations were measured by LC-MS/MS. PI concentrations in LD (**A**) and HD (**B**) cells are shown. Data are presented as means ± SD (*n* = 2; ^*^ *p* < 0.05; ^**^ *p* < 0.01). Abbreviation: Ctrl: control.

**Figure 9:**
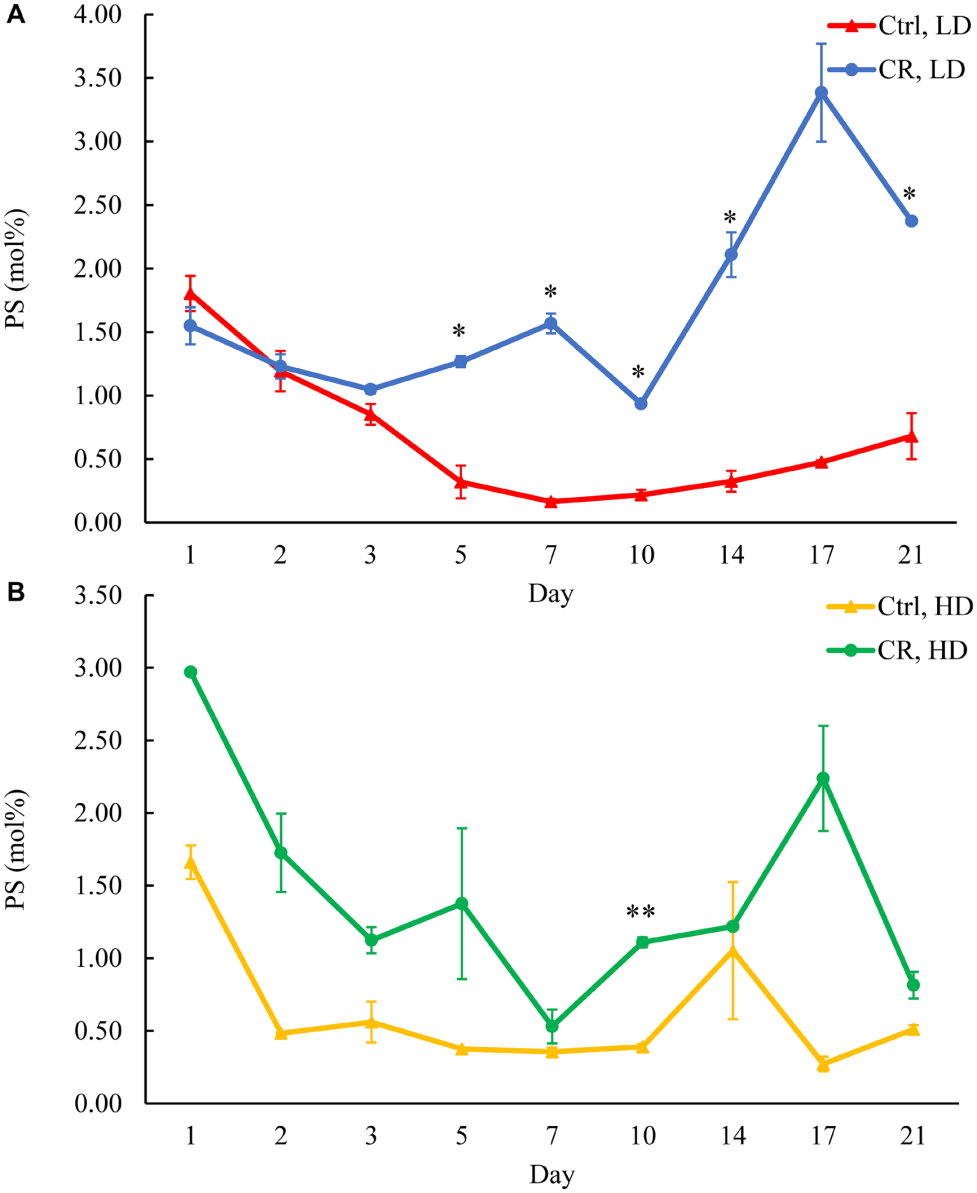
CR rises PS concentration in HD and especially in LD cells throughout the chronological lifespan. Samples of WT yeast cultured in YP medium initially containing 0.2% glucose (CR conditions) or 2% glucose (control non-CR conditions) were recovered on different days of culturing and subjected to centrifugation in Percoll density gradient to purify HD and LD cell populations. PS concentrations were measured by LC-MS/MS. PS concentrations in LD (**A**) and HD (**B**) cells are shown. Data are presented as means ± SD (*n* = 2; ^*^ *p* < 0.05; ^**^ *p* < 0.01). Abbreviation: Ctrl: control.

**Figure 10:**
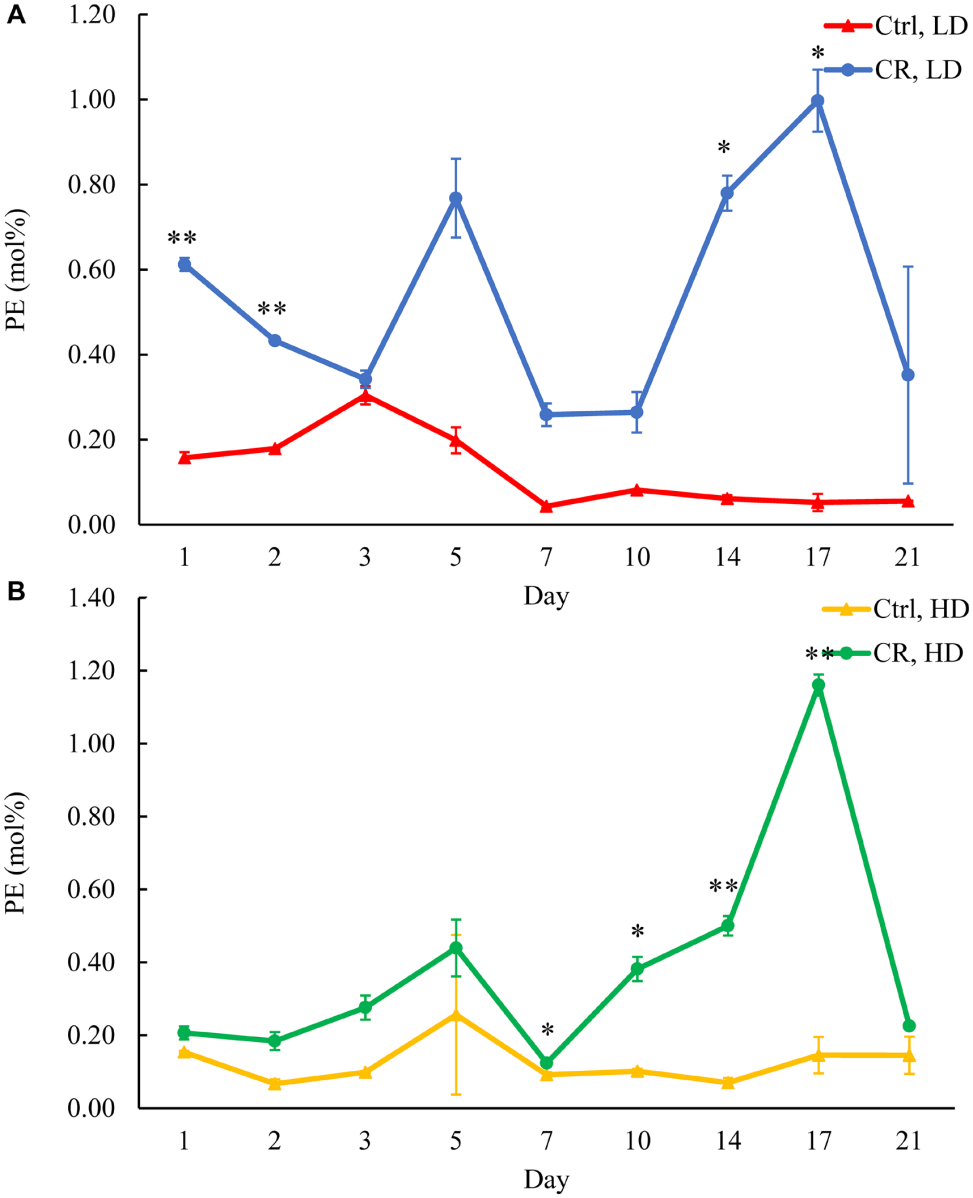
CR rises PE concentration in both HD and LD cells through the chronological lifespan. Samples of WT yeast cultured in YP medium initially containing 0.2% glucose (CR conditions) or 2% glucose (control non-CR conditions) were recovered on different days of culturing and subjected to centrifugation in Percoll density gradient to purify HD and LD cell populations. PE concentrations were measured by LC-MS/MS. PE concentrations in LD (**A**) and HD (**B**) cells are shown. Data are presented as means ± SD (*n* = 2; ^*^ *p* < 0.05; ^**^ *p* < 0.01). Abbreviation: Ctrl: control.

**Figure 11:**
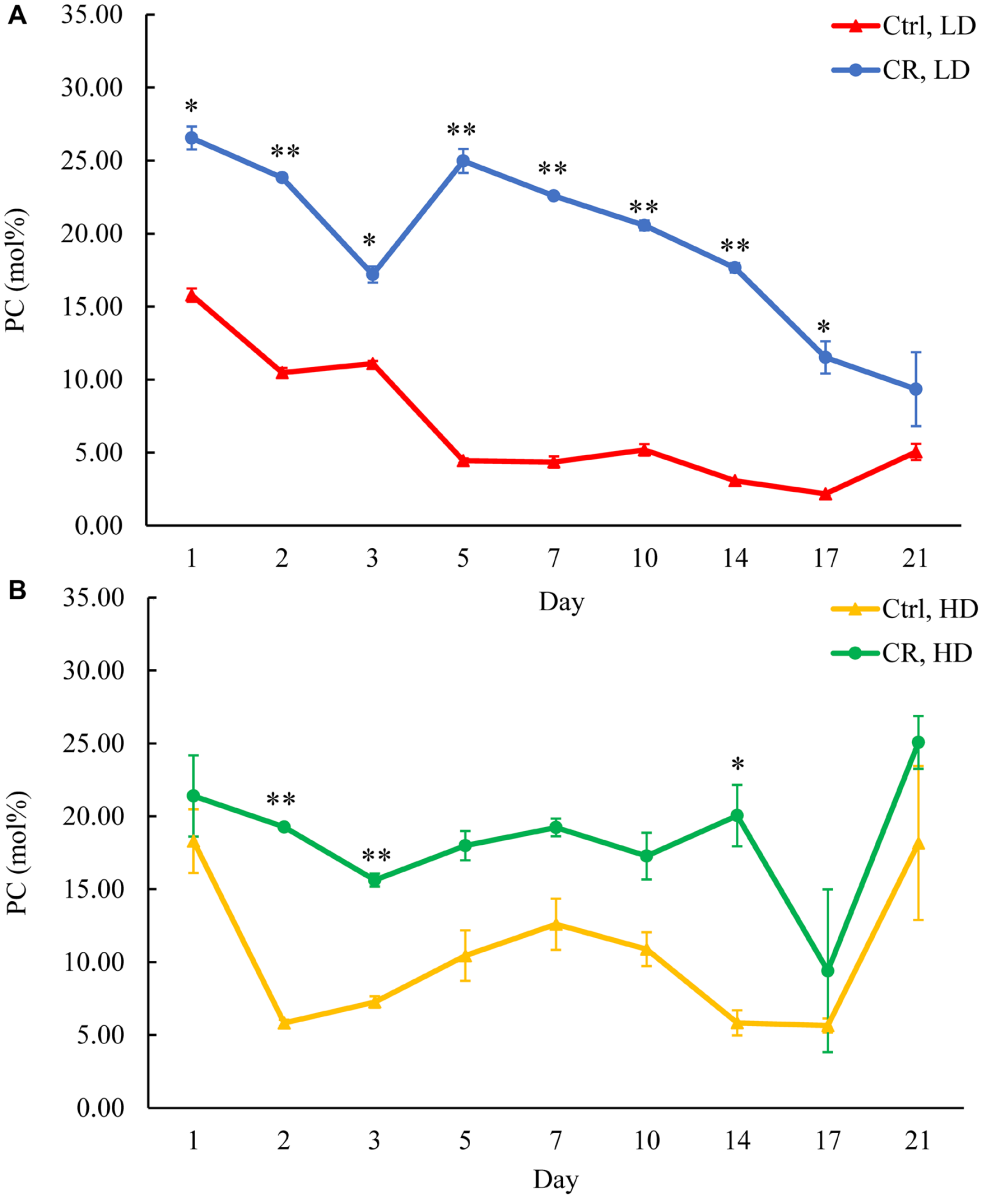
CR increases PC concentration in both HD and LD cells through the chronological lifespan. Samples of WT yeast cultured in YP medium initially containing 0.2% glucose (CR conditions) or 2% glucose (control non-CR conditions) were recovered on different days of culturing and subjected to centrifugation in Percoll density gradient to purify HD and LD cell populations. PC concentrations were measured by LC-MS/MS. PC concentrations in LD (**A**) and HD (**B**) cells are shown. Data are presented as means ± SD (*n* = 2; ^*^ *p* < 0.05; ^**^ *p* < 0.01). Abbreviation: Ctrl: control.

**Figure 12:**
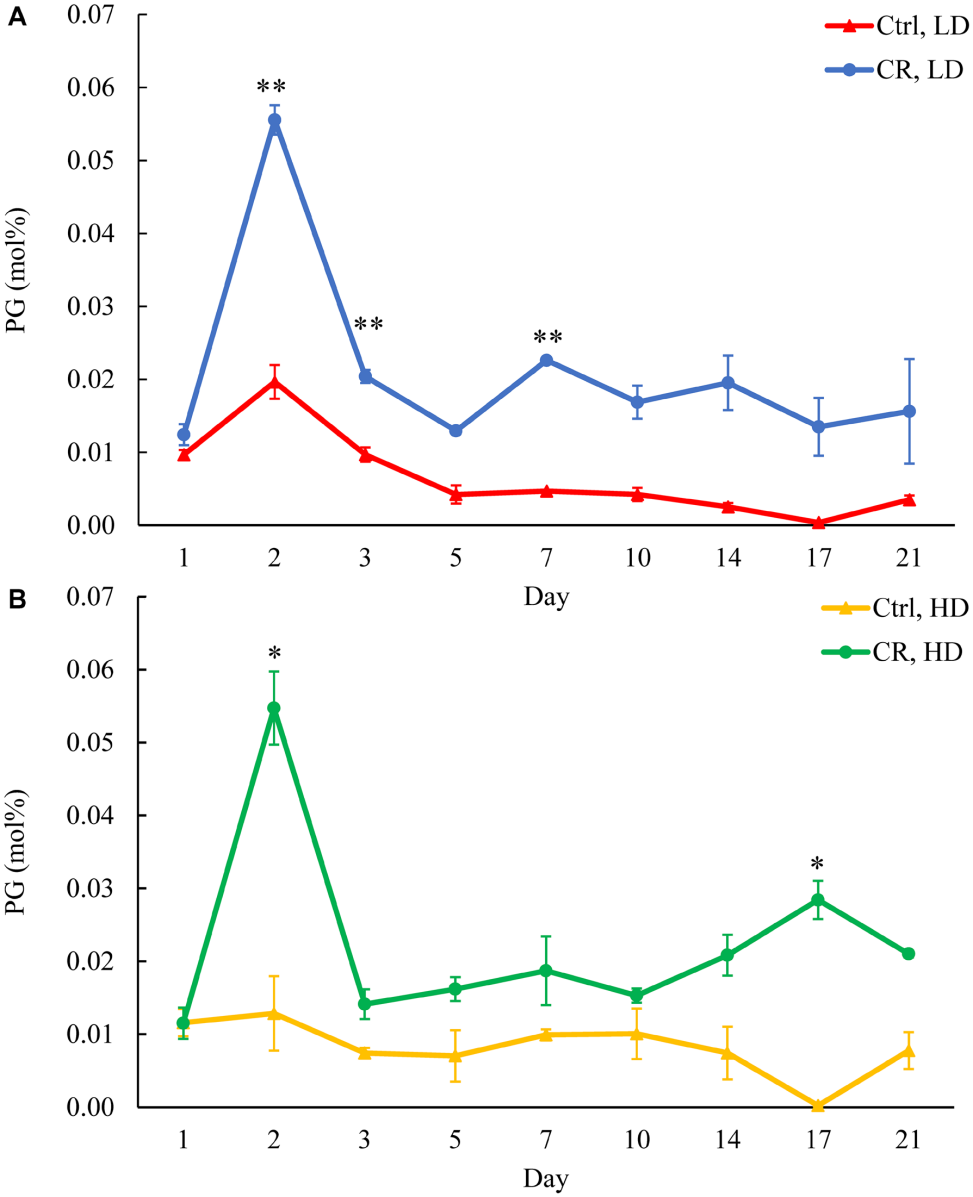
CR increases PG concentration in both HD and LD cells through the chronological lifespan. Samples of WT yeast cultured in YP medium initially containing 0.2% glucose (CR conditions) or 2% glucose (control non-CR conditions) were recovered on different days of culturing and subjected to centrifugation in Percoll density gradient to purify HD and LD cell populations. PG concentrations were measured by LC-MS/MS. PG concentrations in LD (**A**) and HD (**B**) cells are shown. Data are presented as means ± SD (*n* = 2; ^*^ *p* < 0.05; ^**^ *p* < 0.01).

**Figure 13:**
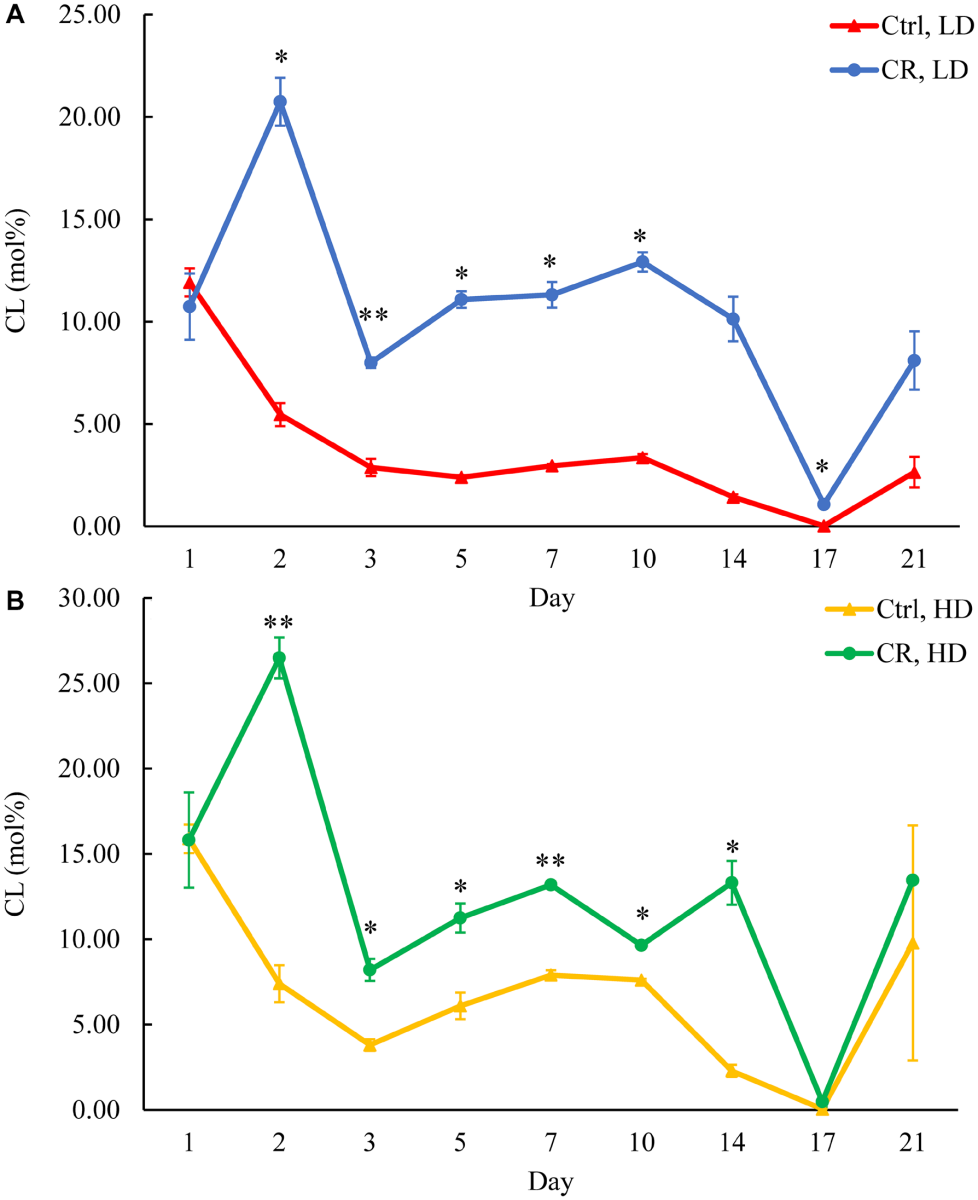
CR increases CL concentration in HD and especially in LD cells throughout the chronological lifespan. Samples of WT yeast cultured in YP medium initially containing 0.2% glucose (CR conditions) or 2% glucose (control non-CR conditions) were recovered on different days of culturing and subjected to centrifugation in Percoll density gradient to purify HD and LD cell populations. CL concentrations were measured by LC-MS/MS. CL concentrations in LD (**A**) and HD (**B**) cells are shown. Data are presented as means ± SD (*n* = 2; ^*^ *p* < 0.05; ^**^ *p* < 0.01).

We assessed the concentrations of phospholipids in differently aged HD and LD cells. These phospholipids included the so-called major forms of phospholipids [29, 30, 32] LPA, PA, PI, PS and PC synthesized in the ER (Figure 2) as well as PE, PG and CL formed in mitochondria (Figure 2). These phospholipids also included the so-called minor forms of phospholipids [29, 30, 32], lysophosphatidylinositol (LPI), lysophosphatidylserine (LPS), lysophosphatidylethanolamine (LPE), lysophosphatidylcholine (LPC) and lysophosphatidylglycerol (LPG). These minor forms of phospholipids are synthesized in the enzymatic reactions catalyzed by the broad specificity lysophospholipid acyltransferase Ale1 in the ER, LPC acyltransferase Taz1 in mitochondria, and LPI acyltransferase Psi1 in LDs and mitochondria [29, 30, 32]. We found that CR increases the concentrations of all these phospholipids in HD and LD cells through most of the chronological lifespan. The data for the effects of CR on LPA (Figure 6), PA (Figure 7), PI (Figure 8), PS (Figure 9), PE (Figure 10), PC (Figure 11), LPI (Supplementary Figure 13), LPS (Supplementary Figure 14), LPE (Supplementary Figure 15), LPC (Supplementary Figure 16), PG (Figure 12), CL (Figure 13) and LPG (Supplementary Figure 17) are shown in the major-set and Supplementary Figures. Of note, a relative rise in the concentrations of many of these phospholipids within short-lived LD populations of Q and NQ cells exceeded that in long-lived HD populations of Q and NQ cells.

Unlike CR, neither the *tor1Δ* mutation nor LCA elicited significant and long-lasting changes in all these phospholipids’ concentrations within HD and LD cells. The data on how *tor1Δ* and LCA affected LPA (Supplementary Figures 18 and 19), PA (Supplementary Figures 20 and 21), PI (Supplementary Figures 22 and 23), PS (Supplementary Figures 24 and 25), PE (Supplementary Figures 26 and 27), PC (Supplementary Figures 28 and 29), LPI (Supplementary Figures 30 and 31), LPS (Supplementary Figures 32 and 33), LPE (Supplementary Figures 34 and 35), LPC (Supplementary Figures 36 and 37), PG (Supplementary Figures 38 and 39), CL (Supplementary Figures 40 and 41) and LPG (Supplementary Figures 42 and 43) concentrations in differently aged HD and LD cells are presented in Supplementary Material.

## DISCUSSION

**Figure 14:**
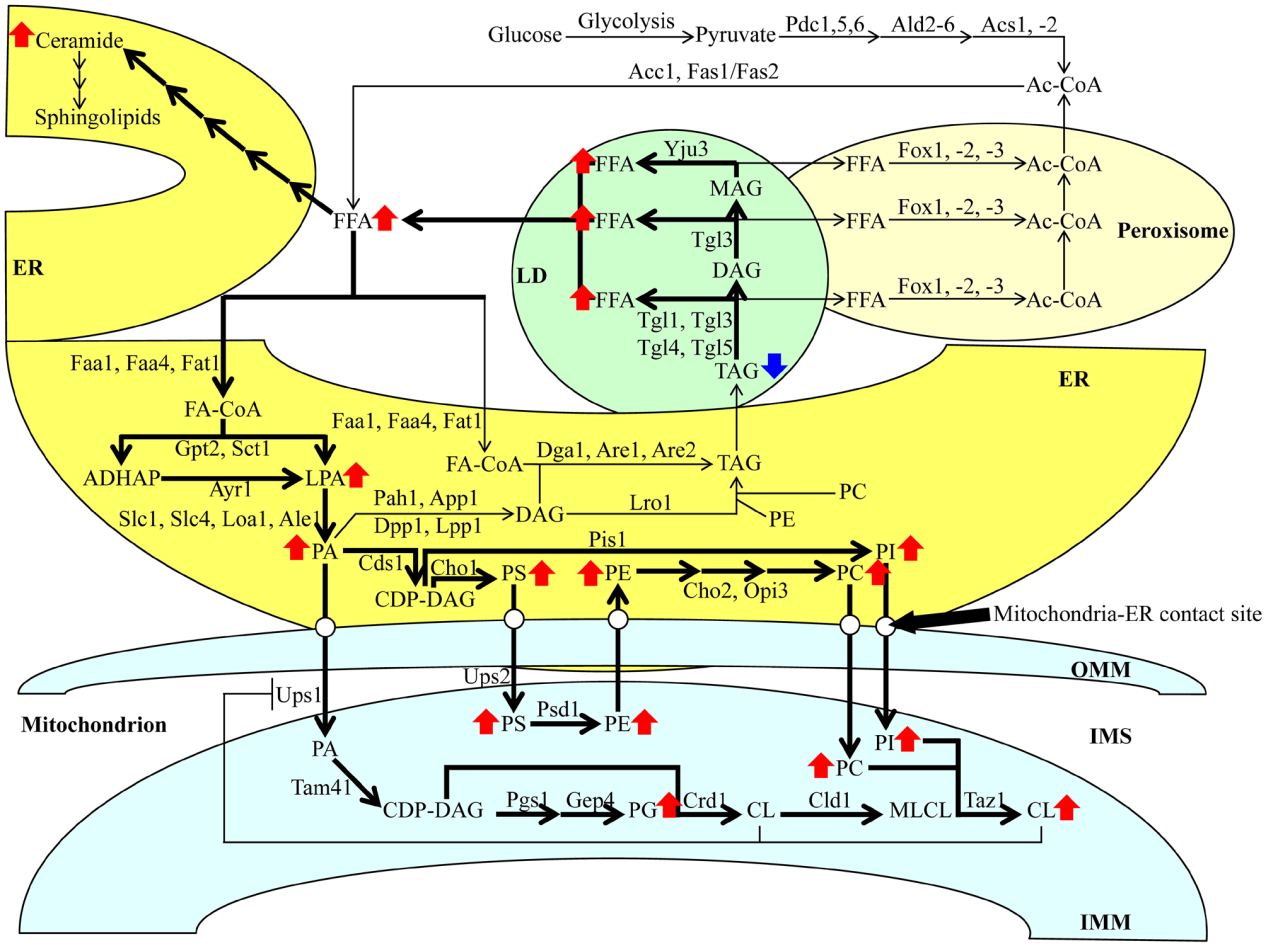
A model for a CR-dependent remodeling of lipid metabolism and transport in HD and LD cells. CR stimulates FFA formation because this low-calorie diet promotes the lipolysis of the neutral lipids TAG. CR also activates the incorporation of FFA into various phospholipid classes in the ER and mitochondria. Arrows next to the names of lipid classes indicate those whose concentrations are increased (red arrows) or decreased (blue arrows) in yeast cells cultured under CR conditions. The thickness of black arrows is proportional to the efficiency with which TAG is hydrolyzed, FFA is incorporated into phospholipids and phospholipids are transported from the ER to mitochondria via the mitochondria-ER contact sites. Abbreviations: All abbreviations are provided in the text and the legend for Figure 2.

We used Percoll density centrifugation to purify HD and LD cells from differently aged yeast cultures. The cultures were incubated in a nutrient-rich medium initially containing 0.2% glucose (CR conditions) or 2% glucose (non-CR conditions) as a sole carbon source. An HD cell population contains both Q and NQ cells. In the HD population, Q cells undergo a relatively slow conversion into NQ cells during chronological aging [14, 16, 17]. An LD cell population is also a mixture of Q and NQ cells. Q cells in the LD population are converted into NQ cells faster during chronological aging than in the HD population [14, 16, 17]. We used the LC-MS/MS-based quantitative lipidomics to assess the aging-associated changes in HD and LD cells’ lipidomes. Previous studies showed that CR, a robust geroprotective dietary intervention, decreases TAG concentration and increases CL concentration in HD and LD cells of budding yeast [14, 16, 17]. Here, we provided the first evidence that CR statistically significantly alters the concentrations of many different lipid classes through most of the chronological lifespan of HD and LD cells. To assess the aging-associated changes in each of these lipid classes, we calculated their concentrations in mol% of all lipids. Our data indicate that CR elicits a characteristic remodeling of HD and LD cell lipidomes. This CR-specific remodeling of the entire cellular lipidome is outlined below and schematically depicted in Figure 14.

CR simulates TAG lipolysis in LDs, likely by activating the neutral lipid hydrolases Tgl1, Tgl3, Tgl4, Tgl5 and Yju3 (Figure 14). The CR-driven stimulation of TAG lipolysis causes a significant rise in the intracellular concentrations of FFA in the cytosol (Figure 14). The flow of FFA under CR conditions is directed into the synthesis of CER and all phospholipid classes in the ER. These phospholipid classes include the so-called major forms of phospholipids, such as LPA, PA, PI, PS, PE and PC (Figure 14). These phospholipid classes also include the so-called minor forms of phospholipids, such as LPS and LPE. The concentrations of all these major and minor phospholipid forms within the ER of the HD and LD cells cultured under CR conditions exceed those within the ER of the HD and LD cells not limited in calorie supply (Figure 14). CR also promotes PA, PS, PC and PI transport from the ER to mitochondria through the mitochondria-ER contact sites (Figure 14). The activation of PS transport into mitochondria results in the CR-dependent excessive synthesis of PE from PS in these organelles (Figure 14). The activation of PA, PC and PI transport into mitochondria elicits excessive CR-dependent synthesis of PG and CL in these organelles (Figure 14). The concentrations of the major phospholipid forms (including PG, CL, PS, PE, PC and PI) and the minor ones (such as LPC and LPI) within mitochondria of the HD and LD cells cultured under CR conditions are higher than those within mitochondria of the HD and LD cells not limited in calorie supply (Figure 14).

We hypothesize that the remodeling of HD and LD cells lipidomes under CR conditions may contribute to the CR-dependent aging delay and longevity extension of these cells. We propose the following two mechanisms for such contribution. Most phospholipids whose concentrations rise under CR conditions are the ones that are synthesized and reside in the ER (Figure 14). The ER lipidome changes affect lipid and protein homeostasis within this organelle. These changes are known to activate the so-called unfolded protein response in the ER (UPR^ER^) of yeast and other organisms [39–41]. After being activated, the UPR^ER^ system allows the restoration of lipid and protein homeostasis in the ER. This regulatory system accelerates lipid synthesis, slows protein translation and stimulates a refolding of improperly folded proteins in the ER [41, 42]. The UPR^ER^ system is an essential contributor to aging delay and longevity extension in yeast and other organisms [42–44]. Therefore, we hypothesize that one mechanism for delaying the chronological aging of yeast synthesizing excessive amounts of phospholipids in the ER under CR conditions may involve activating the UPR^ER^ system. The other mechanism for slowing the chronological aging of yeast synthesizing excessive amounts of PG, CL, PS, PE, PC, LPC and LPI in mitochondria under CR conditions may improve certain aspects of mitochondrial functionality. These aspects include such pro-longevity processes as activated mitochondrial respiration, elevated mitochondrial membrane potential and altered mitochondrial ROS production observed in HD and LD sub-populations of Q and NQ cells [14]. Future studies are needed to test our hypotheses about the two mechanisms through which the observed remodeling of HD and LD cells lipidomes under CR conditions may contribute to the CR-dependent aging delay. These future studies may involve experiments on assessing the effects of mutations affecting the known protein components of the UPR^ER^ system, redundant enzymes catalyzing phospholipid formation in the ER (Figure 14) or enzymes catalyzing CL synthesis in the IMM of mitochondria on the extent of the CR-dependent chronological aging delay in yeast.

We noticed that a relative increase in the concentrations of PI, PS, PE, LPI and LPE within the short-lived LD sub-populations of Q and NQ cells is higher than that within the long-lived HD populations of Q and NQ cells. This observation suggests the existence of an optimal threshold of these phospholipid concentrations at which the maximal aging-delaying effect can be achieved. We hypothesize that the concentrations of PI, PS, PE, LPI and LPE in the long-lived HD populations of Q and NQ cells are within the predicted optimal threshold. In contrast, the higher concentrations (i.e., above the optimal threshold) of PI, PS, PE, LPI and LPE in the short-lived LD populations of Q and NQ cells may shorten their longevity because these concentrations are toxic for the cell. One way of testing this hypothesis in the future is to assess if the mutations affecting redundant enzymes that catalyze phospholipid synthesis in the ER (Figure 14) could slow the chronological aging of LD cells.

To attain our second objective, we compared the effects of CR (a dietary geroprotector), the *tor1Δ* mutation (a genetic geroprotector) and LCA (a pharmacological geroprotector) on the lipidomes of HD and LD cells recovered on different stages of the chronological aging process. Our comparative analysis revealed that CR creates a lipidomic pattern within the HD and LD cells that differs from the lipidomic design established by the *tor1Δ* mutation or LCA. Indeed, only CR (but not the *tor1Δ* mutation or LCA) decreased TAG concentration in HD and LD cells through the most chronological lifespan. Furthermore, only CR (but not the *tor1Δ* mutation or LCA) increased FFA concentration in HD and LD cells through the most chronological lifespan. Moreover, only CR (but not the *tor1Δ* mutation or LCA) increased CER concentration in HD and LD cells throughout the chronological lifespan. Besides, only CR (but not the tor1Δ mutation or LCA) raised the concentrations of all ER- and mitochondria-synthesized phospholipids in HD and LD cells through most of the chronological lifespan.

Of note, our recent studies provided evidence that CR creates a metabolic pattern of chronological aging delay that in budding yeast differs from the metabolic design established by the *tor1Δ* mutation and LCA [33]. These metabolites are soluble in aqueous solutions. Thus, it seems that different dietary, genetic and pharmacological geroprotectors differ in their ability to affect the water-insoluble lipidomes and water-soluble metabolomes of chronologically aging budding yeast.

## MATERIALS AND METHODS

### Yeast strains, media and growth conditions

The WT strain *Saccharomyces cerevisiae* BY4742 (*MATα his3Δ1 leu2Δ0 lys2Δ0 ura3Δ0*) from Thermo Scientific/Open Biosystems was grown in YP medium (1% yeast extract, 2% peptone, both from Fisher Scientific) initially containing the following: 1) 2% (w/v) glucose (Fisher Scientific) as a carbon source, 2) 0.2% (w/v) glucose as a carbon source or 3) 0.2% (w/v) glucose as a carbon source and 50 μM LCA. The *tor1Δ* single-gene-deletion mutant strain in the BY4742 genetic background from Thermo Scientific/Open Biosystems was grown in a YP medium initially containing 2% (w/v) glucose as a carbon source. Cells were cultured at 30^°^C with rotational shaking at 200 rpm in Erlenmeyer flasks at a “flask volume/medium volume” ratio of 5:1. Cell aliquots for separating high-density and low-density quiescent cells by centrifugation in Percoll density gradient were collected on days 1, 2, 3, 5, 7, 10, 14, 17 and 21 culturing.

### Separation of the HD and LD populations of Q cells by centrifugation in Percoll density gradient

2 ml of 1.5 M NaCl (Sigma) was placed into a 50-ml conical polypropylene centrifuge tube (Fisher Scientific), and 16 ml of the Percoll solution (Sigma) was added to this tube. The NaCl and Percoll solutions were then mixed by pipetting. 4 ml of the NaCl/Percoll mixture was put into each of the four polyallomer tubes for an MLS-50 rotor for an Optima MAX ultracentrifuge (all from Beckman Coulter, Inc.) to form four Percoll density gradients. The tubes were centrifuged at 25,000 × g (16,000 rpm) for 15 min at 4^°^C in an Optima MAX ultracentrifuge. A sample of yeast cells was taken from a culture at a particular time point (see the previous section). A sample fraction was diluted to determine the total number of cells per ml of culture using a hemacytometer (Fisher Scientific). For each Percoll density gradient, 1 × 10^9^ yeast cells were placed into a 15-ml conical polypropylene centrifuge tube (Fisher Scientific) and then pelleted by centrifugation at 5,000 rpm for 7 min at room temperature (RT) in an IEC Centra CL2 clinical centrifuge (Thermo Electron Corporation). Pelleted cells were resuspended in 500 μl of 50 mM Tris/HCl buffer (pH 7.5), overlaid onto the preformed Percoll gradient and centrifuged at 2,300 × g (5,000 rpm) for 30 min at 25^°^C in an Optima MAX ultracentrifuge. The upper and lower fractions of cells were collected with a pipette, Percoll was removed by washing cells twice with 50 mM Tris/HCl buffer (pH 7.5) and cells were resuspended in 50 mM Tris/HCl buffer (pH 7.5) for lipid extraction.

### Identification and quantitation of cellular lipids using LC-MS/MS

After measuring the cell titer with the help of a hemocytometer, a volume of the cell suspension in 50 mM Tris/HCl buffer (pH 7.5) that contains the total number of 5.0 × 10^7^ cells was transferred into a pre-cooled at 4^°^C 1.5-ml microcentrifuge Eppendorf tube. The cells were harvested by centrifugation at 16,000 × g for 1 min at 4°C, and the supernatant was discarded. 1.5 ml of ice-cold nano-pure water was added to the pellet, the cells were washed by centrifugation at 16,000 × g for 1 min at 4°C, and the supernatant was discarded. 1.5 ml of ice-cold ammonium bicarbonate (ABC) solution was added to the pellet, the cells were washed by centrifugation at 16,000 × g for 1 min at 4°C, and the supernatant was discarded. The cell pellet was stored at −80^°^C before lipid extraction. After being held at −80°C, the cell pellet was thawed on ice and then resuspended in 200 μl of ice-cold nano-pure water. The cell suspension was transferred to a 15-ml high-strength glass screw-top centrifuge tube with a polytetrafluoroethylene-lined cap. The following was added to this tube: 1) 25 μl of the mixture of internal lipid standards prepared in chloroform/methanol (2:1) mixture, 2) 100 μl of 425–600 μM acid-washed glass beads, and 3) 600 μl of chloroform/methanol (17:1) mixture. The tube was vortexed at high speed for 5 min at RT to disrupt the cells. The tube was then vortexed at low speed for 1 h at RT to facilitate lipids extraction. The sample was incubated for 30 min on ice to promote protein precipitation and separate the aqueous and organic phases from each other. The tube was centrifuged in a clinical centrifuge at 3,000 × g for 5 min at RT to facilitate the separation of the upper aqueous phase and the lower organic phase, which contained all lipid classes. A borosilicate glass pipette was used to transfer the lower organic phase (~400 μl) to another 15-ml high-strength glass screw-top centrifuge tube with a polytetrafluoroethylene lined cap. The lower organic phase was kept under the flow of nitrogen gas. 300 μl of chloroform-methanol (2:1) mixture was added to the remaining upper aqueous phase to allow the extraction of sphingolipids, LPA, lysophosphatidylglycerol (LPG), lysophosphatidylinositol (LPI), lysophosphatidylserine (LPS), PA, PG, PI and PS. The tube was vortexed vigorously for 5 min at RT and then centrifuged in a clinical centrifuge at 3,000 × g for 5 min at RT. A borosilicate glass pipette was used to transfer the lower organic phase (~ 200 μl) formed after centrifugation to the organic phase collected at the previous step. The solvent in the combined organic phases was evaporated under the flow of nitrogen gas. The tube containing the lipid film was closed under nitrogen gas flow and then stored at −80°C.

500 μl of acetonitrile (ACN)/2-propanol/nano-pure water (65:35:5) mixture was added to a tube containing the lipid film stored at −80°C, and the tube was vortexed 3 times for 10 s at RT. The tube’s content was subjected to ultrasonic sonication for 15 min, and the tube was vortexed again 3 times for 10 s at RT. 100 μl of a sample was taken from the tube and added to a glass vial with an insert used for a wellplate. An LC system was used to separate different lipid species on a reverse-phase C18 column CSH coupled to a pre-column system (Waters). During lipid separation, the column was maintained at 55°C and a flow rate of 0.3 ml/min, and the sample was kept in the wellplate at RT. The mobile phases that consisted of mixture A (ACN/water (60:40 (v/v))) and mixture B (isopropanol/ACN (90:10 (v/v))) were used for the chromatographic separation of lipids. For a positive mode of detecting parent ions created using the electrospray ionization (ESI) ion source, the ESI (+) mode, the mobile phases A and B contained ammonium formate at the final concentration of 10 mM. For a negative mode of parent ions detection, the ESI (−) mode, the mobile phases A and B contained ammonium acetate at the final concentration of 10 mM. A sample volume of 10 μl was used for the injection in both the ESI (+) and ESI (−) modes. Different lipid species were separated by LC using the following LC gradient: 0–1 min 10% (phase B); 1–4 min 60% (phase B); 4–10 min 68% (phase B); 10–21 min 97% (phase B); 21–24 min 97% (phase B); 24–33 min 10% (phase B). Extraction blanks were run as the first sample, between every four samples, and as the last sample. The background was subtracted to normalize the data.

A mass spectrometer equipped with a HESI (heated electrospray ionization) ion source was used to analyze lipids separated by LC. The settings used for such analysis are provided in Supplementary Table 1 [37]. The Fourier transform analyzer was used to detect parent ions (MS1) at a resolution of 60,000 and within the mass range of 150–2,000 Da. The settings shown in Supplementary Table 2 were used to detect secondary ions (MS2) [37].

The Lipid Search software (V4.1; Fisher Scientific) was used to identify and quantify different lipids from raw LC-MS/MS files. This software uses the largest lipid database, containing more than 1.5 million MS1 and MS2 ions. The software also uses MS1 peaks for lipid quantitation and MS2 for lipid identification. LC-MS raw files containing full-scan MS1 data and data-dependent MS2 data were searched for FFA, CL, PA, phytoceramide (PHC), phytosphingosine (PHS), PC, PE, PG, PI, PS, LPA, lysophosphatidylcholine (LPC), LPG, lysophosphatidylethanolamine (LPE), LPI, LPS, and TAG lipid classes. The m/z tolerance values of 5 ppm and 10 ppm were used for MS1 and MS2 ions. Other search parameters are provided in Supplementary Table 3 [37].

### Miscellaneous procedures

Statistical analysis was performed using Microsoft Excel’s Analysis ToolPack-VBA.

## SUPPLEMENTARY MATERIALS



## Abbreviations

ABC: ammonium bicarbonate
Ac-CoA: acetyl-CoA
ACN: acetonitrile
ADHAP: acyl-dihydroxyacetone phosphate
CDP: cytidine diphosphate
CL: cardiolipins
CER: ceramides
CR: caloric restriction
DAG: diacylglycerols
DHAP: acyl-dihydroxyacetone phosphate
ER: endoplasmic reticulum
ESI: electrospray ionization
FA-CoA: fatty acyl-CoA esters
FFA: free fatty acid
G3P: glycerol-3-phosphate
HESI: heated electrospray ionization
IMM: inner mitochondrial membrane
IMS: intermembrane space
LCA: lithocholic acid
LC-MS/MS: liquid chromatography coupled with tandem mass spectrometry
LDs: lipid droplets
LPA: lysophosphatidic acid
LPC: lysophosphatidylcholine
LPE: lysophosphatidylethanolamine
LPG: lysophosphatidylglycerol
LPI: lysophosphatidylinositol
LPS: lysophosphatidylserine
MAG: monoacylglycerols
MLCL: monolysocardiolipin
MS1: parent ions
MS2: secondary ions
NQ: non-quiescent cells
OMM: outer mitochondrial membrane
PA: phosphatidic acid
PC: phosphatidylcholine
PE: phosphatidylethanolamine
PG: phosphatidylglycerol
PI: phosphatidylinositol
PHC: phytoceramide
PHS: phytosphingosine
PS: phosphatidylserine
Q: quiescent cells
RCD: regulated cell death
ROS: reactive oxygen species
RT: room temperature
SPH: sphingolipids
ST: stationary growth phase
TAG: triacylglycerols
WT: wild type
ΔΨm: the electrochemical potential across the inner mitochondrial membrane
