# Supplementary material for: Caloric restriction causes a distinct reorganization of the lipidome in quiescent and non-quiescent cells of budding yeast
Source: Oncotarget. 2021 Nov 23;12(24):2351–74. doi: 10.18632/oncotarget.28133 (PMC8629408; doi:10.18632/oncotarget.28133)
Supplement: Supplementary file 1 [file oncotarget-12-2351-s001.pdf]

Caloric restriction causes a distinct reorganization of the lipidome in quiescent and non-quiescent cells of budding yeast

SUPPLEMENTARY MATERIALS

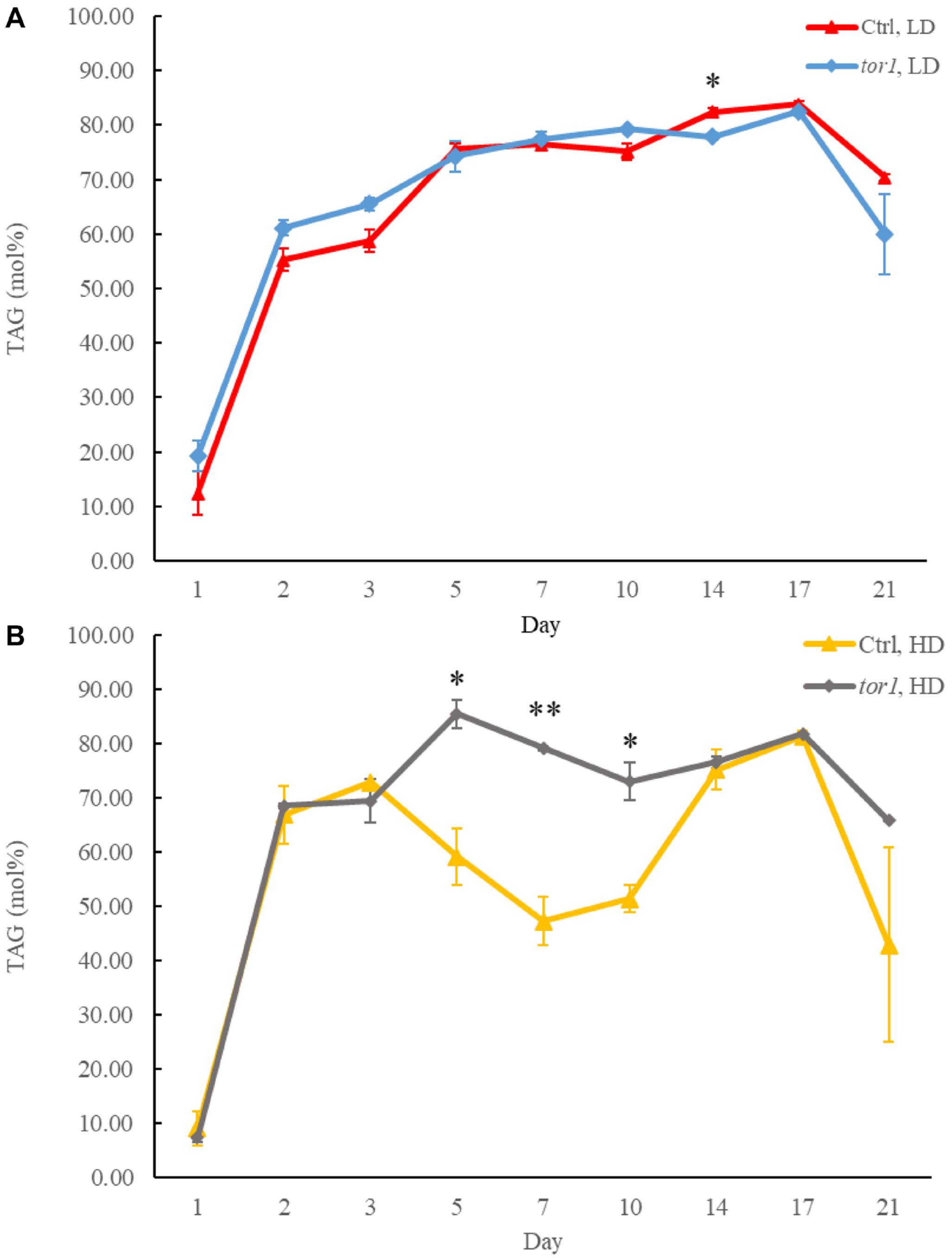

Supplementary Figure 1: The *tor1Δ* mutation increases TAG concentration in HD cells between days 5 and 10 of cell culturing. Samples of WT (control) and *tor1Δ* yeast cultured in YP medium initially containing 2% glucose (non-CR conditions) were recovered on different days of culturing and subjected to centrifugation in Percoll density gradient to purify HD and LD cell populations. TAG concentrations were measured by LC-MS/MS. TAG concentrations in LD (**A**) and HD (**B**) cells are shown. Data are presented as means ± SD ($n = 2$; $^{*}p < 0.05$; $^{**}p < 0.01$). Abbreviation: Ctrl: control.

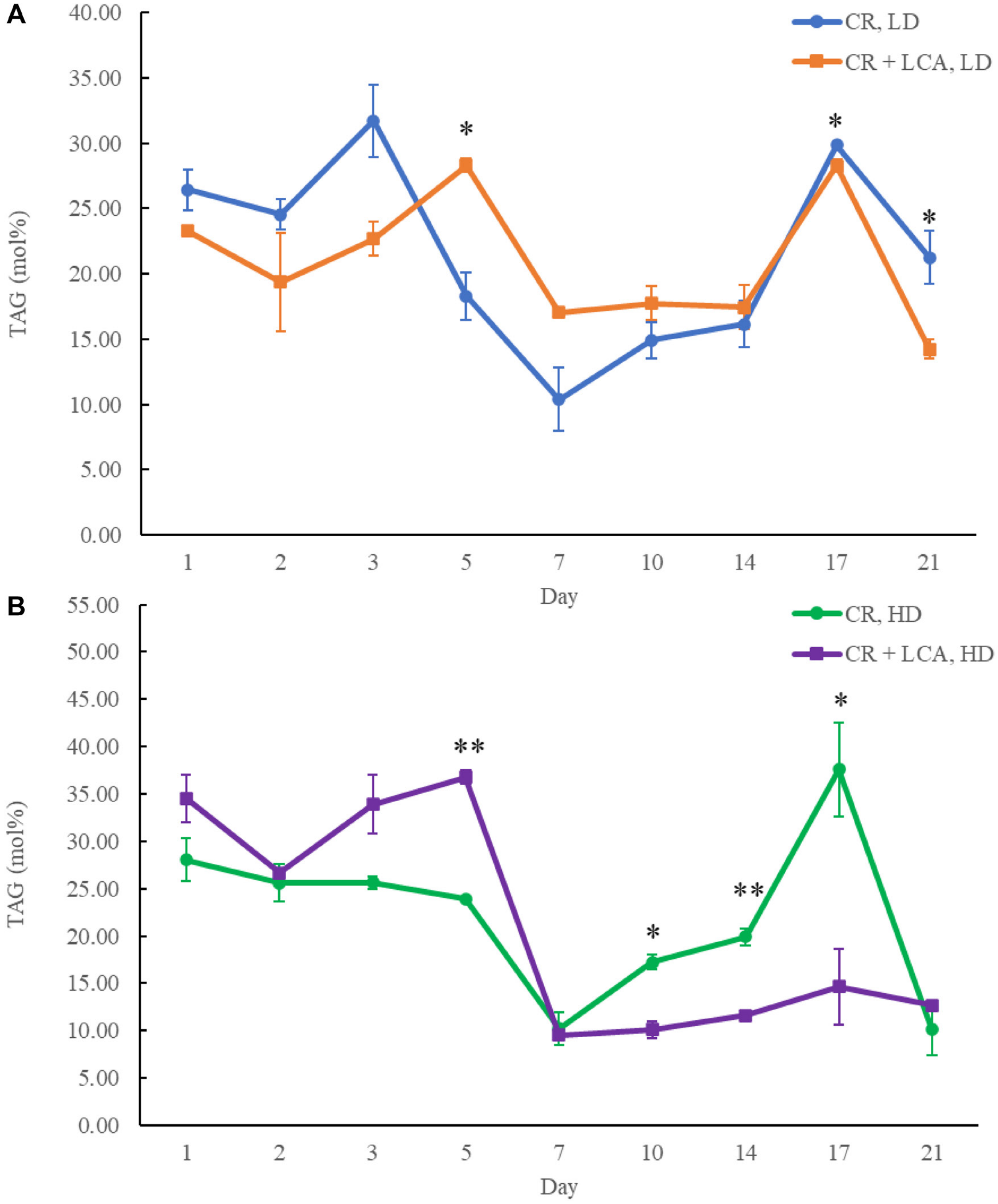

Supplementary Figure 2: LCA decreases TAG concentration in HD cells between days 10 and 17 of cell culturing. Samples of WT yeast cultured in YP medium initially containing 0.2% glucose (CR conditions) with 50 µM LCA or without it (control) were recovered on different days of culturing and subjected to centrifugation in Percoll density gradient to purify HD and LD cell populations. TAG concentrations were measured by LC-MS/MS. TAG concentrations in LD (**A**) and HD (**B**) cells are shown. Data are presented as means ± SD ($n = 2$; $^{*}p < 0.05$; $^{**}p < 0.01$).

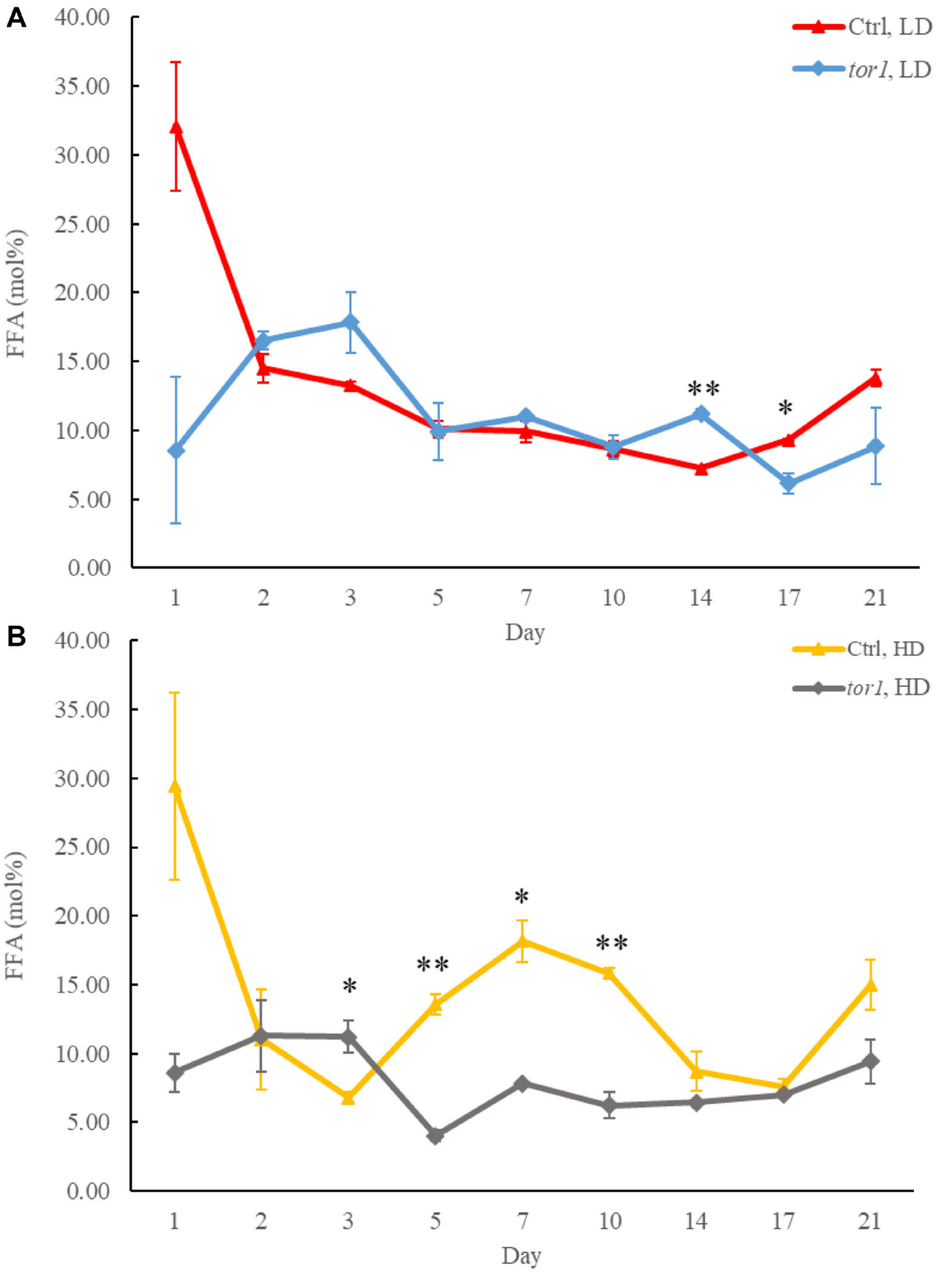

Supplementary Figure 3: The *tor1Δ* mutation decreases FFA concentration in HD cells between days 5 and 10 of cell culturing. Samples of WT (control) and *tor1Δ* yeast cultured in YP medium initially containing 2% glucose (non-CR conditions) were recovered on different days of culturing and subjected to centrifugation in Percoll density gradient to purify HD and LD cell populations. FFA concentrations were measured by LC-MS/MS. FFA concentrations in LD (**A**) and HD (**B**) cells are shown. Data are presented as means ± SD ($n = 2$; $^{*}p < 0.05$; $^{**}p < 0.01$). Abbreviation: Ctrl: control.

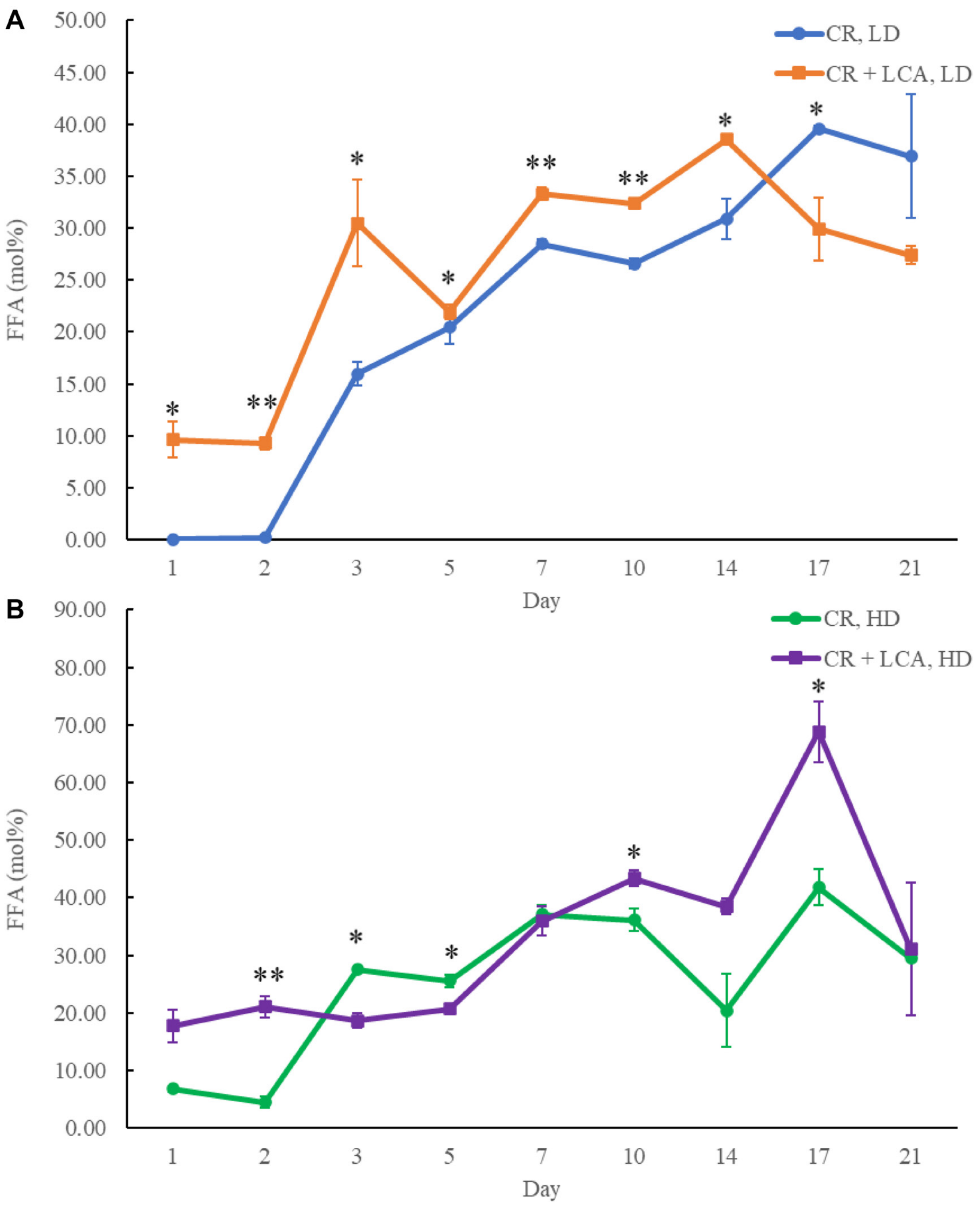

Supplementary Figure 4: LCA increases FFA concentration in LD cells and alters FFA concentration in HD cells through most of the chronological lifespan. Samples of WT yeast cultured in YP medium initially containing 0.2% glucose (CR conditions) with 50 µM LCA or without it (control) were recovered on different days of culturing and subjected to centrifugation in Percoll density gradient to purify HD and LD cell populations. FFA concentrations were measured by LC-MS/MS. FFA concentrations in LD (**A**) and HD (**B**) cells are shown. Data are presented as means ± SD ($n = 2$; $^{*}p < 0.05$; $^{**}p < 0.01$).

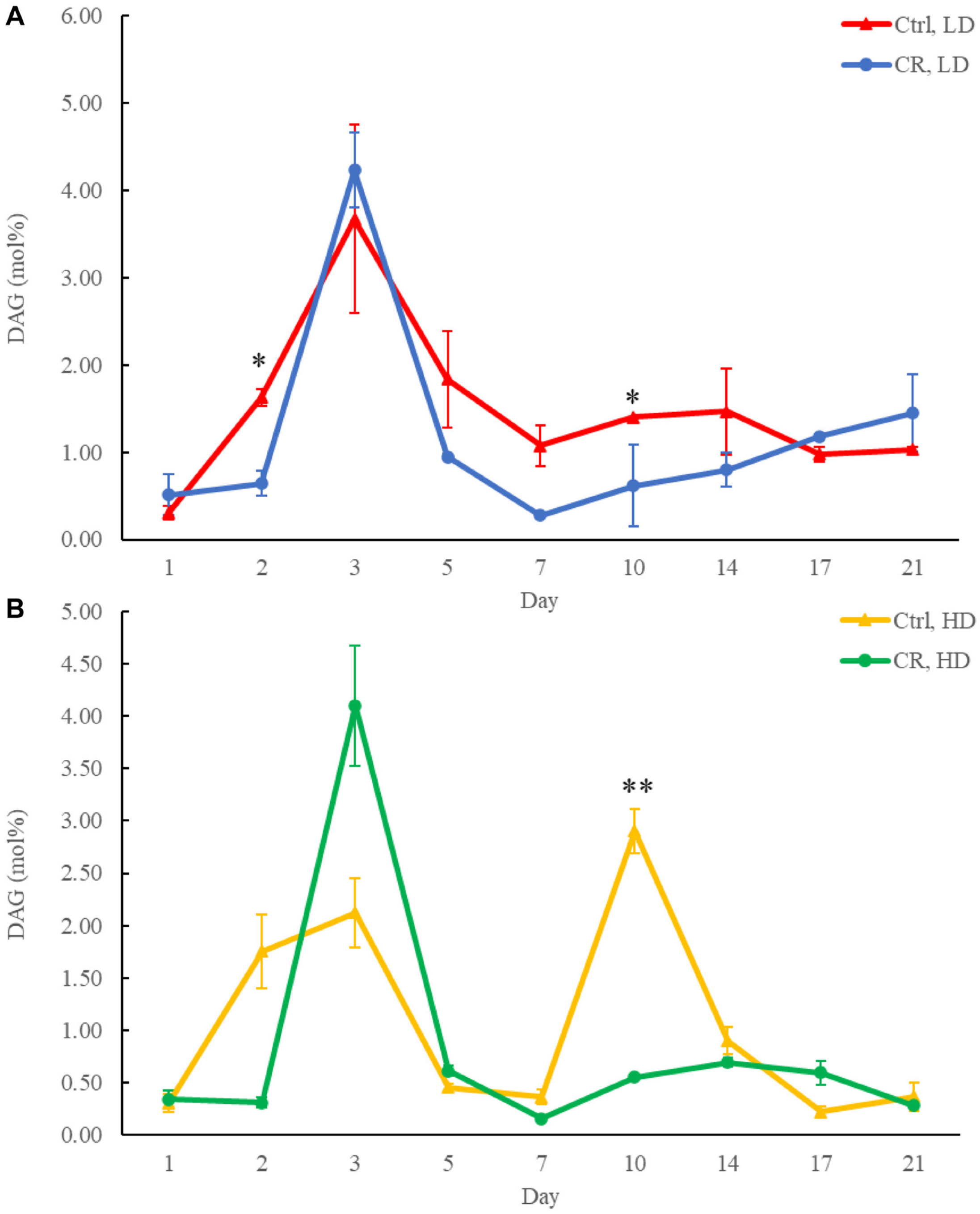

Supplementary Figure 5: CR does not have a long-lasting effect on DAG concentration in HD and LD cells through the chronological lifespan. Samples of WT yeast cultured in YP medium initially containing 0.2% glucose (CR conditions) or 2% glucose (control non-CR conditions) were recovered on different days of culturing and subjected to centrifugation in Percoll density gradient to purify HD and LD cell populations. DAG concentrations were measured by LC-MS/MS. DAG concentrations in LD (**A**) and HD (**B**) cells are shown. Data are presented as means ± SD ($n = 2$; $^{*}p < 0.05$; $^{**}p < 0.01$). Abbreviation: Ctrl: control.

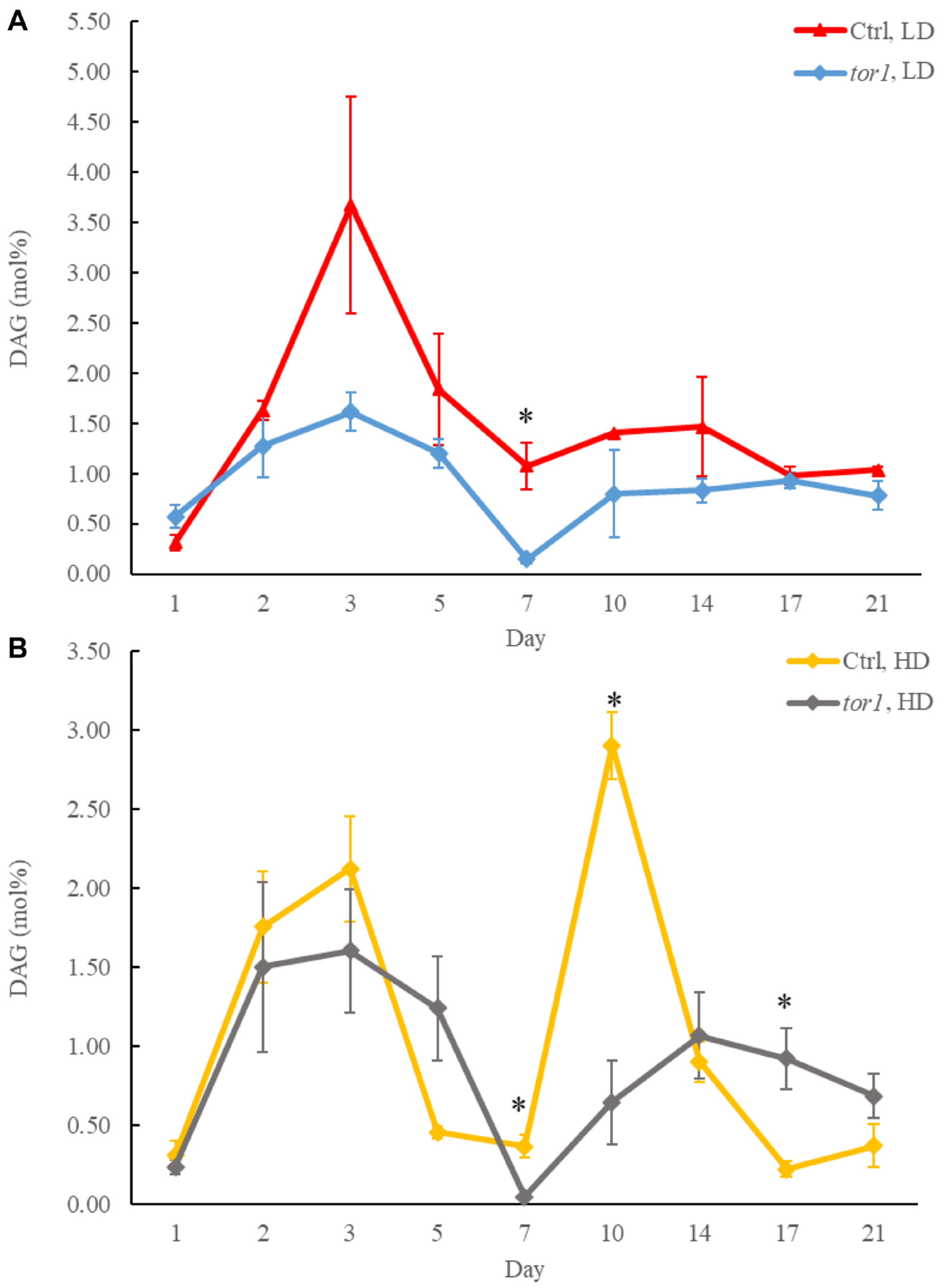

Supplementary Figure 6: The *tor1Δ* mutation does not have a long-lasting effect on DAG concentration in HD and LD cells through the chronological lifespan. Samples of WT (control) and *tor1Δ* yeast cultured in YP medium initially containing 2% glucose (non-CR conditions) were recovered on different days of culturing and subjected to centrifugation in Percoll density gradient to purify HD and LD cell populations. DAG concentrations were measured by LC-MS/MS. DAG concentrations in LD (**A**) and HD (**B**) cells are shown. Data are presented as means ± SD ($n = 2$; $^{*}p < 0.05$; $^{**}p < 0.01$). Abbreviation: Ctrl: control.

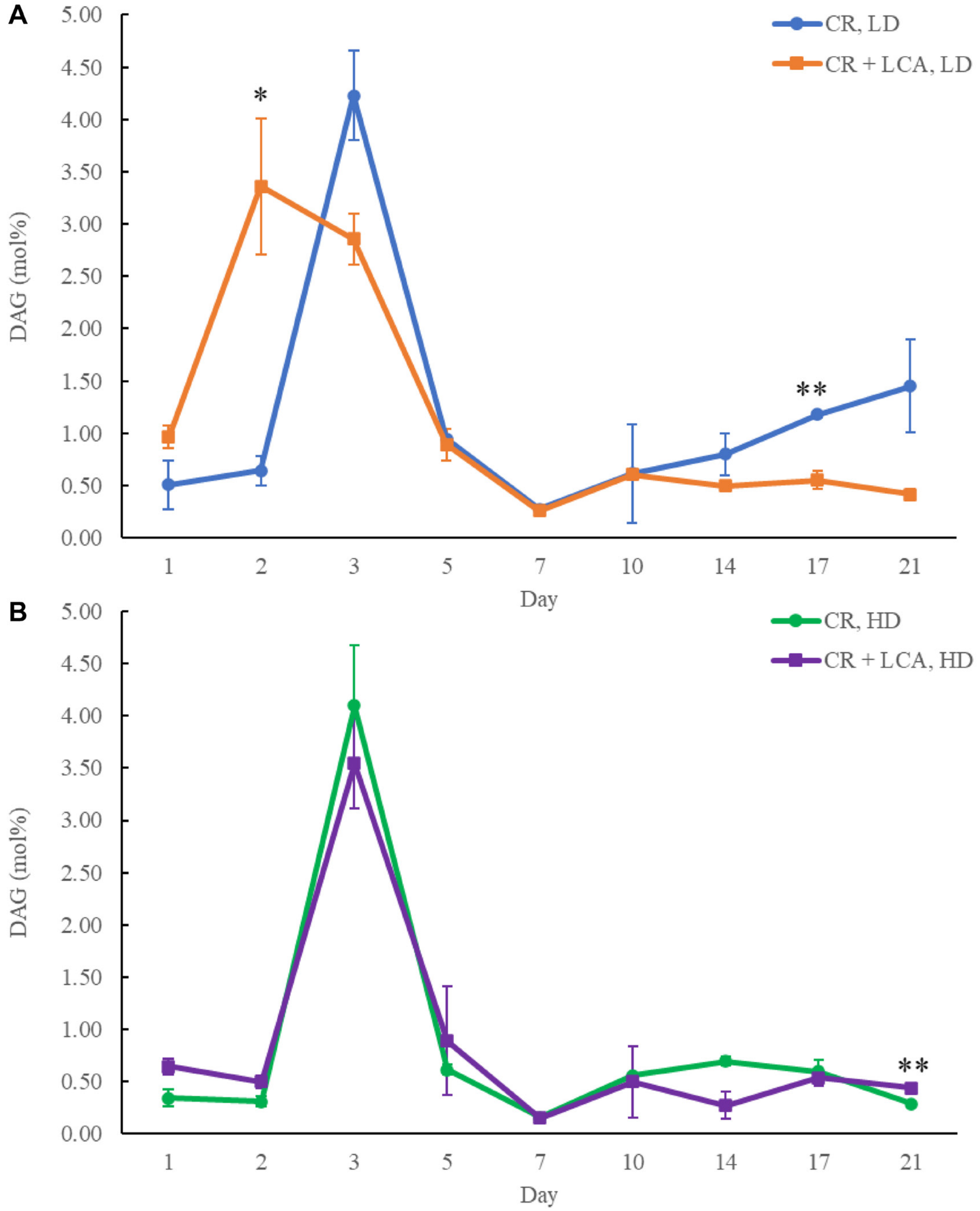

Supplementary Figure 7: LCA does not have a long-lasting effect on DAG concentration in HD and LD cells through the chronological lifespan. Samples of WT yeast cultured in YP medium initially containing 0.2% glucose (CR conditions) with 50 µM LCA or without it (control) were recovered on different days of culturing and subjected to centrifugation in Percoll density gradient to purify HD and LD cell populations. DAG concentrations were measured by LC-MS/MS. DAG concentrations in LD (**A**) and HD (**B**) cells are shown. Data are presented as means ± SD ($n = 2$; $^{*}p < 0.05$; $^{**}p < 0.01$).

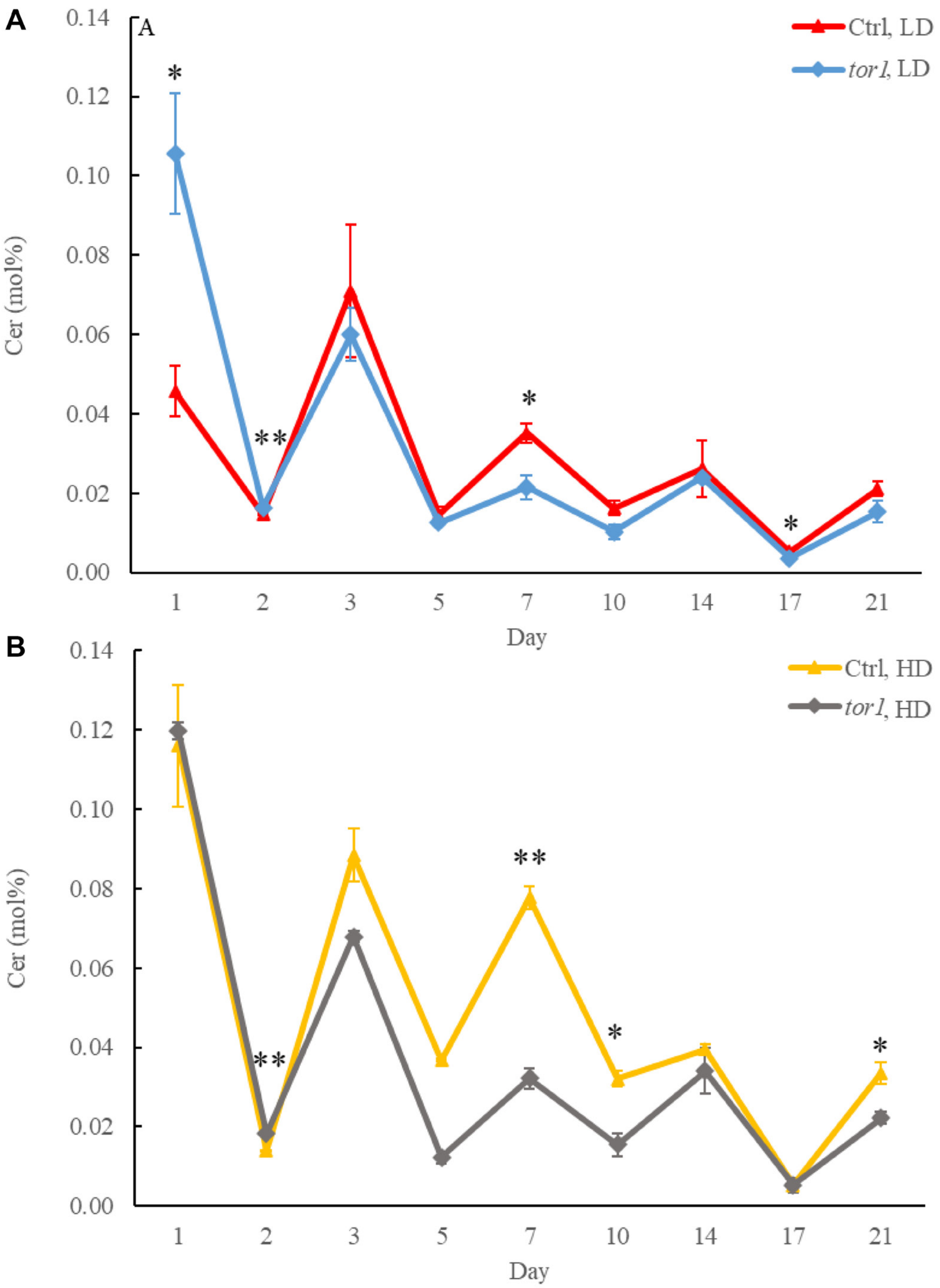

Supplementary Figure 8: The *tor1Δ* mutation decreases CER concentration in HD cells through most of the chronological lifespan. Samples of WT (control) and *tor1Δ* yeast cultured in YP medium initially containing 2% glucose (non-CR conditions) were recovered on different days of culturing and subjected to centrifugation in Percoll density gradient to purify HD and LD cell populations. CER concentrations were measured by LC-MS/MS. CER concentrations in LD (**A**) and HD (**B**) cells are shown. Data are presented as means ± SD ($n = 2$; $^{*}p < 0.05$; $^{**}p < 0.01$). Abbreviation: Ctrl: control.

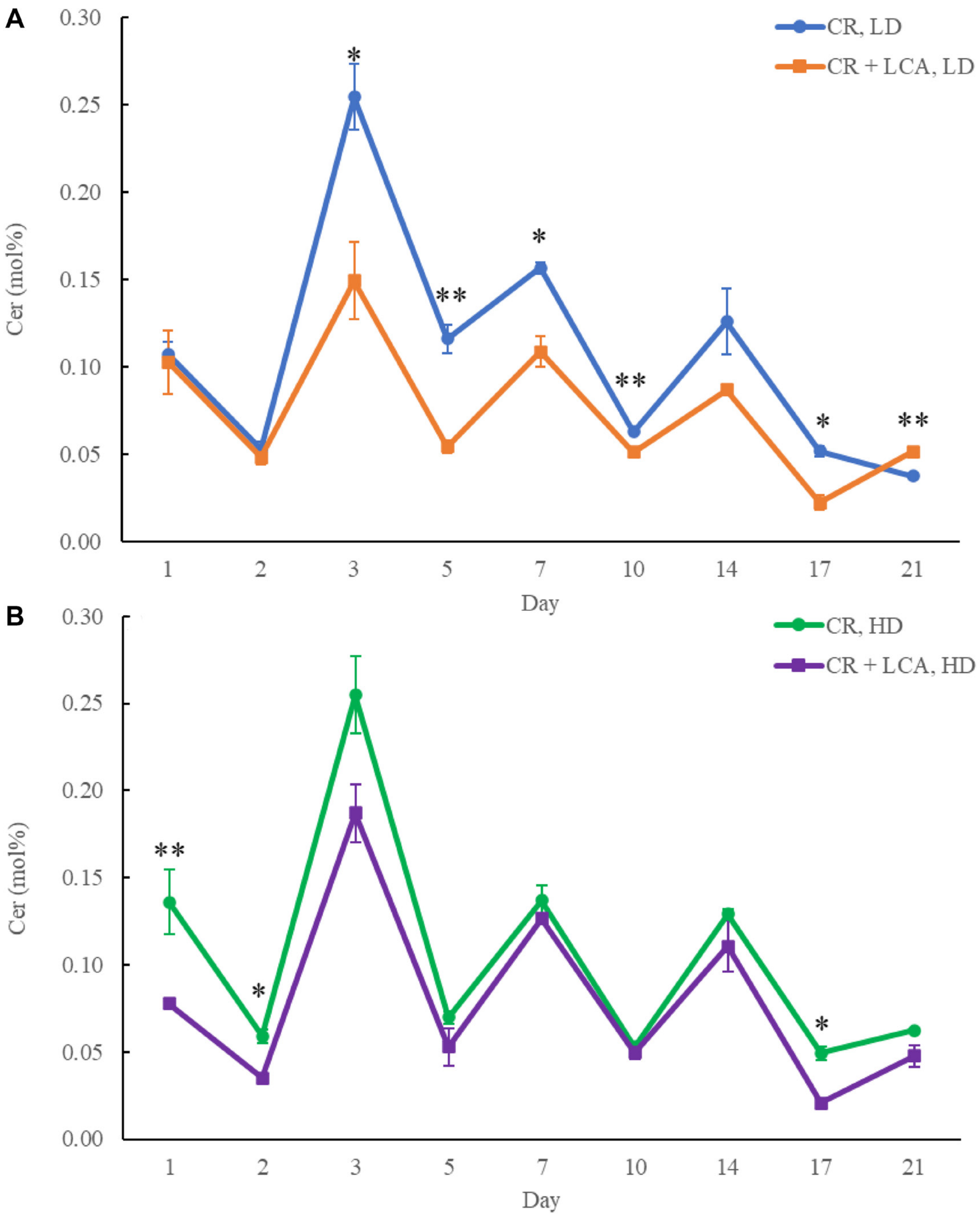

Supplementary Figure 9: LCA decreases CER concentration in LD cells through most of the chronological lifespan. Samples of WT yeast cultured in YP medium initially containing 0.2% glucose (CR conditions) with 50 µM LCA or without it (control) were recovered on different days of culturing and subjected to centrifugation in Percoll density gradient to purify HD and LD cell populations. CER concentrations were measured by LC-MS/MS. CER concentrations in LD (**A**) and HD (**B**) cells are shown. Data are presented as means ± SD ($n = 2$; $^{*}p < 0.05$; $^{**}p < 0.01$).

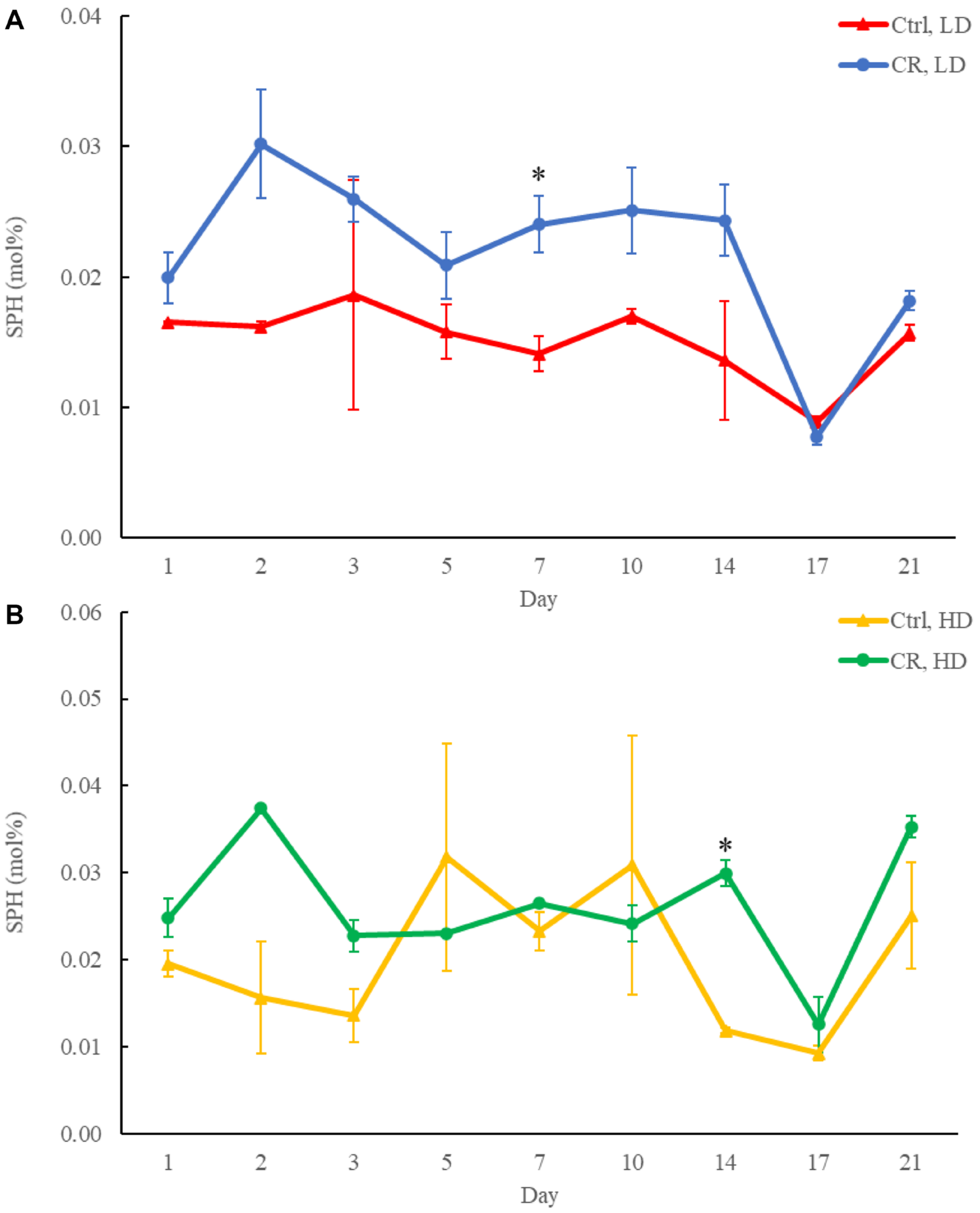

Supplementary Figure 10: CR does not significantly alter SPH concentrations in HD and LD cells through the chronological lifespan. Samples of WT yeast cultured in YP medium initially containing 0.2% glucose (CR conditions) or 2% glucose (control non-CR conditions) were recovered on different days of culturing and subjected to centrifugation in Percoll density gradient to purify HD and LD cell populations. SPH concentrations were measured by LC-MS/MS. SPH concentrations in LD (**A**) and HD (**B**) cells are shown. Data are presented as means ± SD ($n = 2$; $^{*}p < 0.05$). Abbreviation: Ctrl: control.

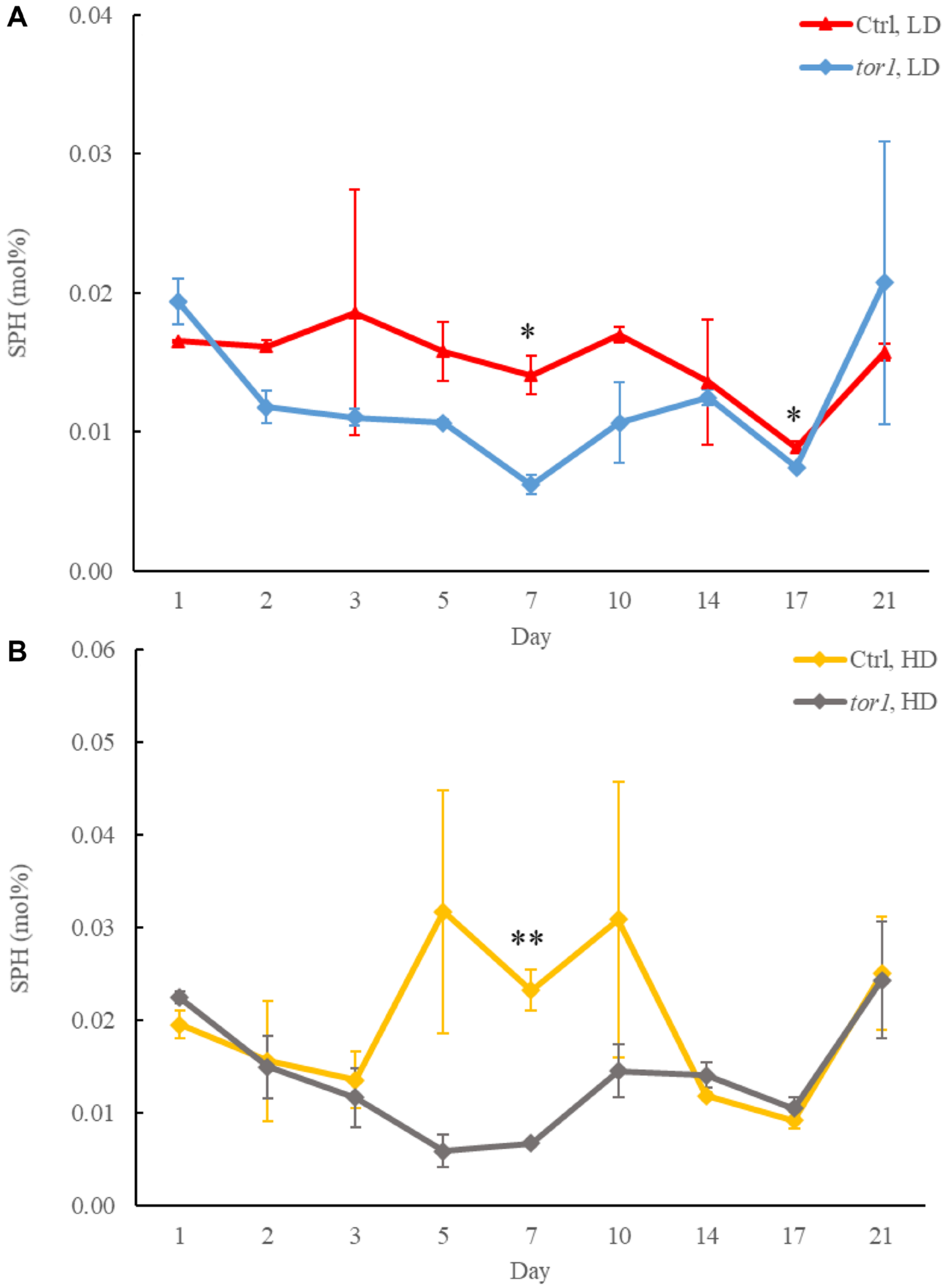

Supplementary Figure 11: The *tor1Δ* mutation does not cause a significant long-lasting effect on SPH concentrations in HD and LD cells. Samples of WT (control) and *tor1Δ* yeast cultured in YP medium initially containing 2% glucose (non-CR conditions) were recovered on different days of culturing and subjected to centrifugation in Percoll density gradient to purify HD and LD cell populations. SPH concentrations were measured by LC-MS/MS. SPH concentrations in LD (**A**) and HD (**B**) cells are shown. Data are presented as means ± SD ($n = 2$; $^{*}p < 0.05$; $^{**}p < 0.01$). Abbreviation: Ctrl: control.

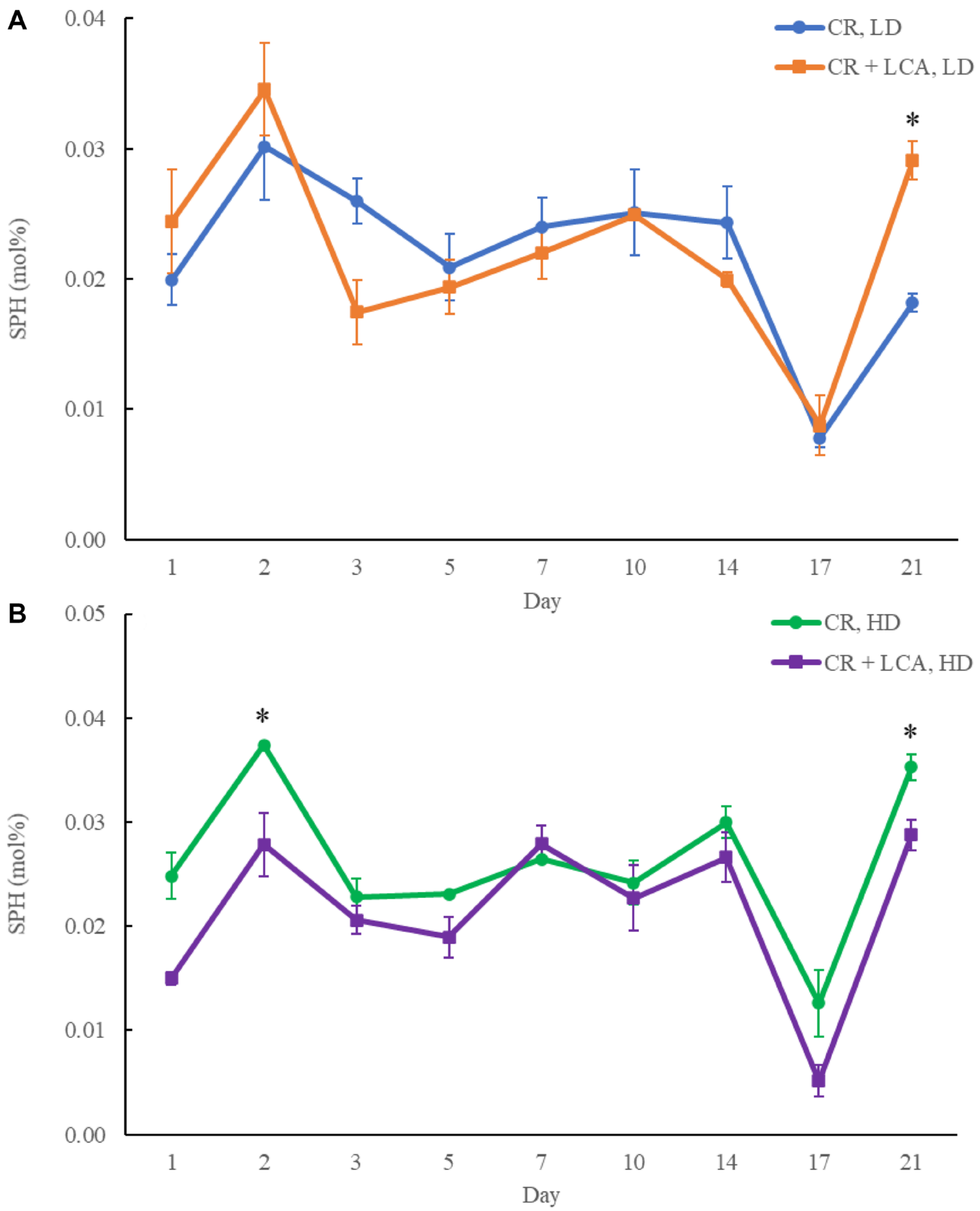

Supplementary Figure 12: LCA does not elicit a significant continuing effect on SPH concentrations in HD and LD cells. Samples of WT yeast cultured in YP medium initially containing 0.2% glucose (CR conditions) with 50 µM LCA or without it (control) were recovered on different days of culturing and subjected to centrifugation in Percoll density gradient to purify HD and LD cell populations. SPH concentrations were measured by LC-MS/MS. SPH concentrations in LD (**A**) and HD (**B**) cells are shown. Data are presented as means ± SD ($n = 2$; $^{*}p < 0.05$).

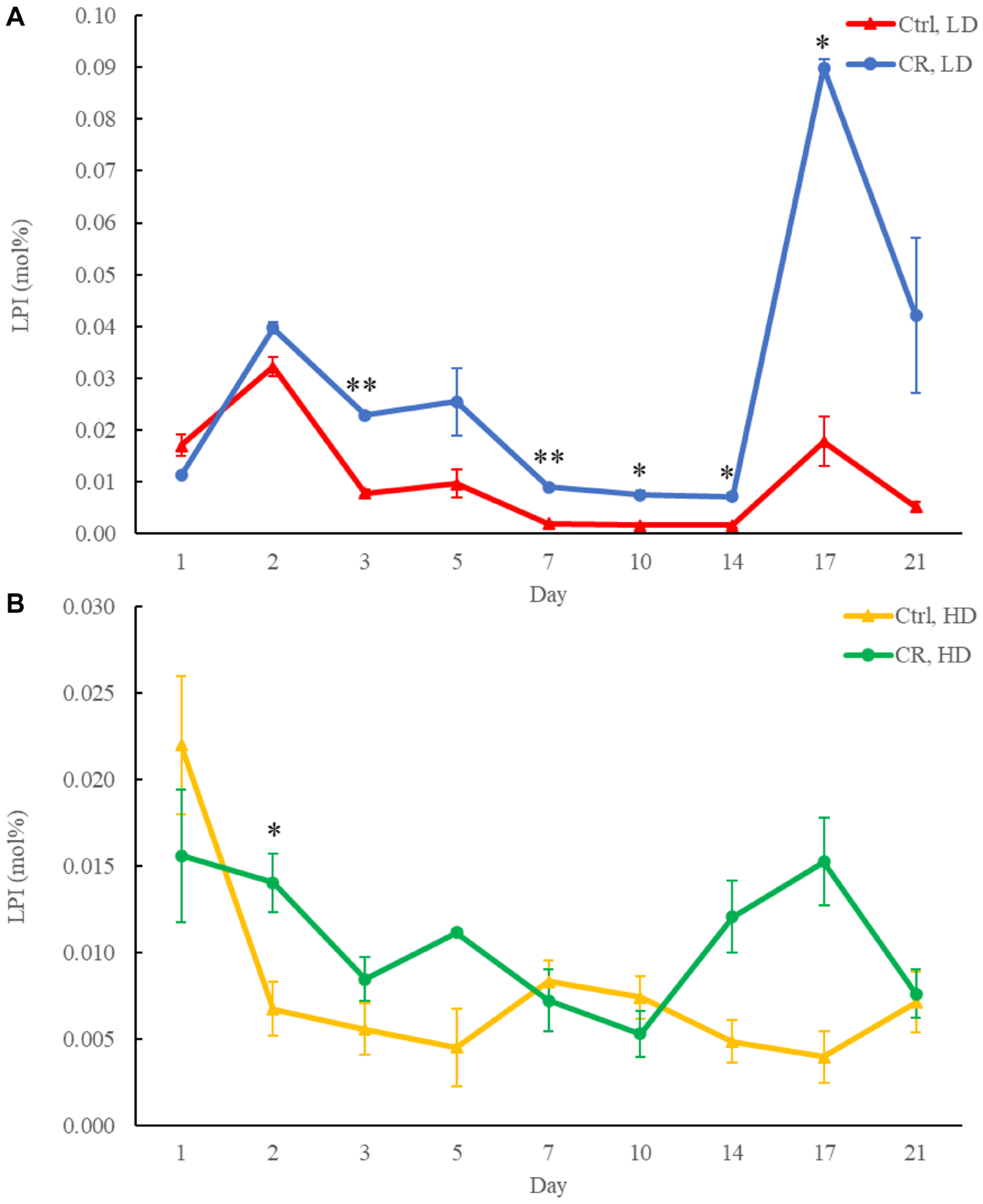

Supplementary Figure 13: CR increases LPI concentration in LD cells throughout the chronological lifespan. Samples of WT yeast cultured in YP medium initially containing 0.2% glucose (CR conditions) or 2% glucose (control non-CR conditions) were recovered on different days of culturing and subjected to centrifugation in Percoll density gradient to purify HD and LD cell populations. LPI concentrations were measured by LC-MS/MS. LPI concentrations in LD (**A**) and HD (**B**) cells are shown. Data are presented as means ± SD ($n = 2$; $^{*}p < 0.05$; $^{**}p < 0.01$). Abbreviation: Ctrl: control.

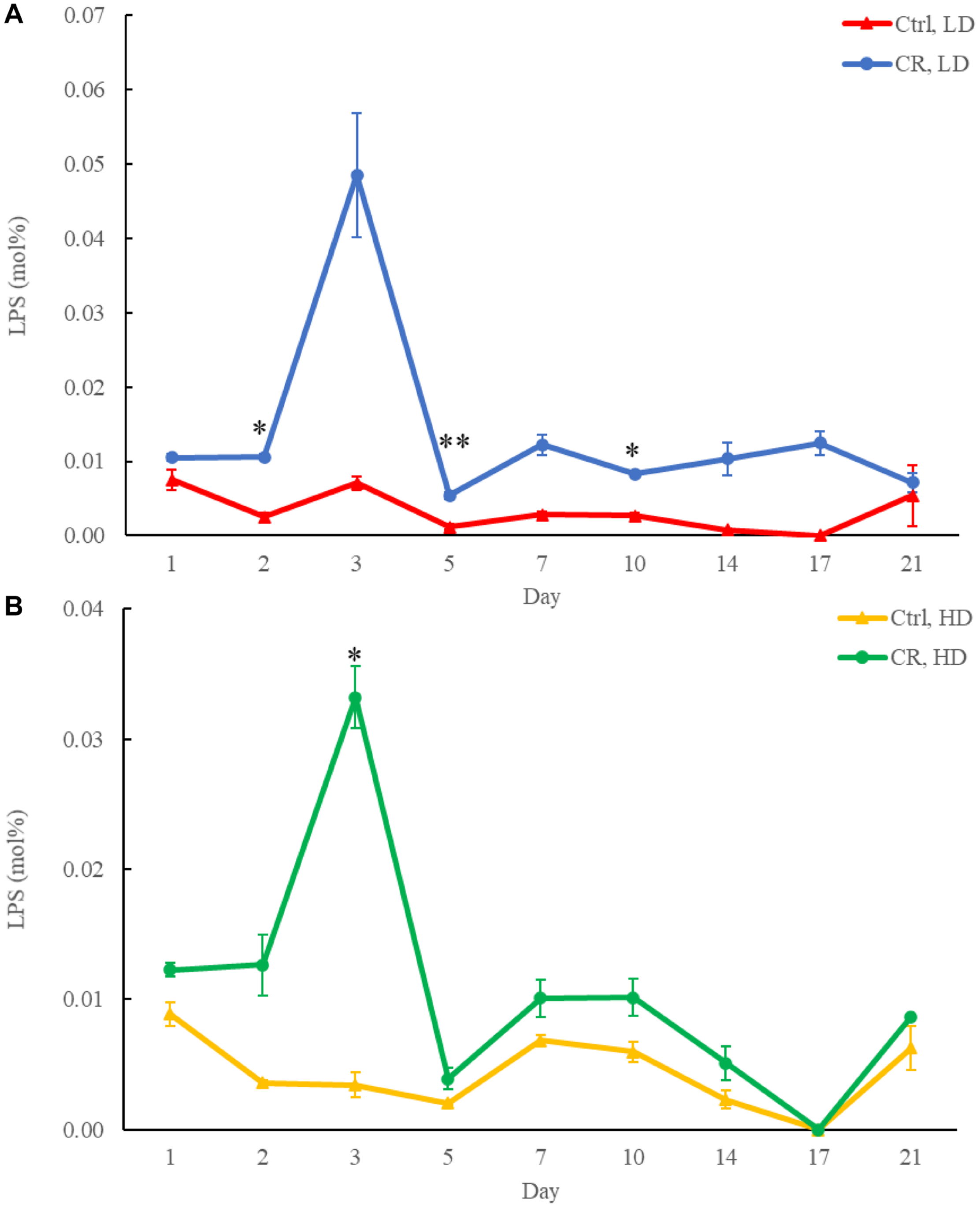

Supplementary Figure 14: CR increases LPS concentration in LD and to a lesser degree in HD cells through the chronological lifespan. Samples of WT yeast cultured in YP medium initially containing 0.2% glucose (CR conditions) or 2% glucose (control non-CR conditions) were recovered on different days of culturing and subjected to centrifugation in Percoll density gradient to purify HD and LD cell populations. LPS concentrations were measured by LC-MS/MS. LPS concentrations in LD (**A**) and HD (**B**) cells are shown. Data are presented as means ± SD ($n = 2$; $^{*}p < 0.05$; $^{**}p < 0.01$). Abbreviation: Ctrl: control.

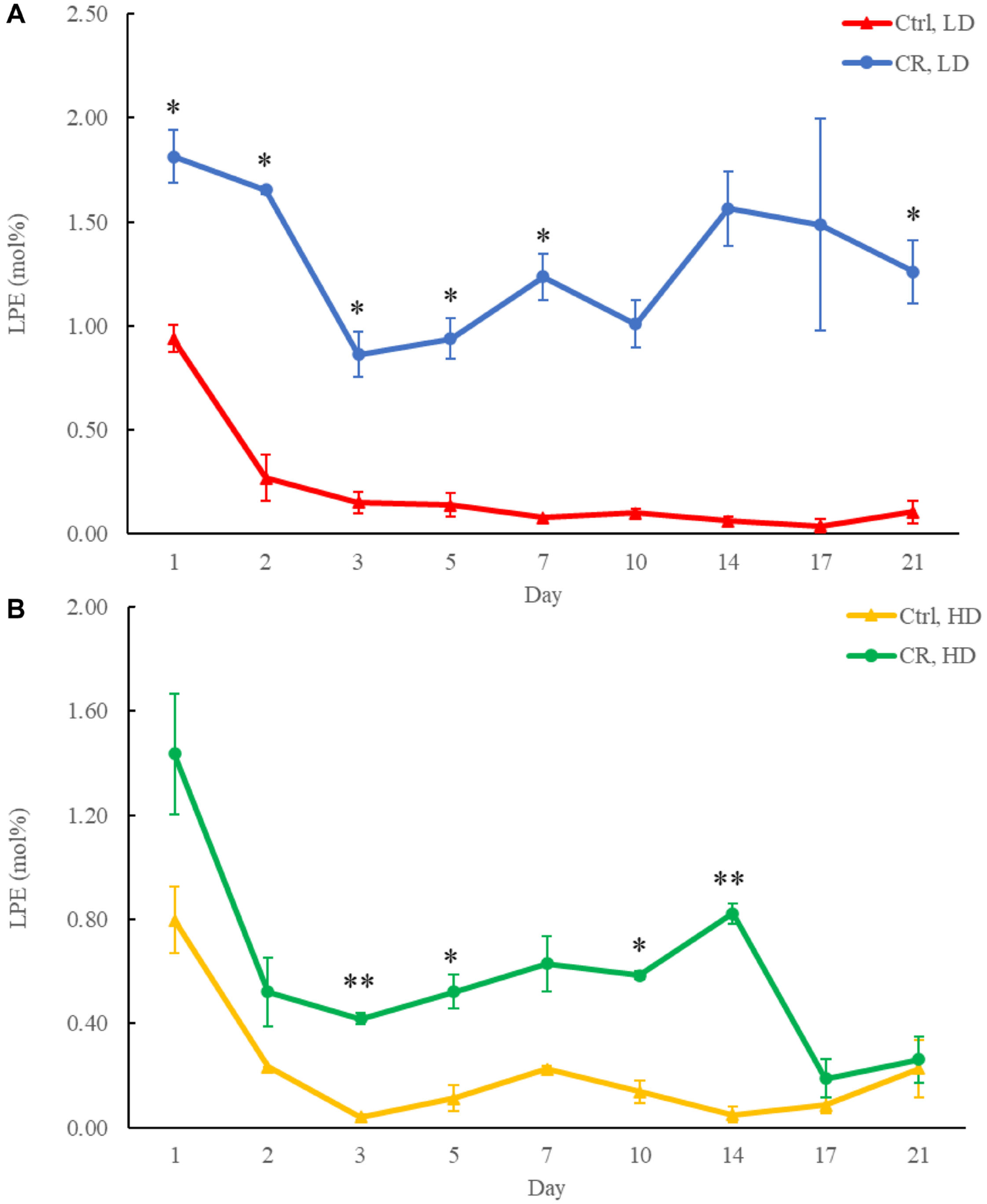

Supplementary Figure 15: CR significantly increases LPE concentration in HD and especially in LD cells throughout the chronological lifespan. Samples of WT yeast cultured in YP medium initially containing 0.2% glucose (CR conditions) or 2% glucose (control non-CR conditions) were recovered on different days of culturing and subjected to centrifugation in Percoll density gradient to purify HD and LD cell populations. LPE concentrations were measured by LC-MS/MS. LPE concentrations in LD (**A**) and HD (**B**) cells are shown. Data are presented as means ± SD ($n = 2$; $^{*}p < 0.05$; $^{**}p < 0.01$). Abbreviation: Ctrl: control.

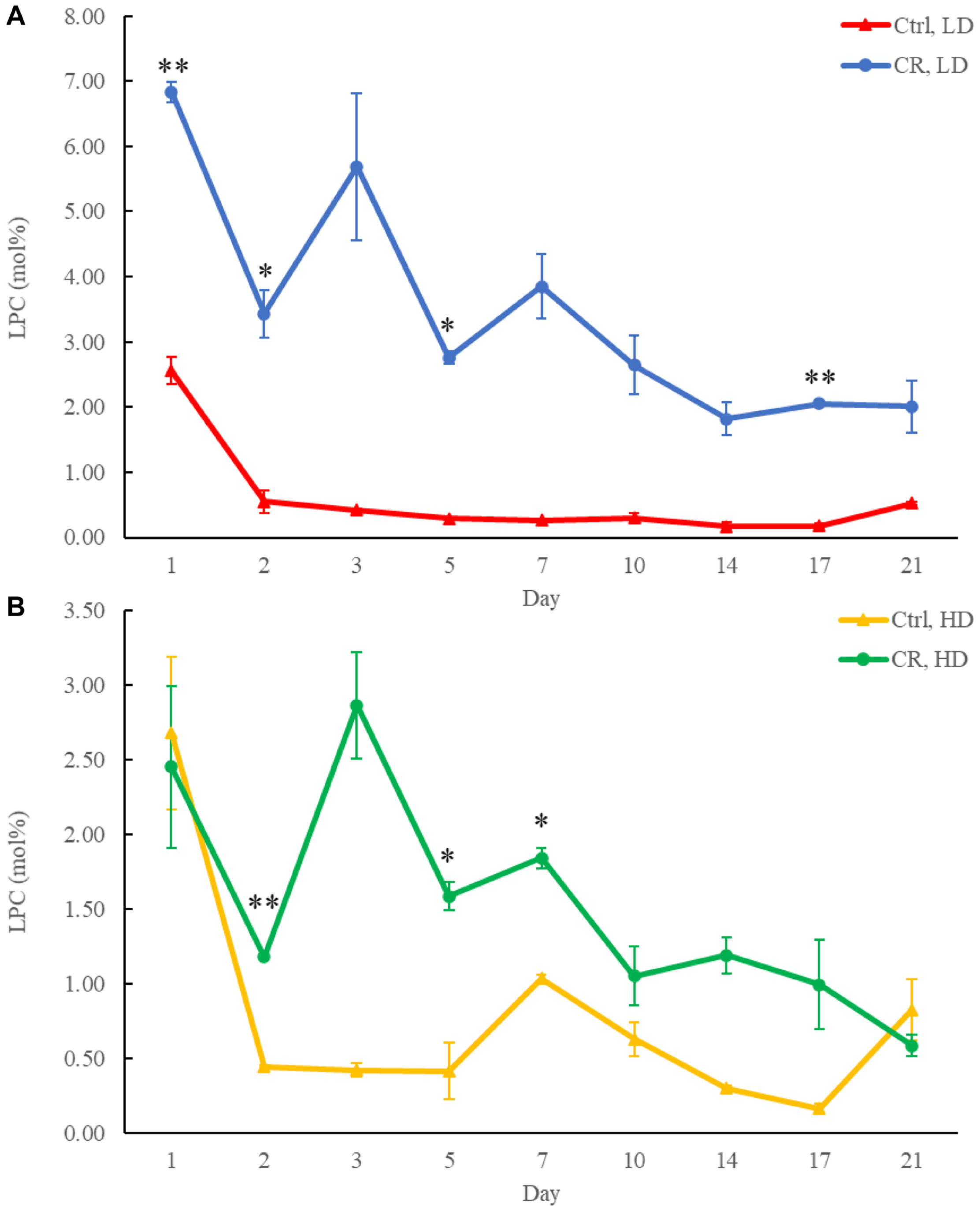

Supplementary Figure 16: CR significantly rises LPC concentration in both HD and LD cells through the chronological lifespan. Samples of WT yeast cultured in YP medium initially containing 0.2% glucose (CR conditions) or 2% glucose (control non-CR conditions) were recovered on different days of culturing and subjected to centrifugation in Percoll density gradient to purify HD and LD cell populations. LPC concentrations were measured by LC-MS/MS. LPC concentrations in LD (**A**) and HD (**B**) cells are shown. Data are presented as means ± SD ($n = 2$; $^{*}p < 0.05$; $^{**}p < 0.01$). Abbreviation: Ctrl: control.

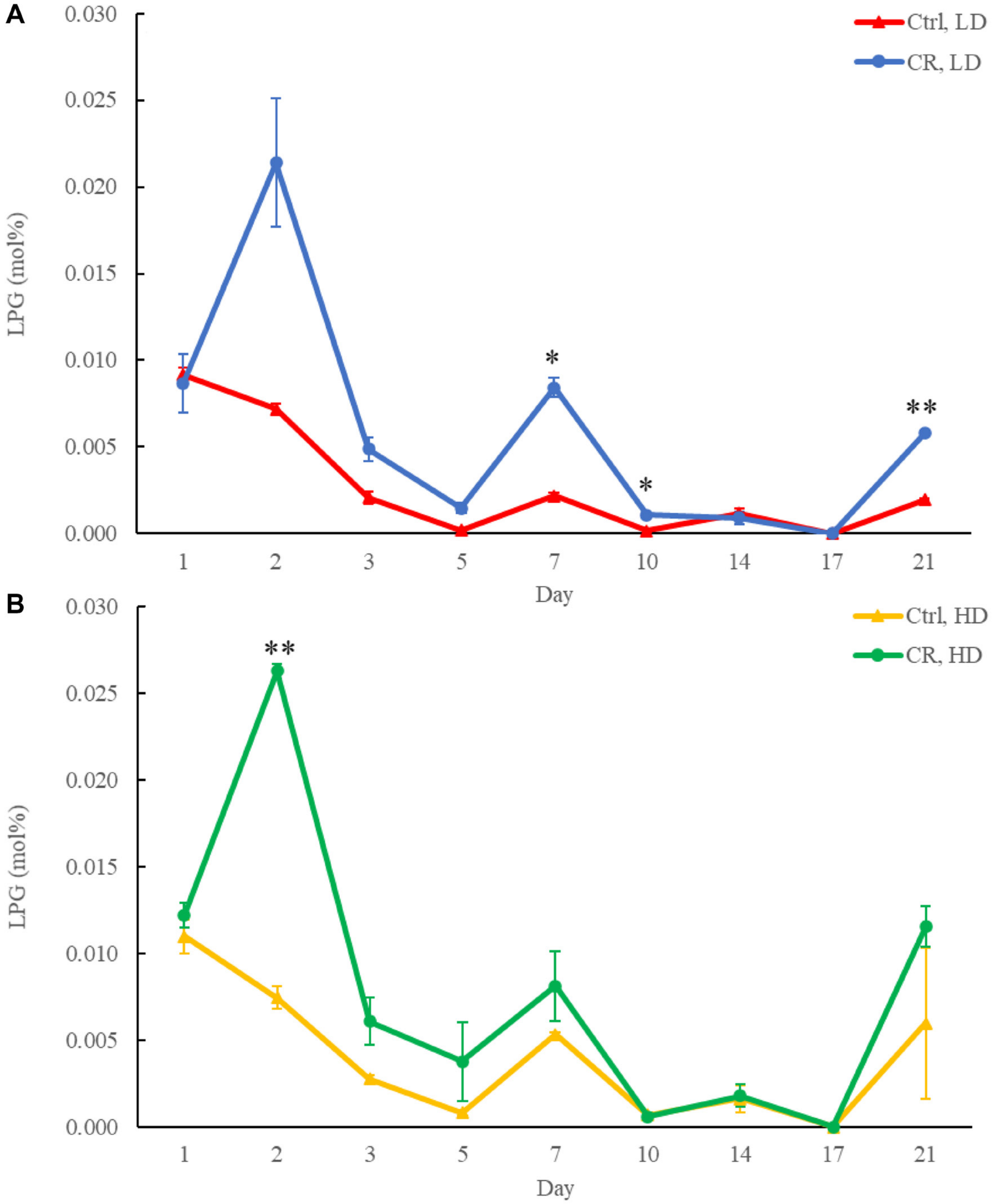

Supplementary Figure 17: CR increases LPG concentration in HD and LD cells through most of the chronological lifespan. Samples of WT yeast cultured in YP medium initially containing 0.2% glucose (CR conditions) or 2% glucose (control non-CR conditions) were recovered on different days of culturing and subjected to centrifugation in Percoll density gradient to purify HD and LD cell populations. LPG concentrations were measured by LC-MS/MS. LPG concentrations in LD (**A**) and HD (**B**) cells are shown. Data are presented as means ± SD ($n = 2$; $^{*}p < 0.05$; $^{**}p < 0.01$). Abbreviation: Ctrl: control.

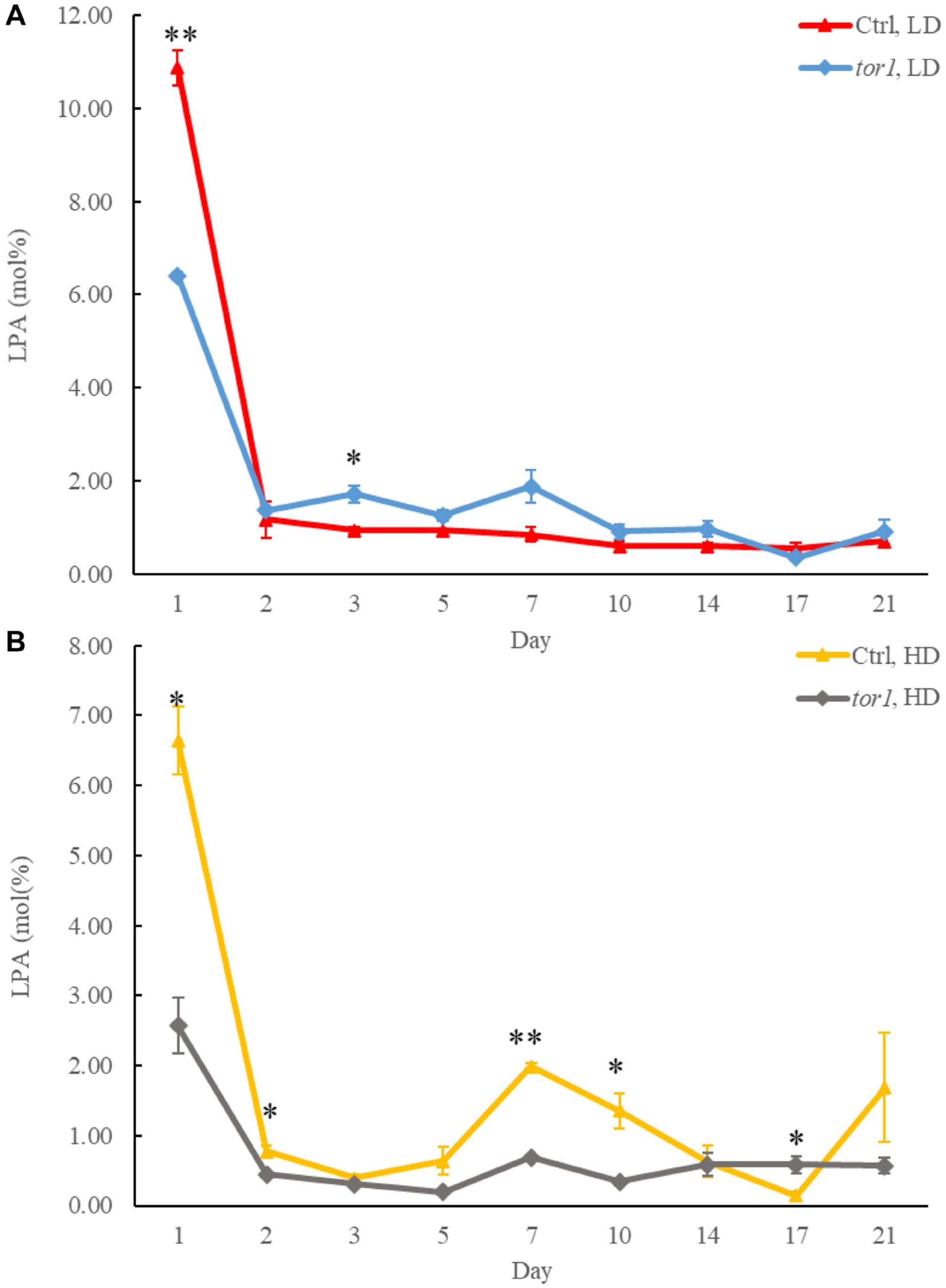

Supplementary Figure 18: The *tor1Δ* mutation does not have a significant long-lasting effect on LPA concentration in HD and LD cells through the chronological lifespan. Samples of WT (control) and *tor1Δ* yeast cultured in YP medium initially containing 2% glucose (non-CR conditions) were recovered on different days of culturing and subjected to centrifugation in Percoll density gradient to purify HD and LD cell populations. LPA concentrations were measured by LC-MS/MS. LPA concentrations in LD (**A**) and HD (**B**) cells are shown. Data are presented as means ± SD ($n = 2$; $^{*}p < 0.05$; $^{**}p < 0.01$). Abbreviation: Ctrl: control.

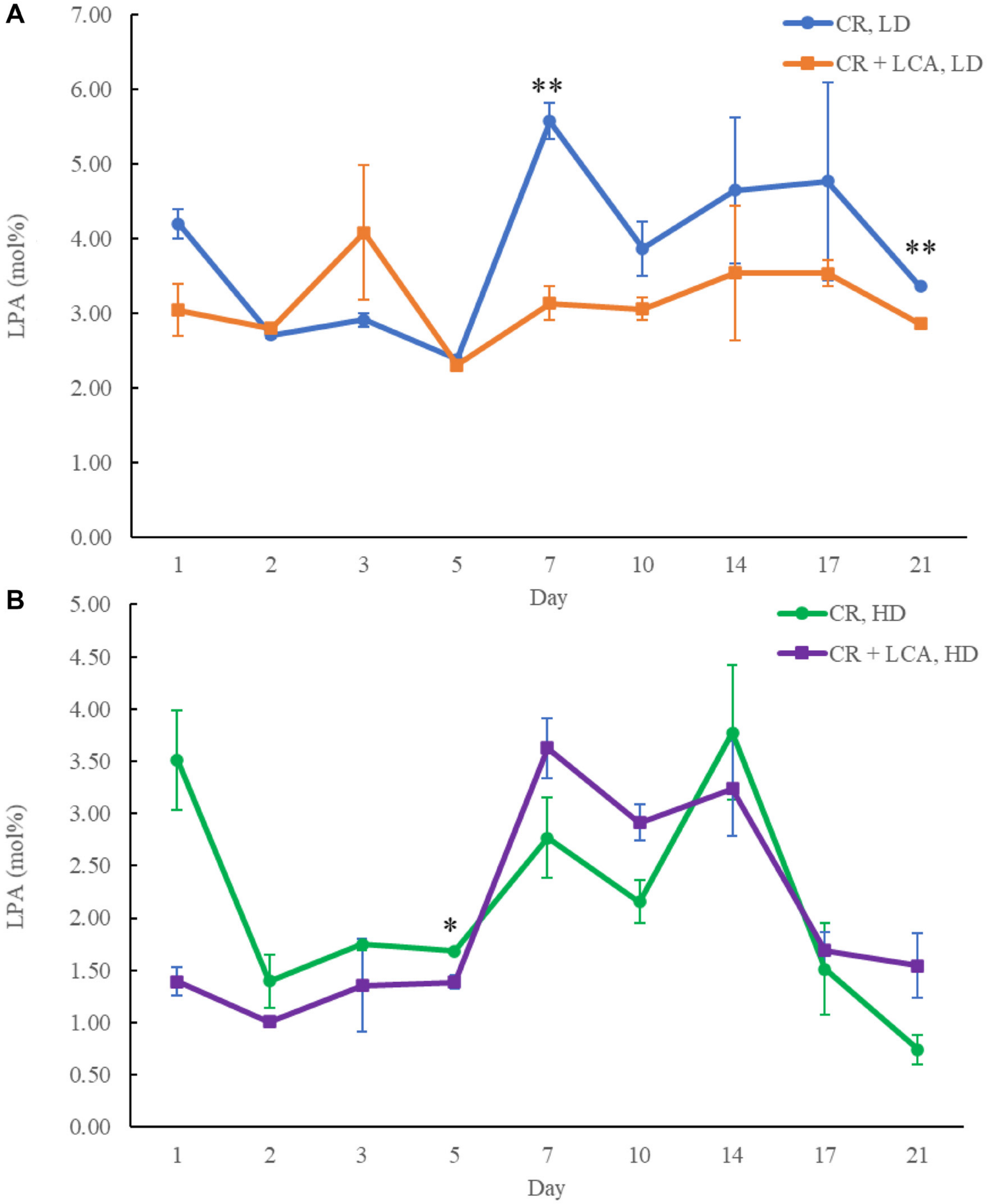

Supplementary Figure 19: LCA does not have a significant long-lasting effect on LPA concentration in HD and LD cells through the chronological lifespan. Samples of WT yeast cultured in YP medium initially containing 0.2% glucose (CR conditions) with 50 μM LCA or without it (control) were recovered on different days of culturing and subjected to centrifugation in Percoll density gradient to purify HD and LD cell populations. LPA concentrations were measured by LC-MS/MS. LPA concentrations in LD (**A**) and HD (**B**) cells are shown. Data are presented as means ± SD ($n = 2$; $^{*}p < 0.05$; $^{**}p < 0.01$).

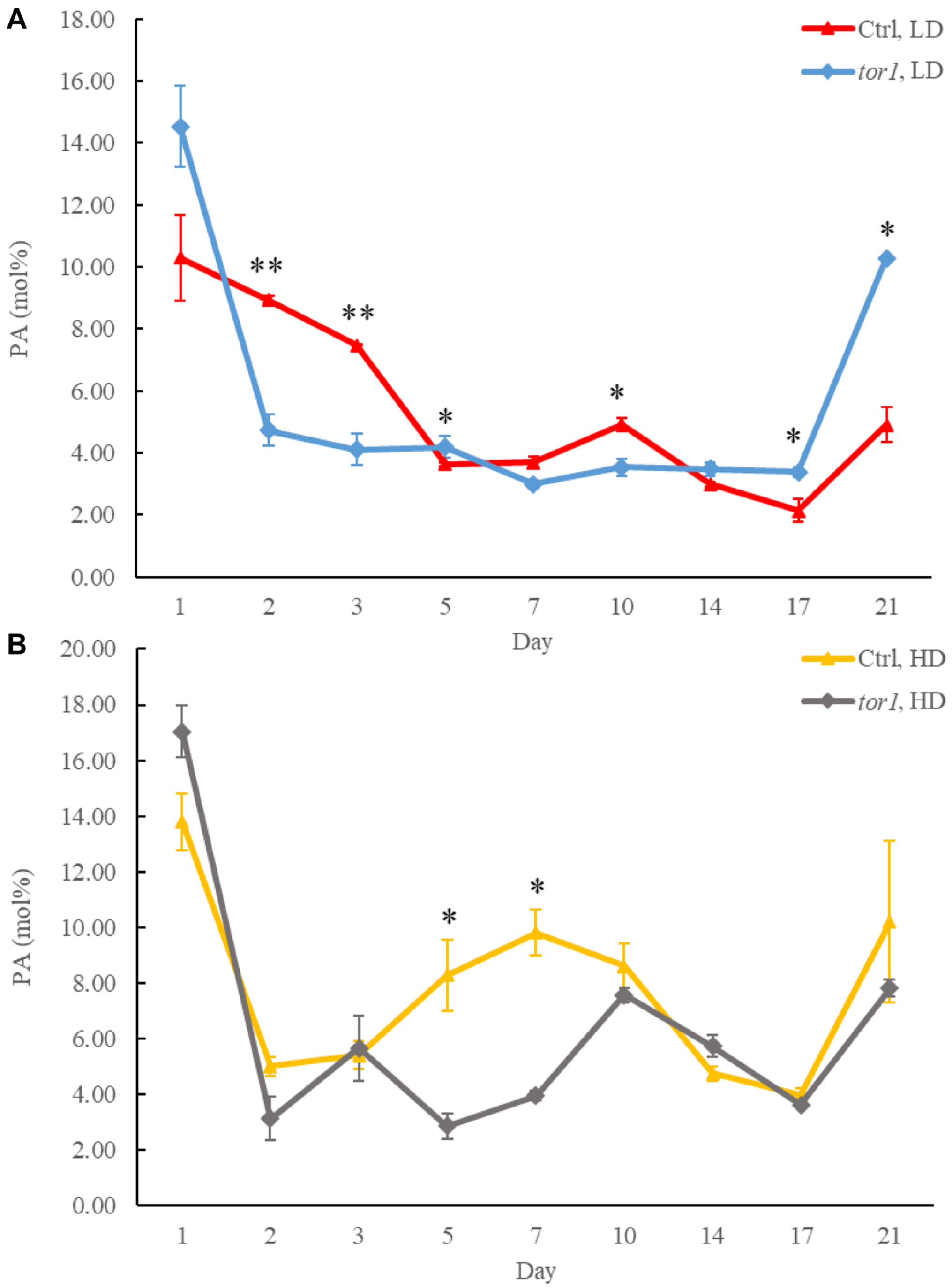

Supplementary Figure 20: The *tor1Δ* mutation does not have a significant long-lasting effect on PA concentration in HD and LD cells through the chronological lifespan. Samples of WT (control) and *tor1Δ* yeast cultured in YP medium initially containing 2% glucose (non-CR conditions) were recovered on different days of culturing and subjected to centrifugation in Percoll density gradient to purify HD and LD cell populations. PA concentrations were measured by LC-MS/MS. PA concentrations in LD (**A**) and HD (**B**) cells are shown. Data are presented as means ± SD ($n = 2$; $^{*}p < 0.05$; $^{**}p < 0.01$). Abbreviation: Ctrl: control.

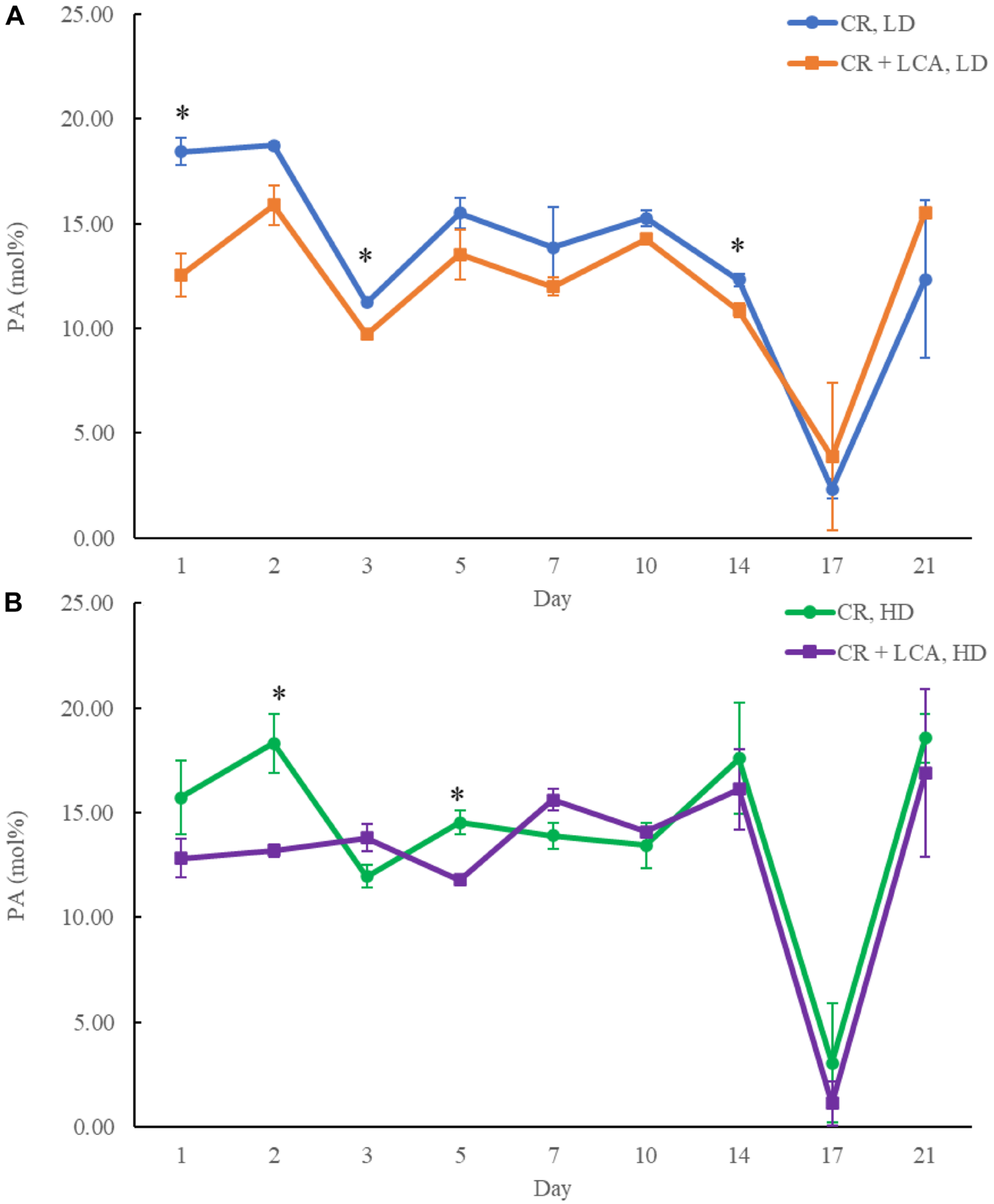

Supplementary Figure 21: LCA does not have a significant long-lasting effect on PA concentration in HD and LD cells through the chronological lifespan. Samples of WT yeast cultured in YP medium initially containing 0.2% glucose (CR conditions) with 50 µM LCA or without it (control) were recovered on different days of culturing and subjected to centrifugation in Percoll density gradient to purify HD and LD cell populations. PA concentrations were measured by LC-MS/MS. PA concentrations in LD (**A**) and HD (**B**) cells are shown. Data are presented as means ± SD ($n = 2$; $^{*}p < 0.05$).

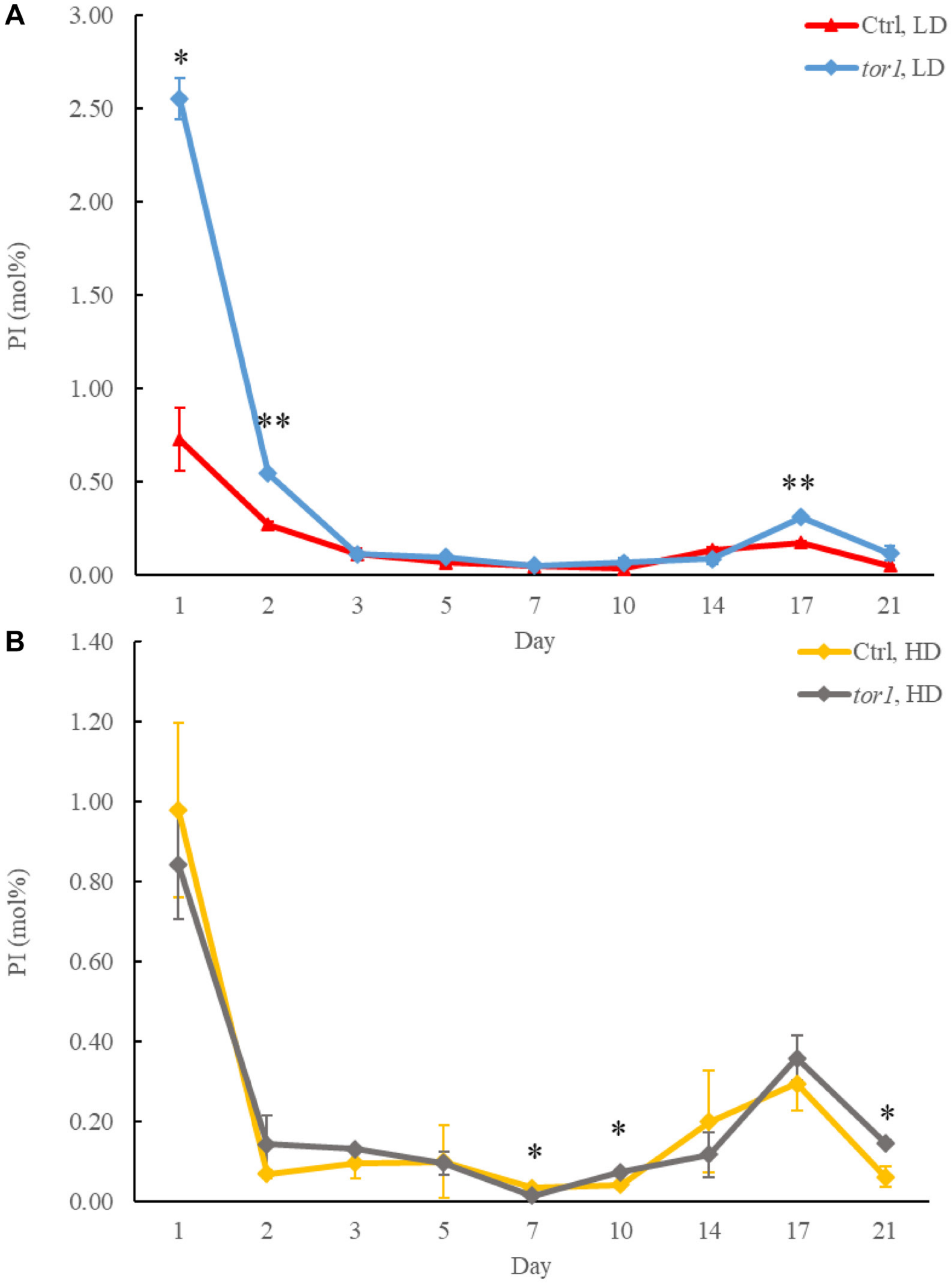

Supplementary Figure 22: The *tor1Δ* mutation does not have a significant long-lasting effect on PI concentration in HD and LD cells through the chronological lifespan. Samples of WT (control) and *tor1Δ* yeast cultured in YP medium initially containing 2% glucose (non-CR conditions) were recovered on different days of culturing and subjected to centrifugation in Percoll density gradient to purify HD and LD cell populations. PI concentrations were measured by LC-MS/MS. PI concentrations in LD (**A**) and HD (**B**) cells are shown. Data are presented as means ± SD ($n = 2$; $^{*}p < 0.05$; $^{**}p < 0.01$). Abbreviation: Ctrl: control.

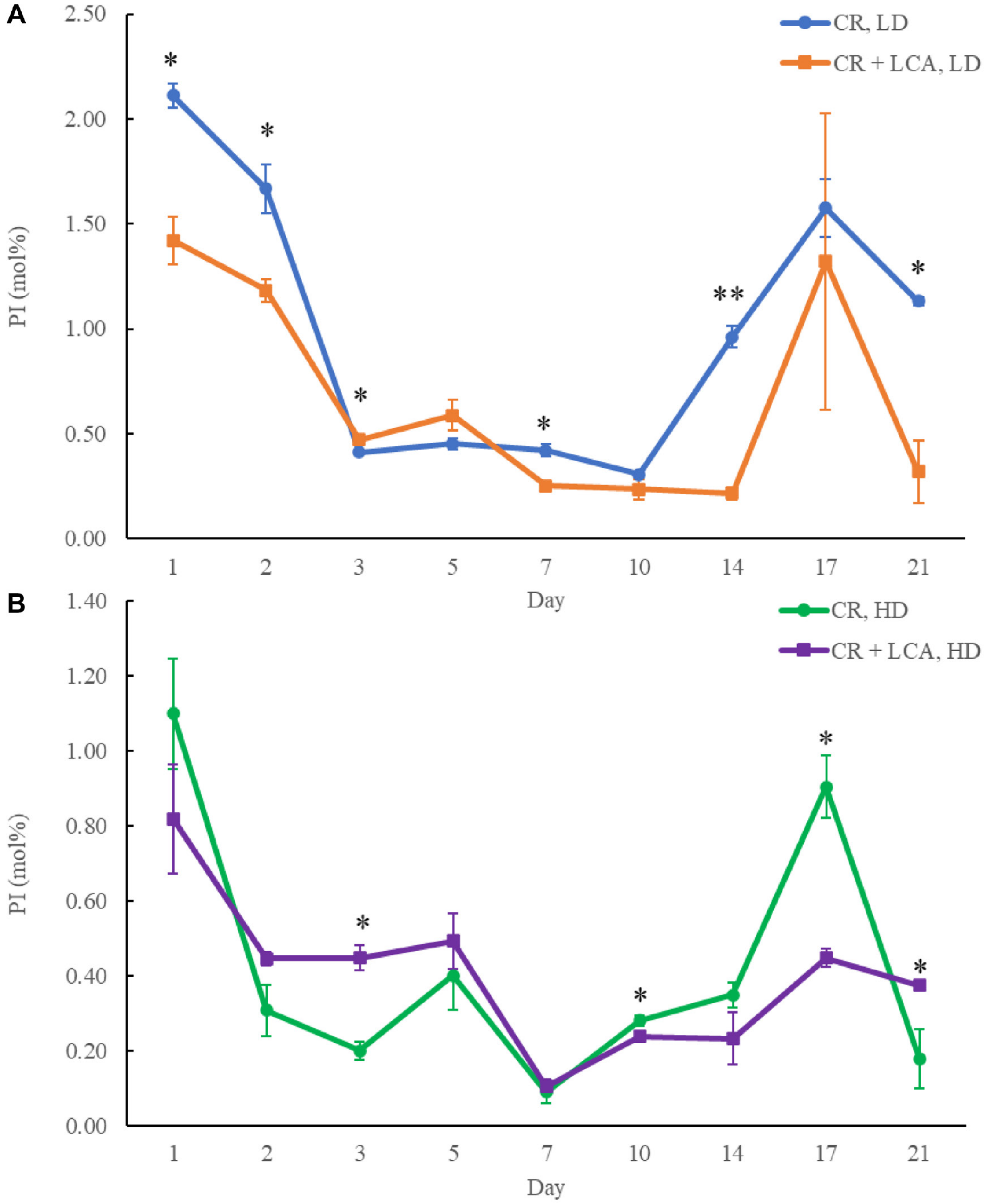

Supplementary Figure 23: LCA does not have a significant long-lasting effect on PI concentration in HD and LD cells through the chronological lifespan. Samples of WT yeast cultured in YP medium initially containing 0.2% glucose (CR conditions) with 50 µM LCA or without it (control) were recovered on different days of culturing and subjected to centrifugation in Percoll density gradient to purify HD and LD cell populations. PI concentrations were measured by LC-MS/MS. PI concentrations in LD (**A**) and HD (**B**) cells are shown. Data are presented as means ± SD ($n = 2$; $^{*}p < 0.05$).

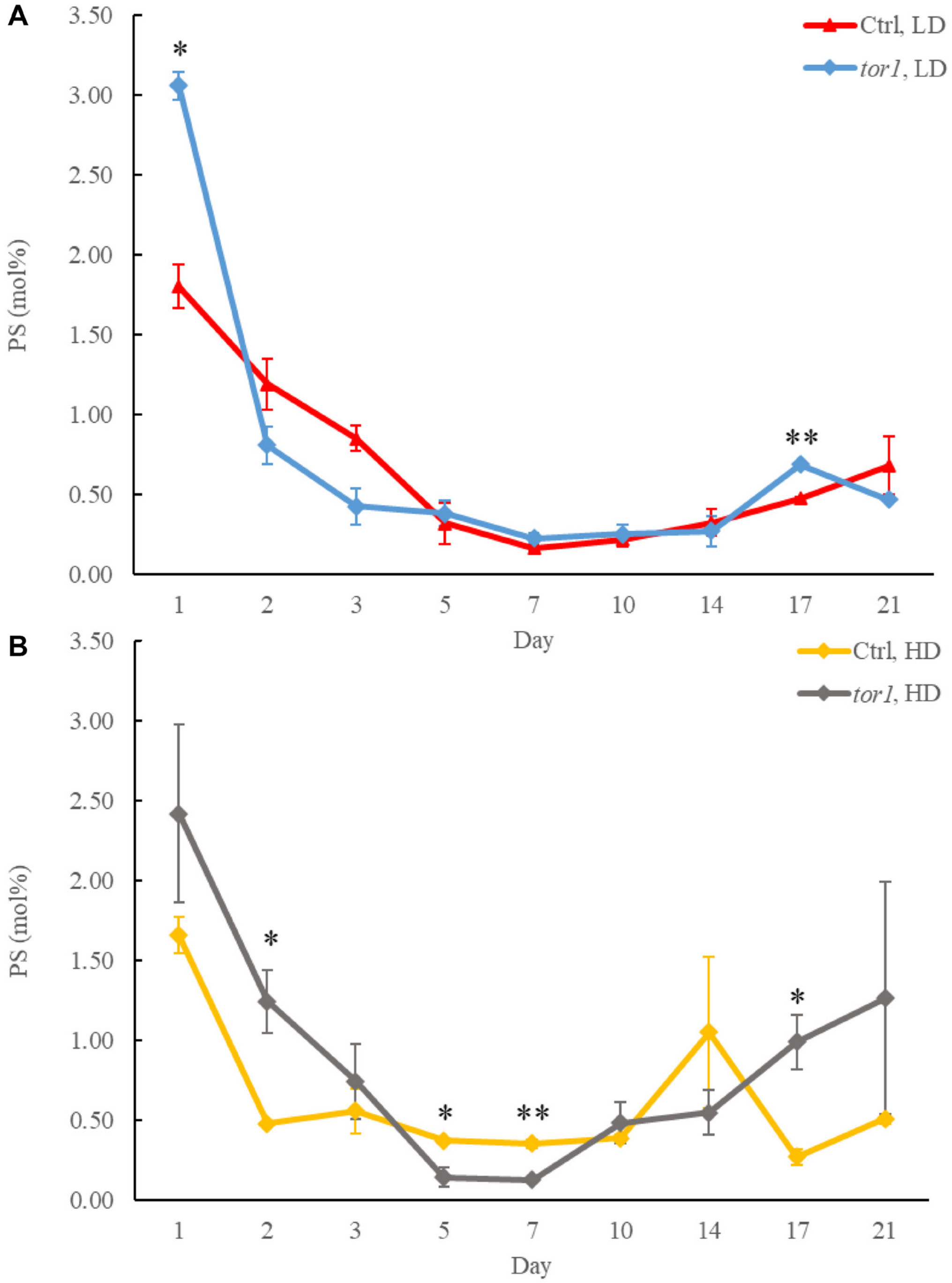

Supplementary Figure 24: The *tor1Δ* mutation does not have a significant long-lasting effect on PS concentration in HD and LD cells through the chronological lifespan. Samples of WT (control) and *tor1Δ* yeast cultured in YP medium initially containing 2% glucose (non-CR conditions) were recovered on different days of culturing and subjected to centrifugation in Percoll density gradient to purify HD and LD cell populations. PS concentrations were measured by LC-MS/MS. PS concentrations in LD (**A**) and HD (**B**) cells are shown. Data are presented as means ± SD ($n = 2$; $^{*}p < 0.05$; $^{**}p < 0.01$). Abbreviation: Ctrl: control.

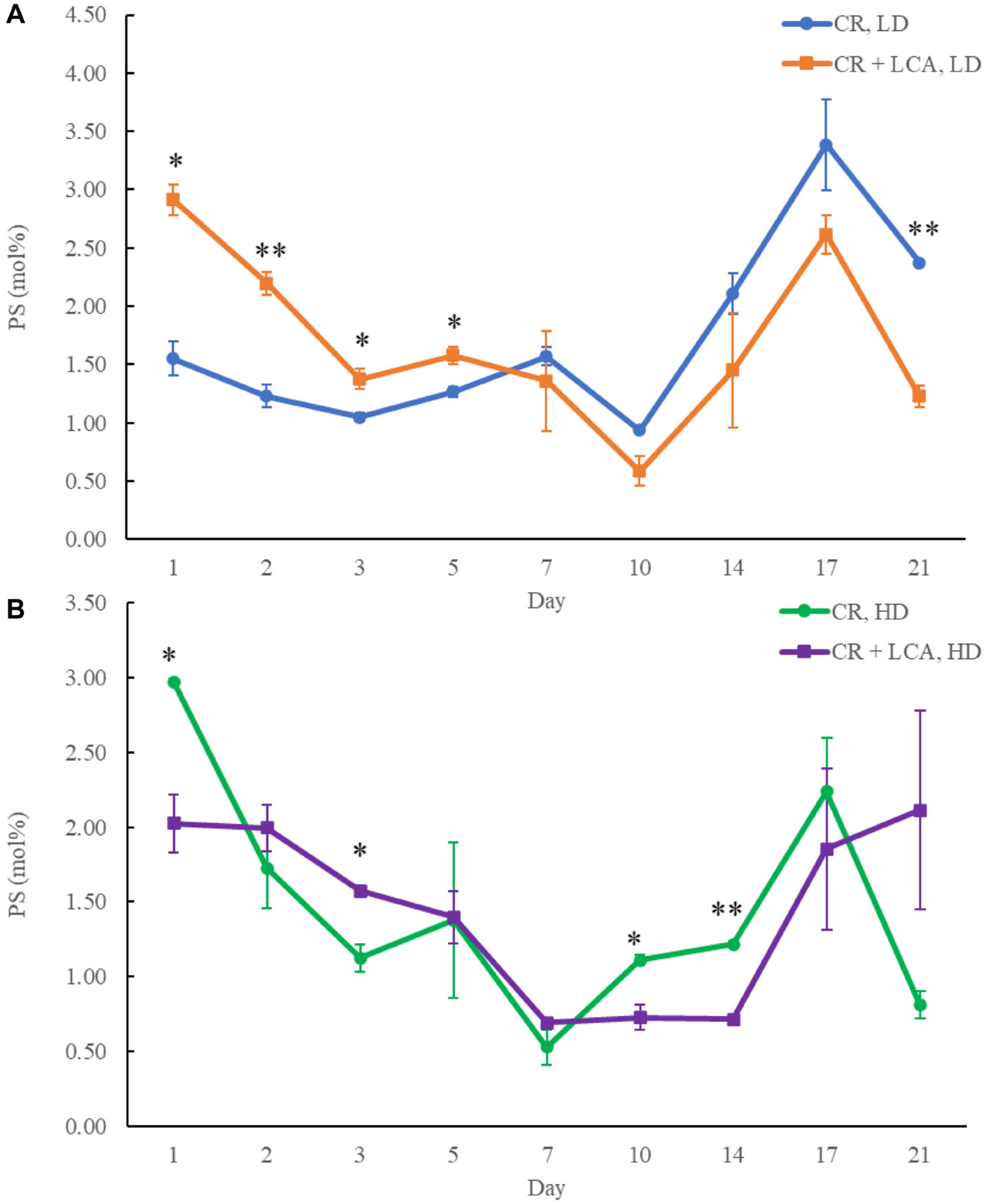

Supplementary Figure 25: LCA does not have a significant long-lasting effect on PS concentration in HD and LD cells through the chronological lifespan. Samples of WT yeast cultured in YP medium initially containing 0.2% glucose (CR conditions) with 50 µM LCA or without it (control) were recovered on different days of culturing and subjected to centrifugation in Percoll density gradient to purify HD and LD cell populations. PS concentrations were measured by LC-MS/MS. PS concentrations in LD (**A**) and HD (**B**) cells are shown. Data are presented as means ± SD ($n = 2$; $^{*}p < 0.05$).

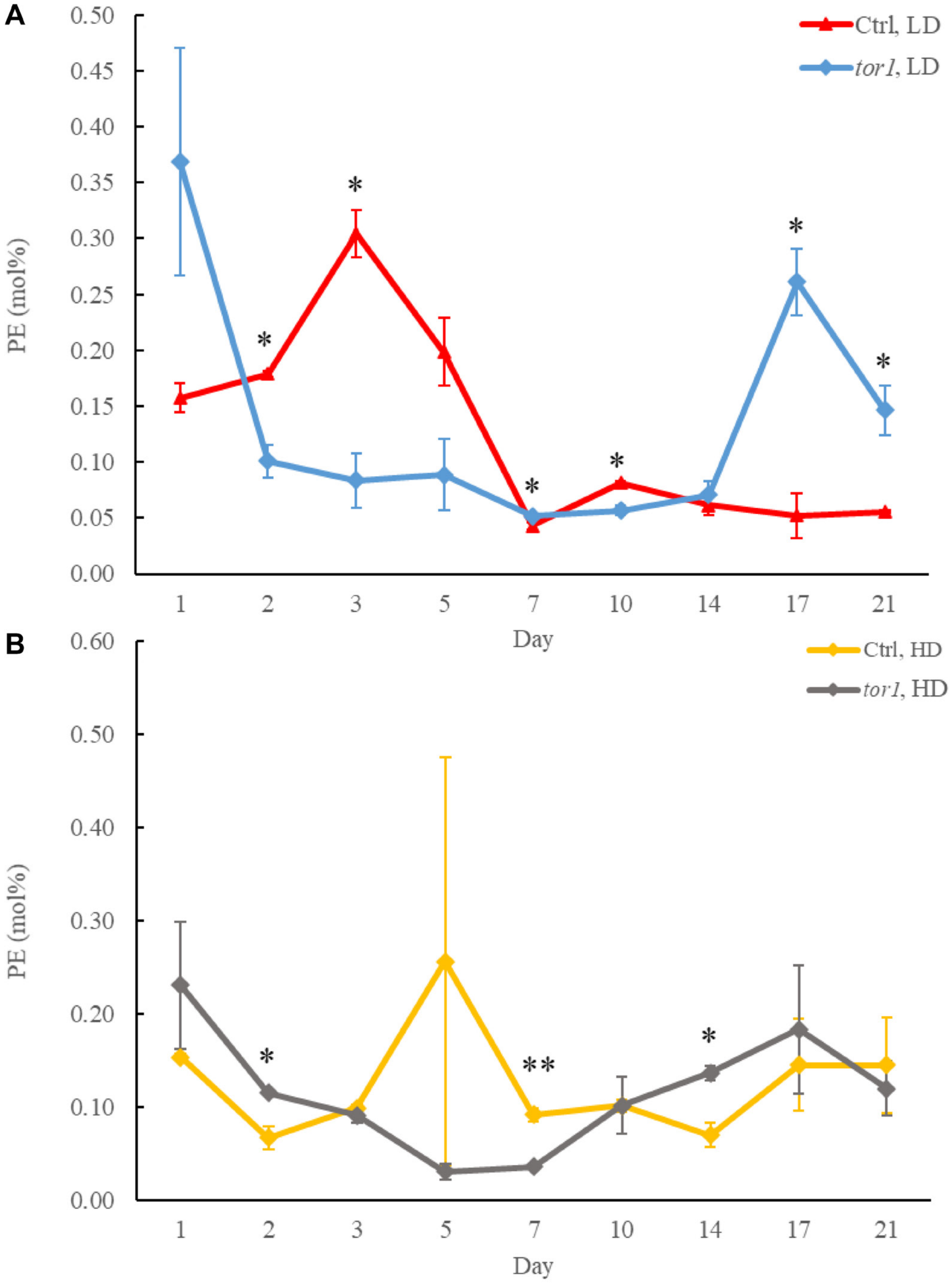

Supplementary Figure 26: The *tor1Δ* mutation does not have a significant long-lasting effect on PE concentration in HD and LD cells through the chronological lifespan. Samples of WT (control) and *tor1Δ* yeast cultured in YP medium initially containing 2% glucose (non-CR conditions) were recovered on different days of culturing and subjected to centrifugation in Percoll density gradient to purify HD and LD cell populations. PE concentrations were measured by LC-MS/MS. PE concentrations in LD (**A**) and HD (**B**) cells are shown. Data are presented as means ± SD ($n = 2$; $^{*}p < 0.05$; $^{**}p < 0.01$). Abbreviation: Ctrl: control.

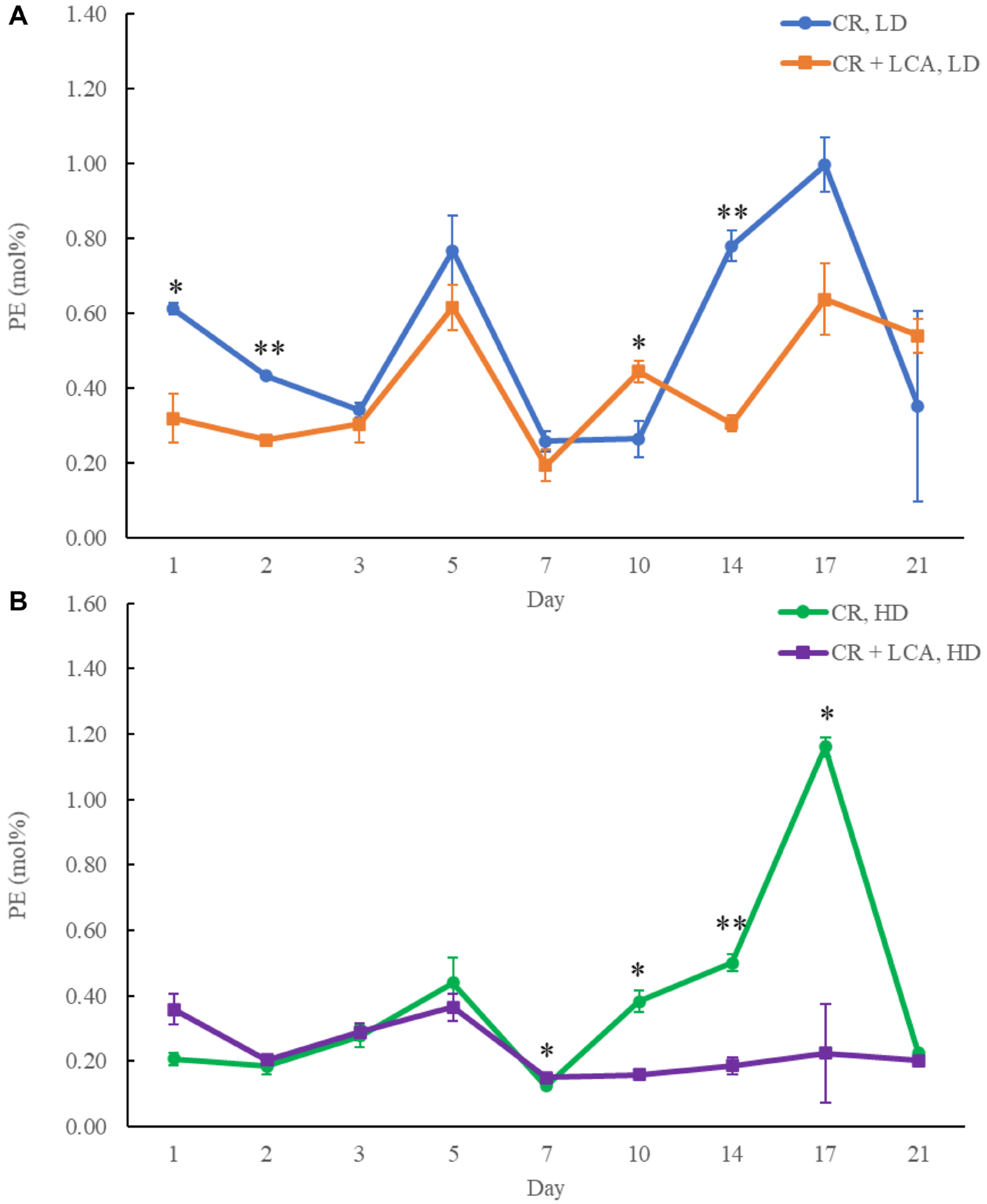

Supplementary Figure 27: LCA does not have a significant long-lasting effect on PE concentration in HD and LD cells through the chronological lifespan. Samples of WT yeast cultured in YP medium initially containing 0.2% glucose (CR conditions) with 50 µM LCA or without it (control) were recovered on different days of culturing and subjected to centrifugation in Percoll density gradient to purify HD and LD cell populations. PE concentrations were measured by LC-MS/MS. PE concentrations in LD (**A**) and HD (**B**) cells are shown. Data are presented as means ± SD ($n = 2$; $^{*}p < 0.05$; $^{**}p < 0.01$).

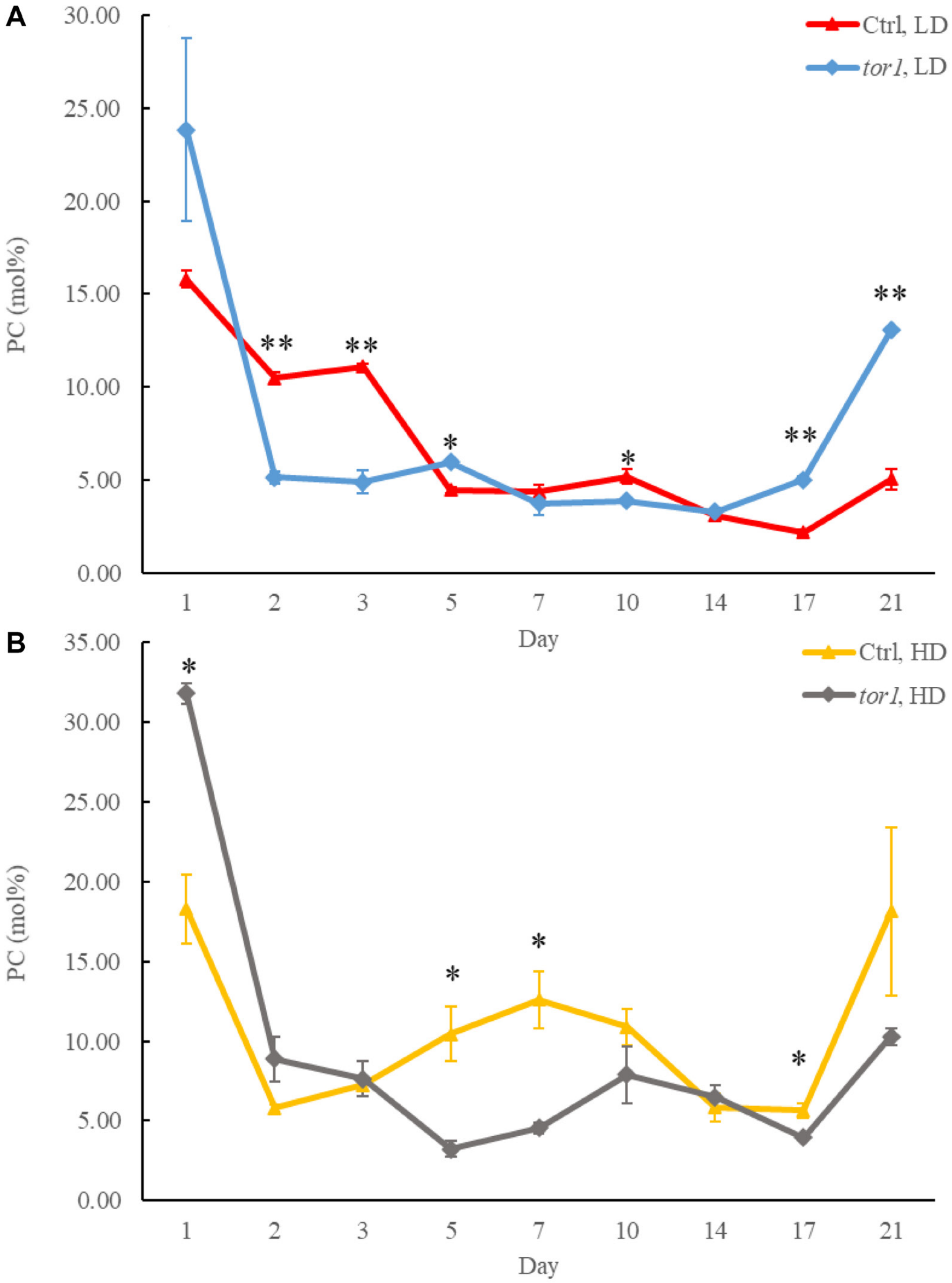

Supplementary Figure 28: The *tor1Δ* mutation does not have a significant long-lasting effect on PC concentration in HD and LD cells through the chronological lifespan. Samples of WT (control) and *tor1Δ* yeast cultured in YP medium initially containing 2% glucose (non-CR conditions) were recovered on different days of culturing and subjected to centrifugation in Percoll density gradient to purify HD and LD cell populations. PC concentrations were measured by LC-MS/MS. PC concentrations in LD (**A**) and HD (**B**) cells are shown. Data are presented as means ± SD ($n = 2$; $^{*}p < 0.05$; $^{**}p < 0.01$). Abbreviation: Ctrl: control.

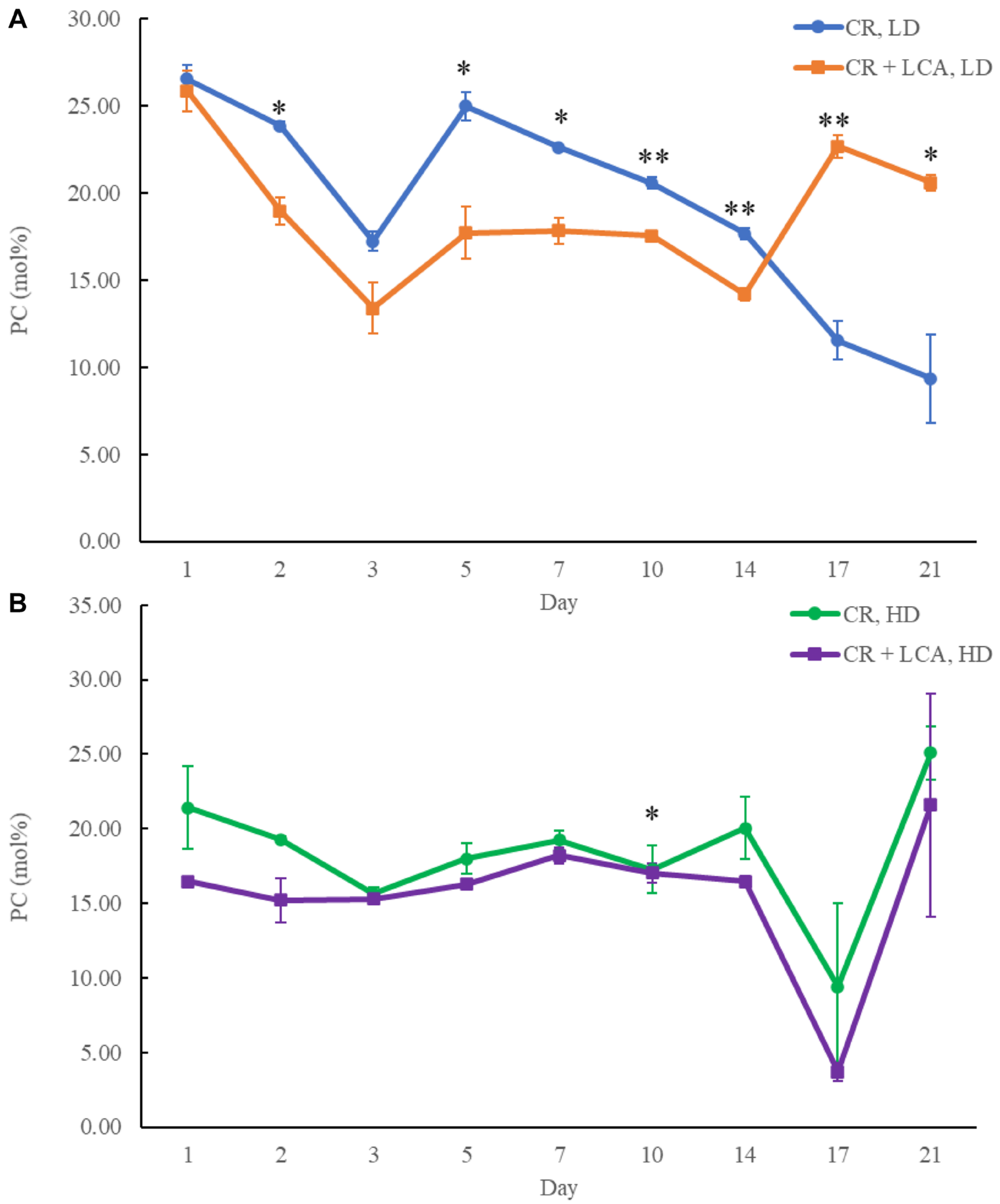

Supplementary Figure 29: LCA does not have a significant long-lasting effect on PC concentration in HD and LD cells through the chronological lifespan. Samples of WT yeast cultured in YP medium initially containing 0.2% glucose (CR conditions) with 50 μM LCA or without it (control) were recovered on different days of culturing and subjected to centrifugation in Percoll density gradient to purify HD and LD cell populations. PC concentrations were measured by LC-MS/MS. PC concentrations in LD (**A**) and HD (**B**) cells are shown. Data are presented as means ± SD ($n = 2$; $^{*}p < 0.05$; $^{**}p < 0.01$).

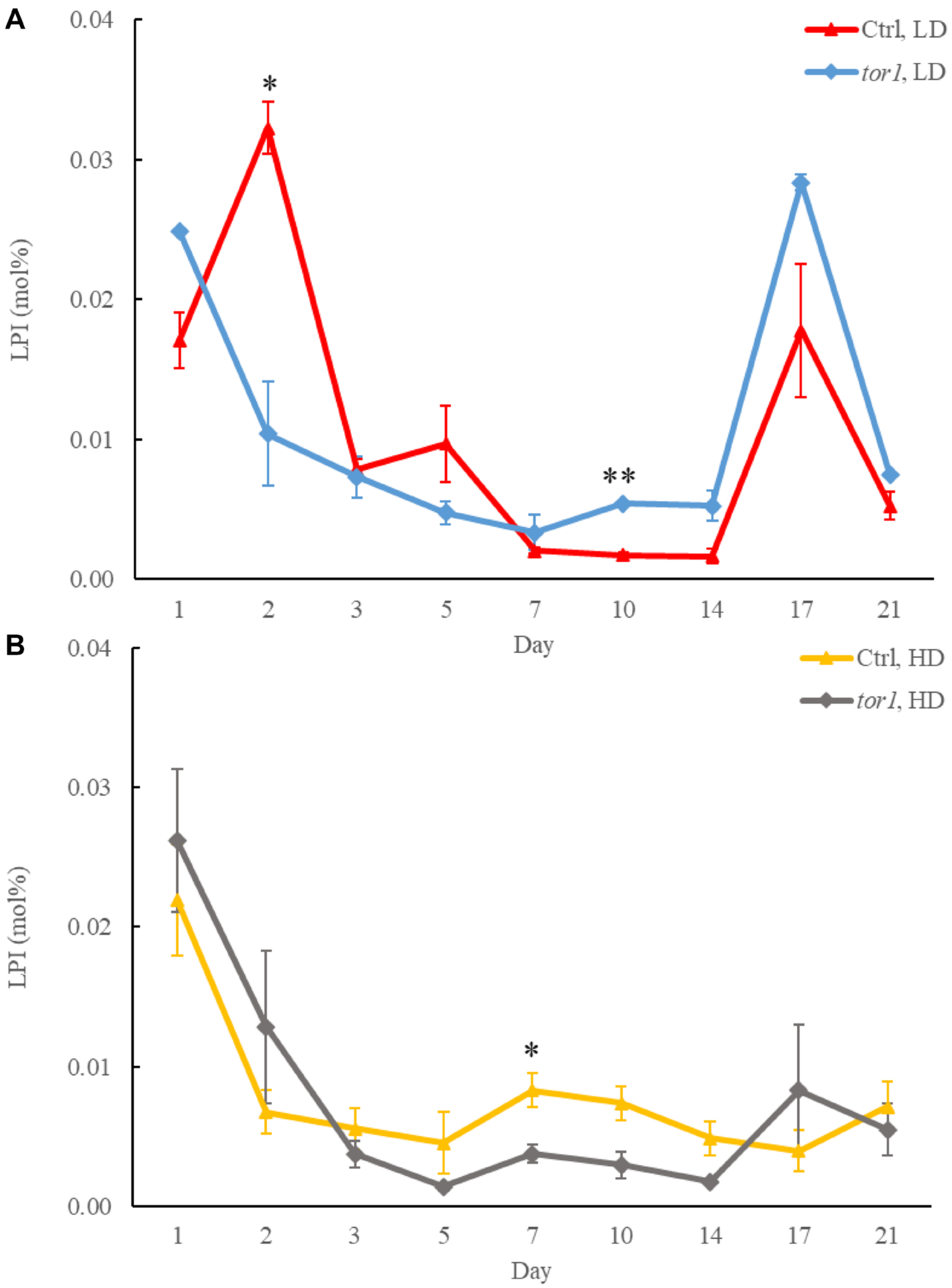

Supplementary Figure 30: The *tor1Δ* mutation does not have a significant long-lasting effect on LPI concentration in HD and LD cells through the chronological lifespan. Samples of WT (control) and *tor1Δ* yeast cultured in YP medium initially containing 2% glucose (non-CR conditions) were recovered on different days of culturing and subjected to centrifugation in Percoll density gradient to purify HD and LD cell populations. LPI concentrations were measured by LC-MS/MS. LPI concentrations in LD (**A**) and HD (**B**) cells are shown. Data are presented as means ± SD ($n = 2$; $^{*}p < 0.05$; $^{**}p < 0.01$). Abbreviation: Ctrl: control.

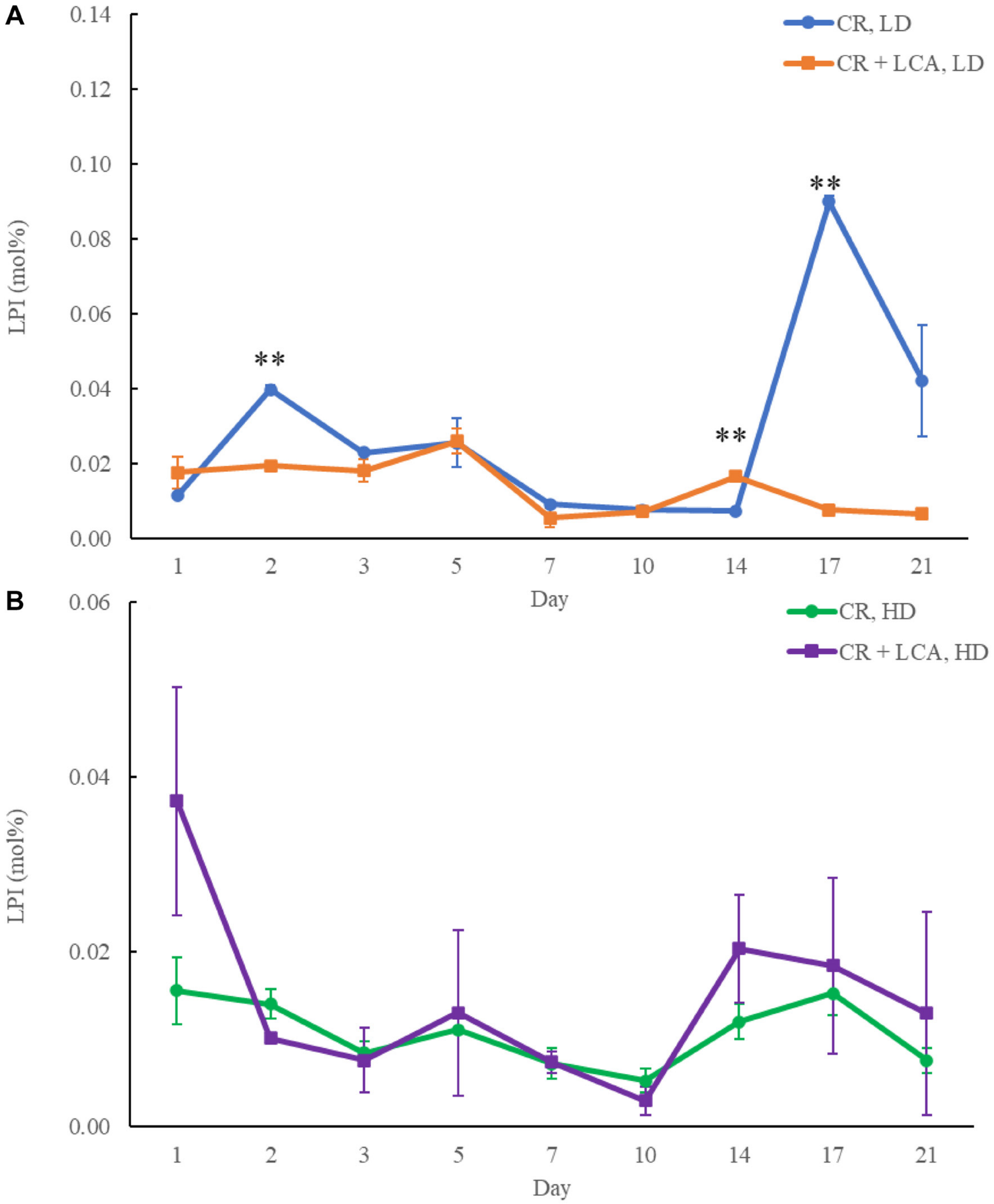

Supplementary Figure 31: LCA does not have a significant long-lasting effect on LPI concentration in HD and LD cells through the chronological lifespan. Samples of WT yeast cultured in YP medium initially containing 0.2% glucose (CR conditions) with 50 μM LCA or without it (control) were recovered on different days of culturing and subjected to centrifugation in Percoll density gradient to purify HD and LD cell populations. LPI concentrations were measured by LC-MS/MS. LPI concentrations in LD (**A**) and HD (**B**) cells are shown. Data are presented as means ± SD ($n = 2$; $^{**}p < 0.01$).

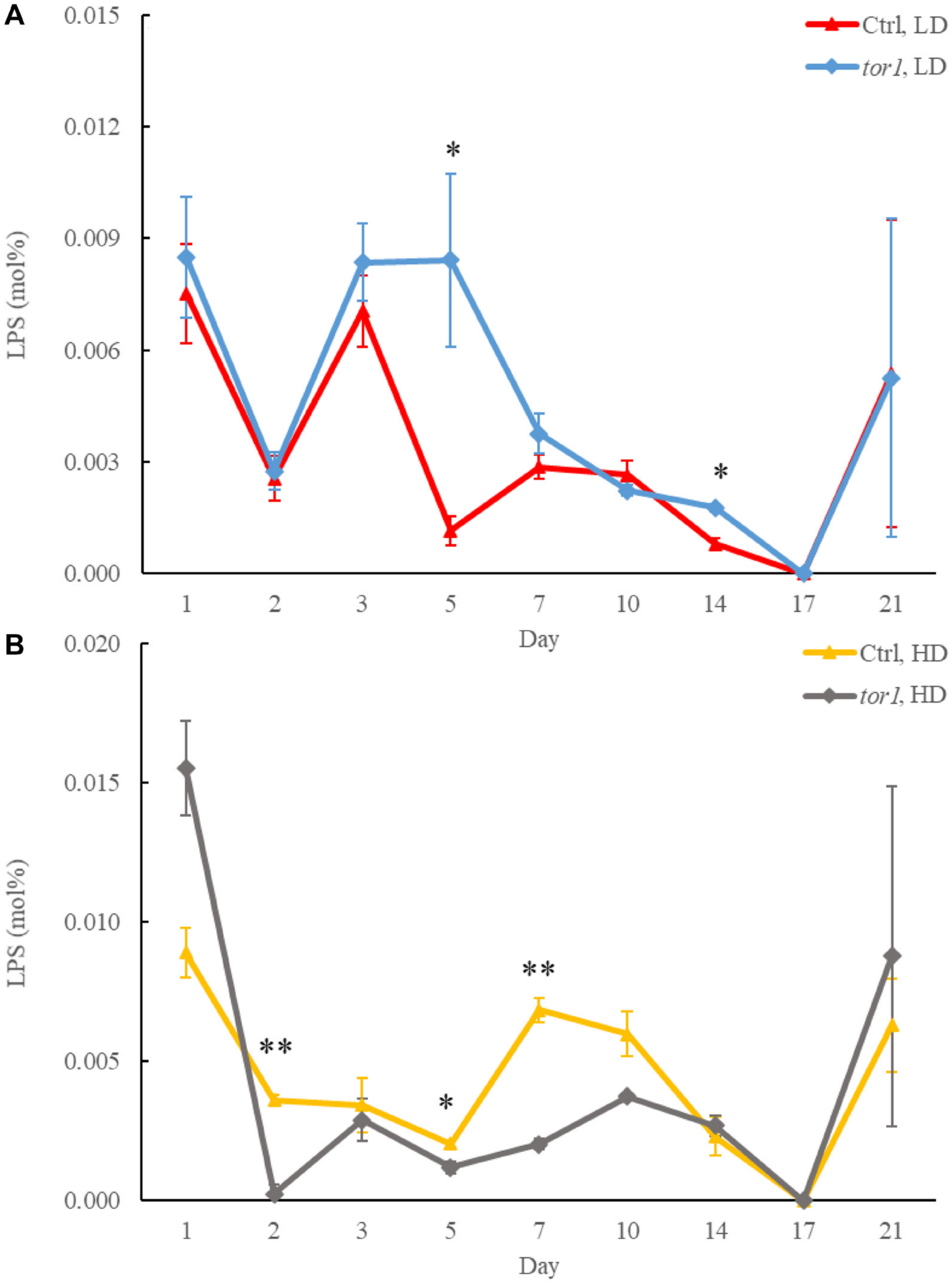

Supplementary Figure 32: The *tor1Δ* mutation does not have a significant long-lasting effect on LPS concentration in HD and LD cells through the chronological lifespan. Samples of WT (control) and *tor1Δ* yeast cultured in YP medium initially containing 2% glucose (non-CR conditions) were recovered on different days of culturing and subjected to centrifugation in Percoll density gradient to purify HD and LD cell populations. LPS concentrations were measured by LC-MS/MS. LPS concentrations in LD (**A**) and HD (**B**) cells are shown. Data are presented as means ± SD ($n = 2$; $^{*}p < 0.05$; $^{**}p < 0.01$). Abbreviation: Ctrl: control.

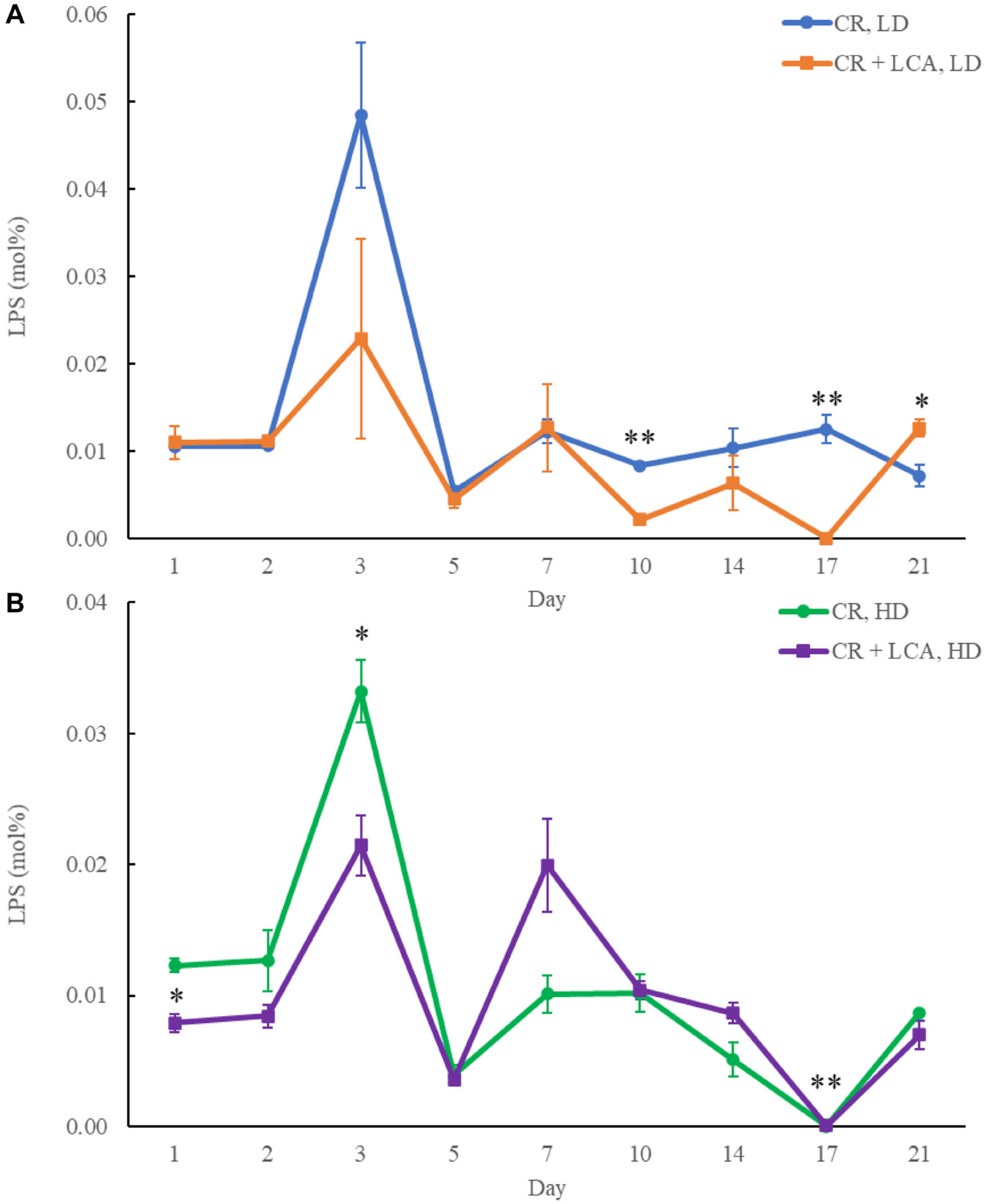

Supplementary Figure 33: LCA does not have a significant long-lasting effect on LPS concentration in HD and LD cells through the chronological lifespan. Samples of WT yeast cultured in YP medium initially containing 0.2% glucose (CR conditions) with 50 µM LCA or without it (control) were recovered on different days of culturing and subjected to centrifugation in Percoll density gradient to purify HD and LD cell populations. LPS concentrations were measured by LC-MS/MS. LPS concentrations in LD (**A**) and HD (**B**) cells are shown. Data are presented as means ± SD ($n = 2$; $^{*}p < 0.05$; $^{**}p < 0.01$).

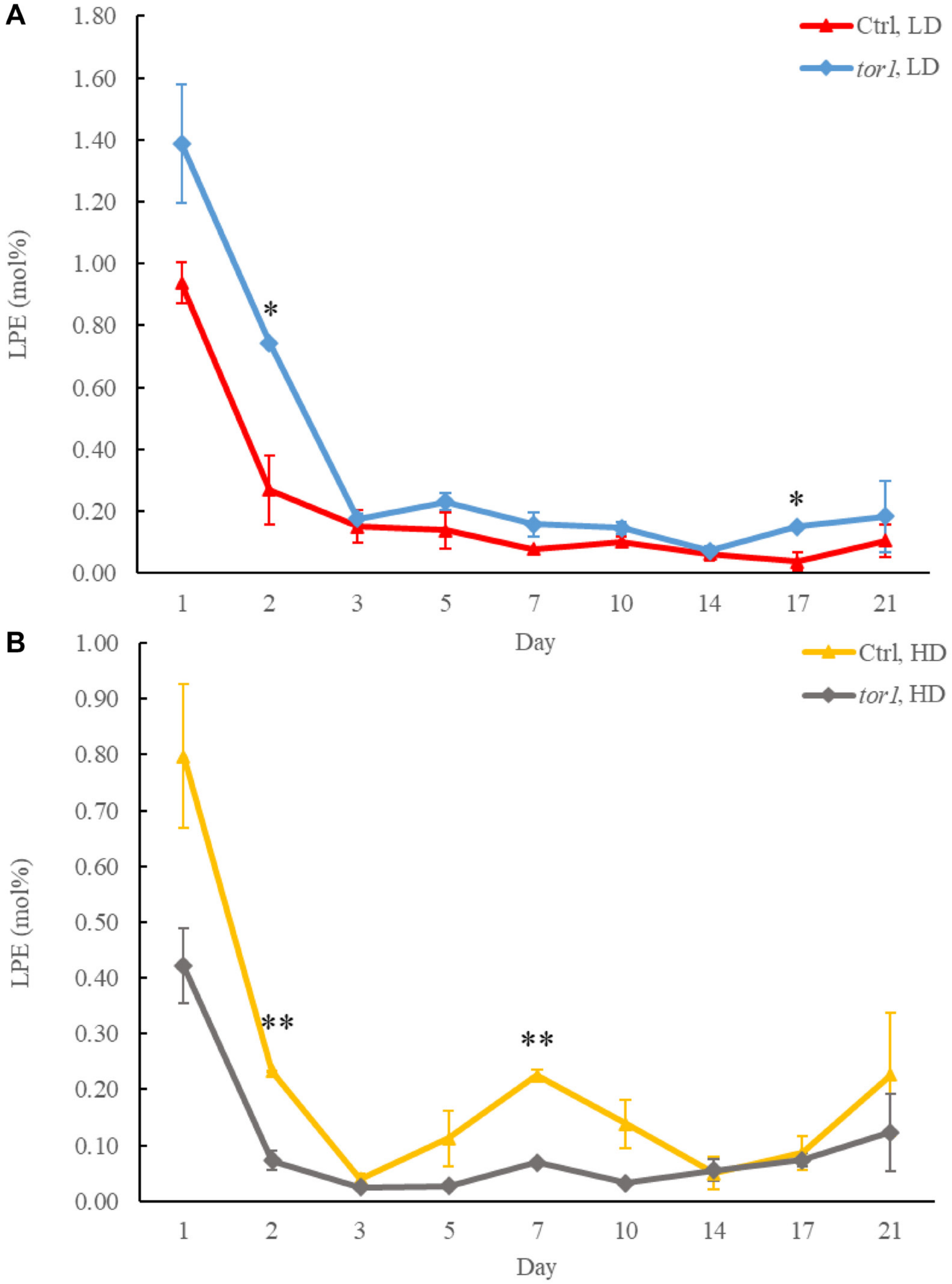

Supplementary Figure 34: The *tor1Δ* mutation does not have a significant long-lasting effect on LPE concentration in HD and LD cells through the chronological lifespan. Samples of WT (control) and *tor1Δ* yeast cultured in YP medium initially containing 2% glucose (non-CR conditions) were recovered on different days of culturing and subjected to centrifugation in Percoll density gradient to purify HD and LD cell sub-populations. LPE concentrations were measured by LC-MS/MS. LPE concentrations in LD (**A**) and HD (**B**) cells are shown. Data are presented as means ± SD ($n = 2$; $^{*}p < 0.05$; $^{**}p < 0.01$). Abbreviation: Ctrl: control.

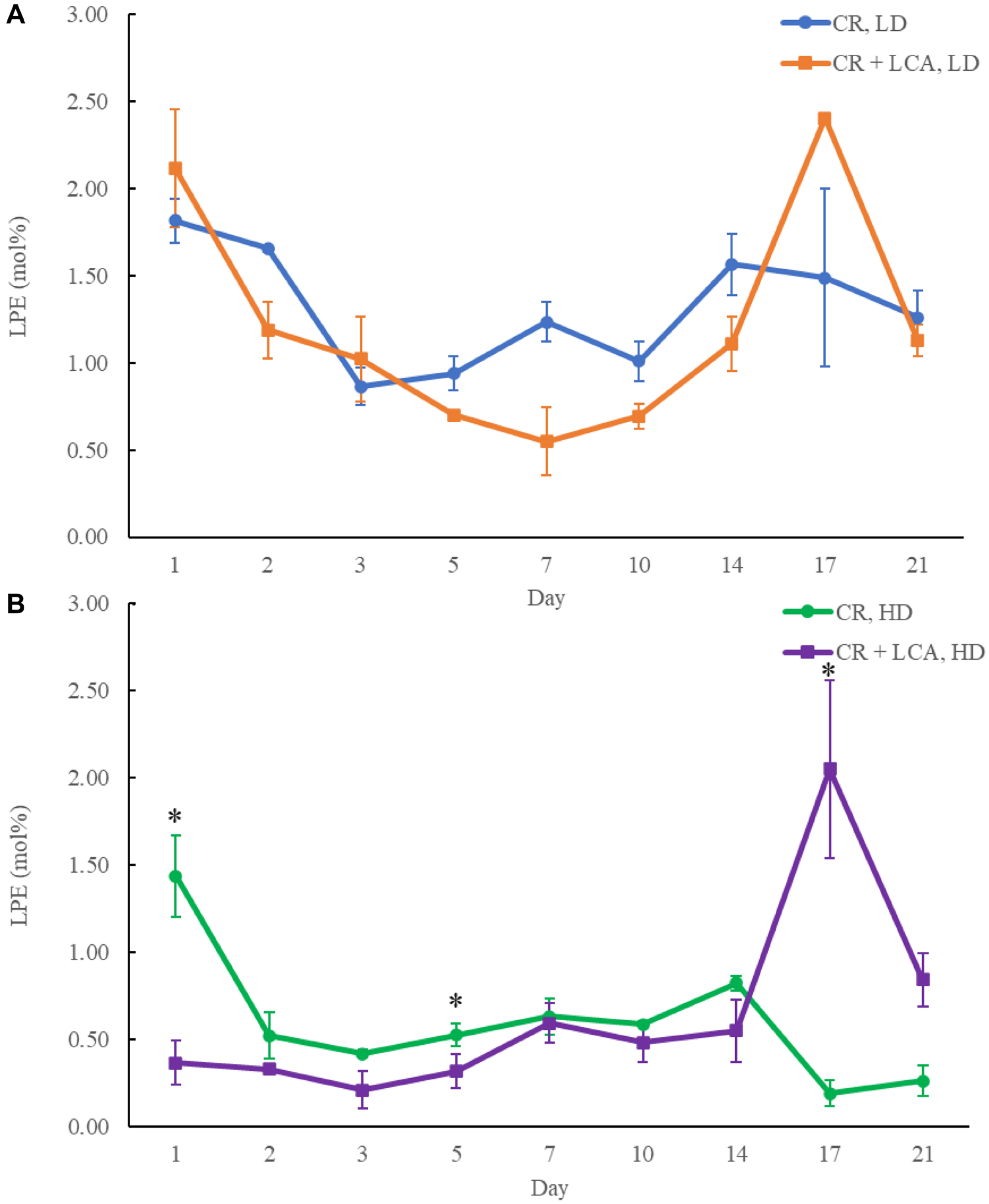

Supplementary Figure 35: LCA does not have a significant long-lasting effect on LPE concentration in HD and LD cells through the chronological lifespan. Samples of WT yeast cultured in YP medium initially containing 0.2% glucose (CR conditions) with 50 µM LCA or without it (control) were recovered on different days of culturing and subjected to centrifugation in Percoll density gradient to purify HD and LD cell sub-populations. LPE concentrations were measured by LC-MS/MS. LPE concentrations in LD (**A**) and HD (**B**) cells are shown. Data are presented as means ± SD ($n = 2$; $^{*}p < 0.05$).

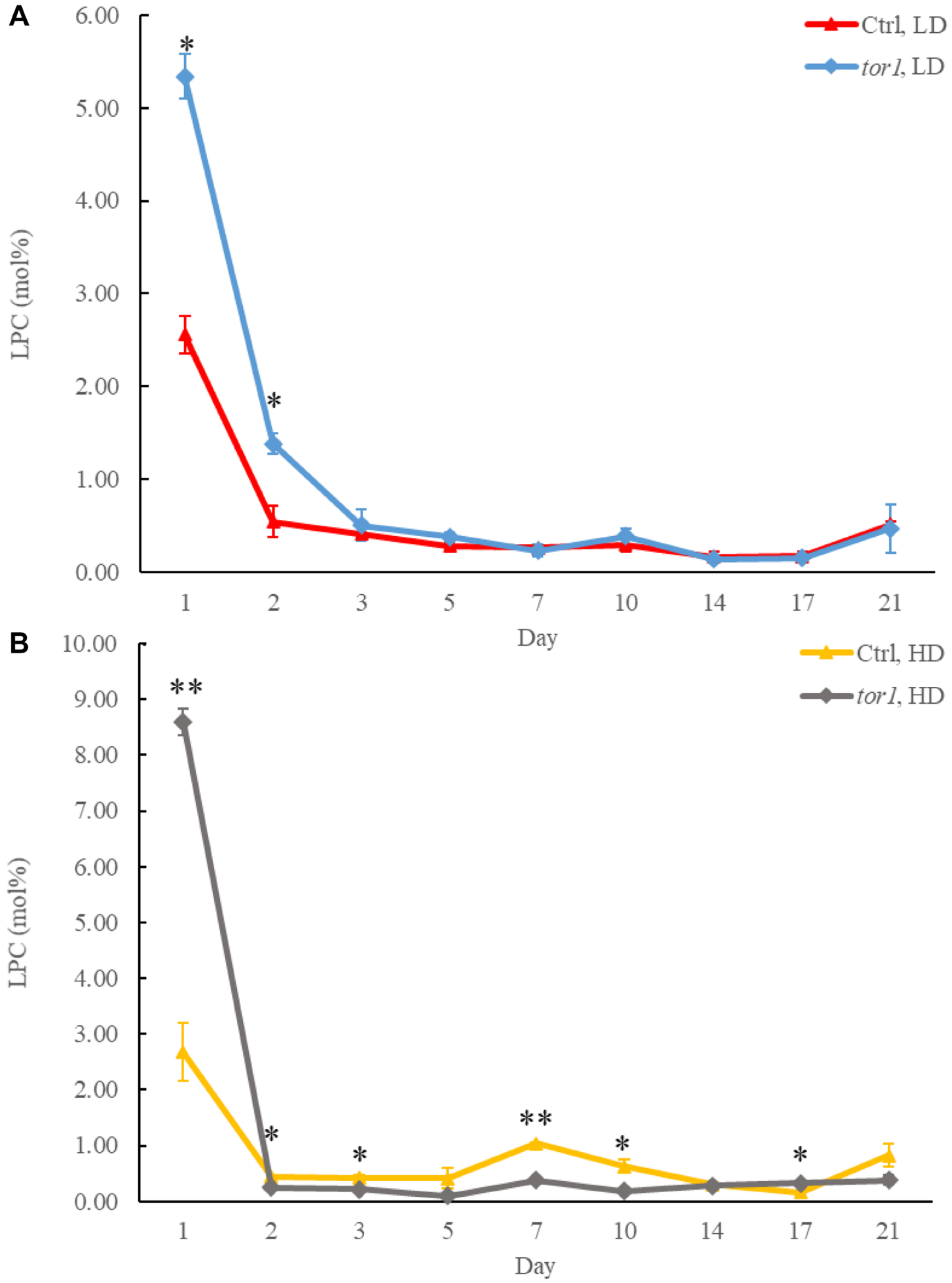

Supplementary Figure 36: The *tor1Δ* mutation does not have a significant long-lasting effect on LPC concentration in HD and LD cells through the chronological lifespan. Samples of WT (control) and *tor1Δ* yeast cultured in YP medium initially containing 2% glucose (non-CR conditions) were recovered on different days of culturing and subjected to centrifugation in Percoll density gradient to purify HD and LD cell populations. LPC concentrations were measured by LC-MS/MS. LPC concentrations in LD (**A**) and HD (**B**) cells are shown. Data are presented as means ± SD ($n = 2$; $^{*}p < 0.05$; $^{**}p < 0.01$). Abbreviation: Ctrl: control.

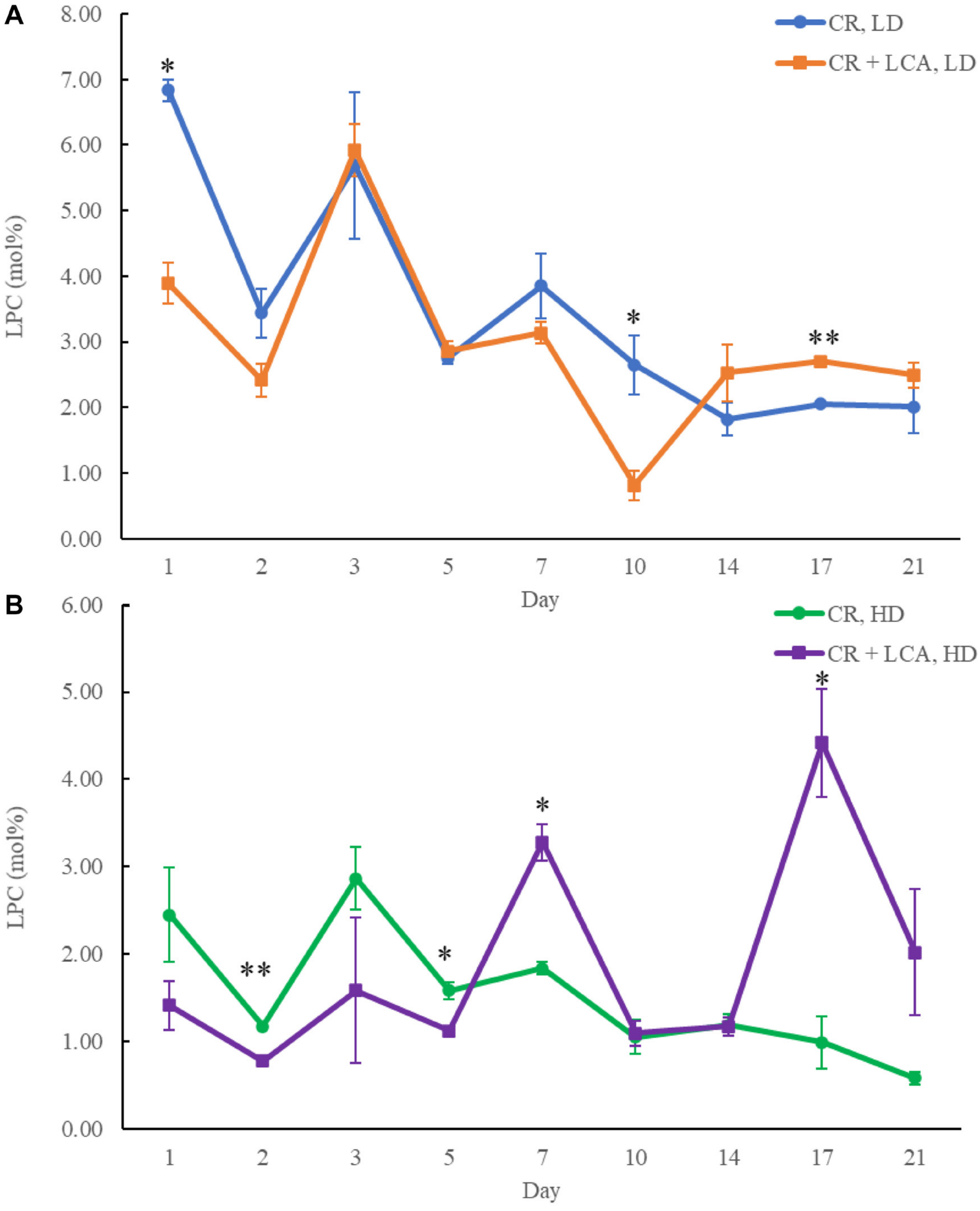

Supplementary Figure 37: LCA does not have a significant long-lasting effect on LPC concentration in HD and LD cells through the chronological lifespan. Samples of WT yeast cultured in YP medium initially containing 0.2% glucose (CR conditions) with 50 µM LCA or without it (control) were recovered on different days of culturing and subjected to centrifugation in Percoll density gradient to purify HD and LD cell populations. LPC concentrations were measured by LC-MS/MS. LPC concentrations in LD (**A**) and HD (**B**) cells are shown. Data are presented as means ± SD (n = 2; $^{*}p < 0.05$; $^{**}p < 0.01$).

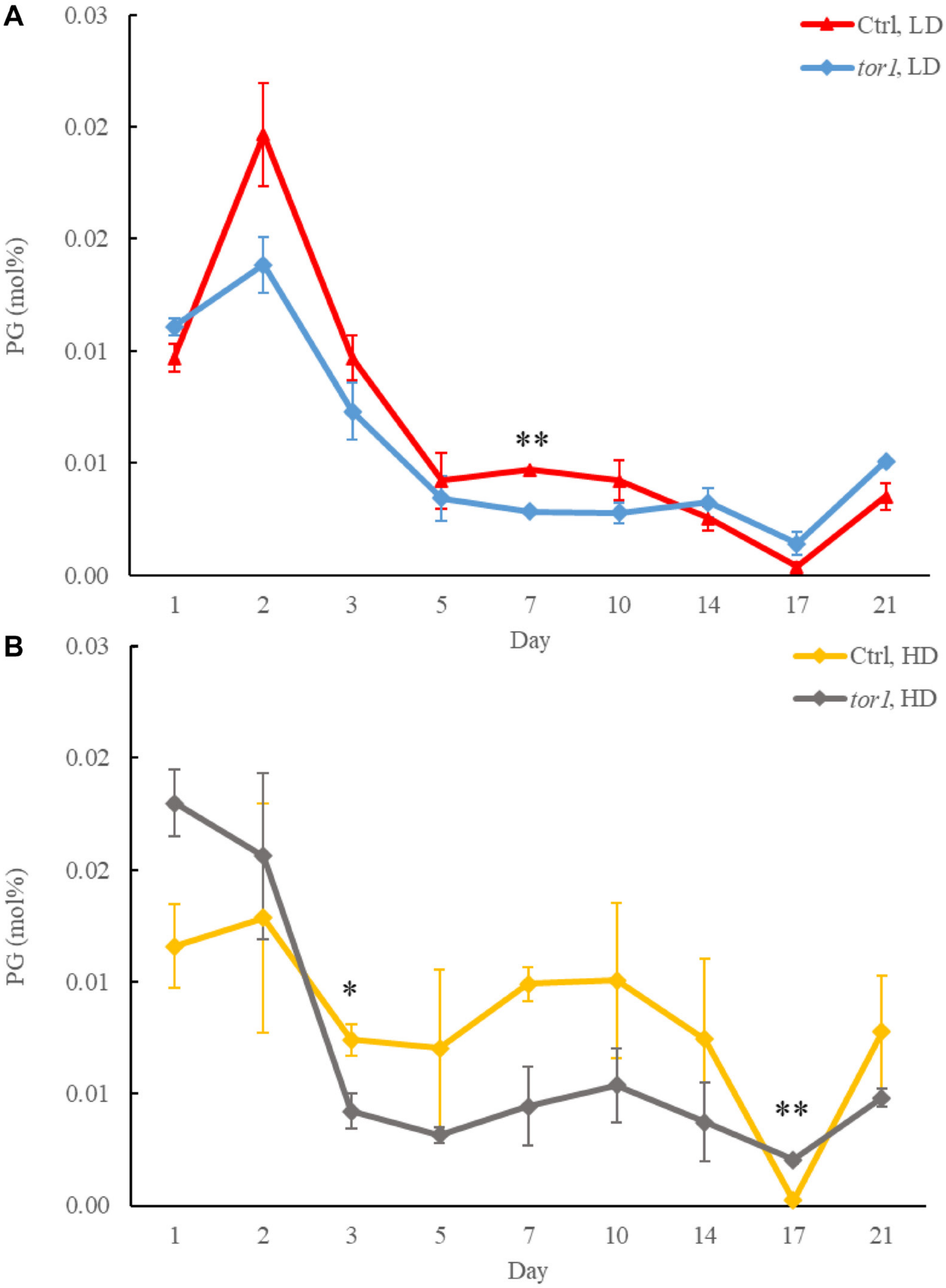

Supplementary Figure 38: The *tor1Δ* mutation does not have a significant long-lasting effect on PG concentration in HD and LD cells through the chronological lifespan. Samples of WT (control) and *tor1Δ* yeast cultured in YP medium initially containing 2% glucose (non-CR conditions) were recovered on different days of culturing and subjected to centrifugation in Percoll density gradient to purify HD and LD cell populations. PG concentrations were measured by LC-MS/MS. PG concentrations in LD (**A**) and HD (**B**) cells are shown. Data are presented as means ± SD ($n = 2$; $^{*}p < 0.05$; $^{**}p < 0.01$). Abbreviation: Ctrl: control.

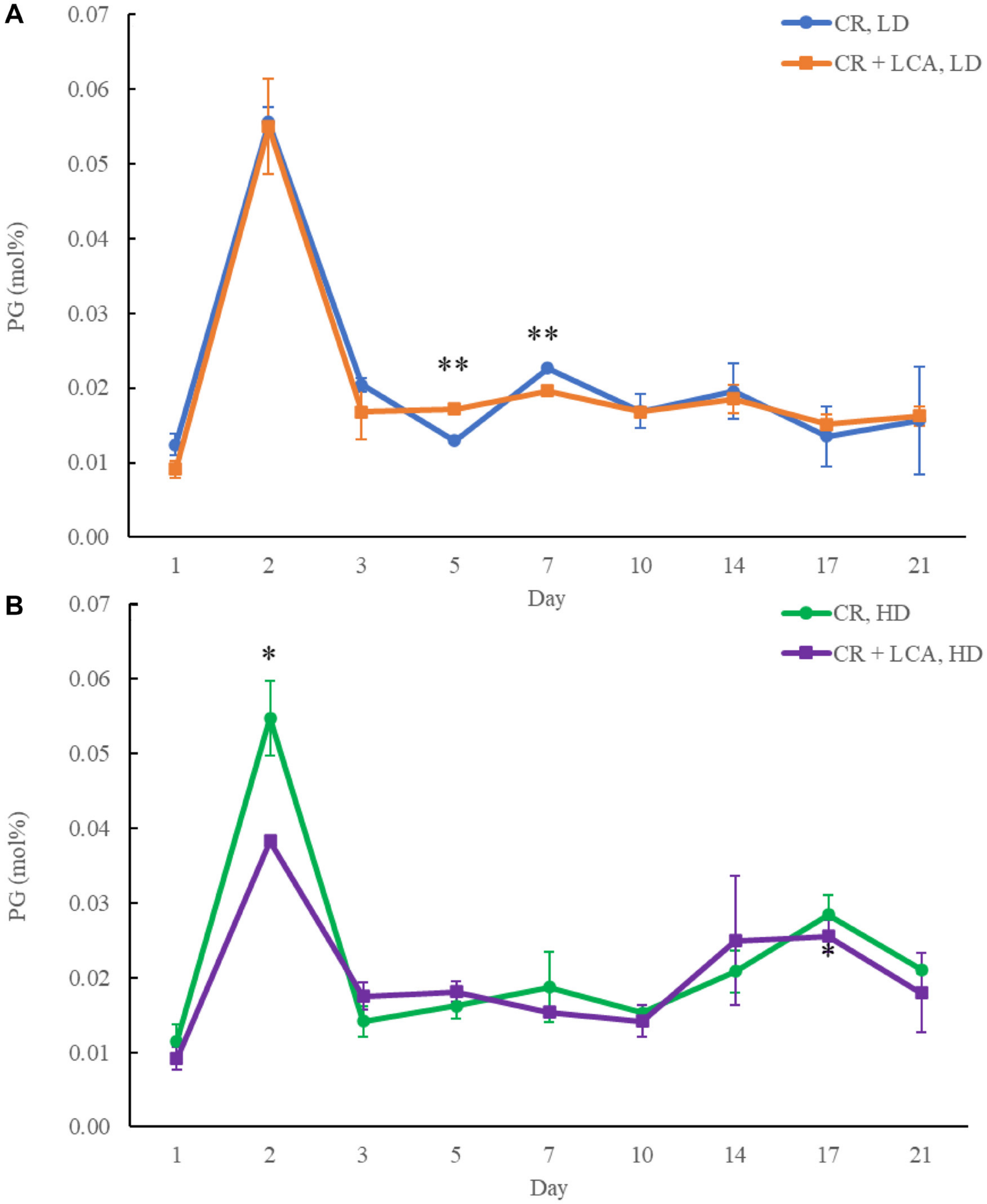

Supplementary Figure 39: LCA does not have a significant long-lasting effect on PG concentration in HD and LD cells through the chronological lifespan. Samples of WT yeast cultured in YP medium initially containing 0.2% glucose (CR conditions) with 50 µM LCA or without it (control) were recovered on different days of culturing and subjected to centrifugation in Percoll density gradient to purify HD and LD cell populations. PG concentrations were measured by LC-MS/MS. PG concentrations in LD (**A**) and HD (**B**) cells are shown. Data are presented as means ± SD ($n = 2$; $^{*}p < 0.05$; $^{**}p < 0.01$).

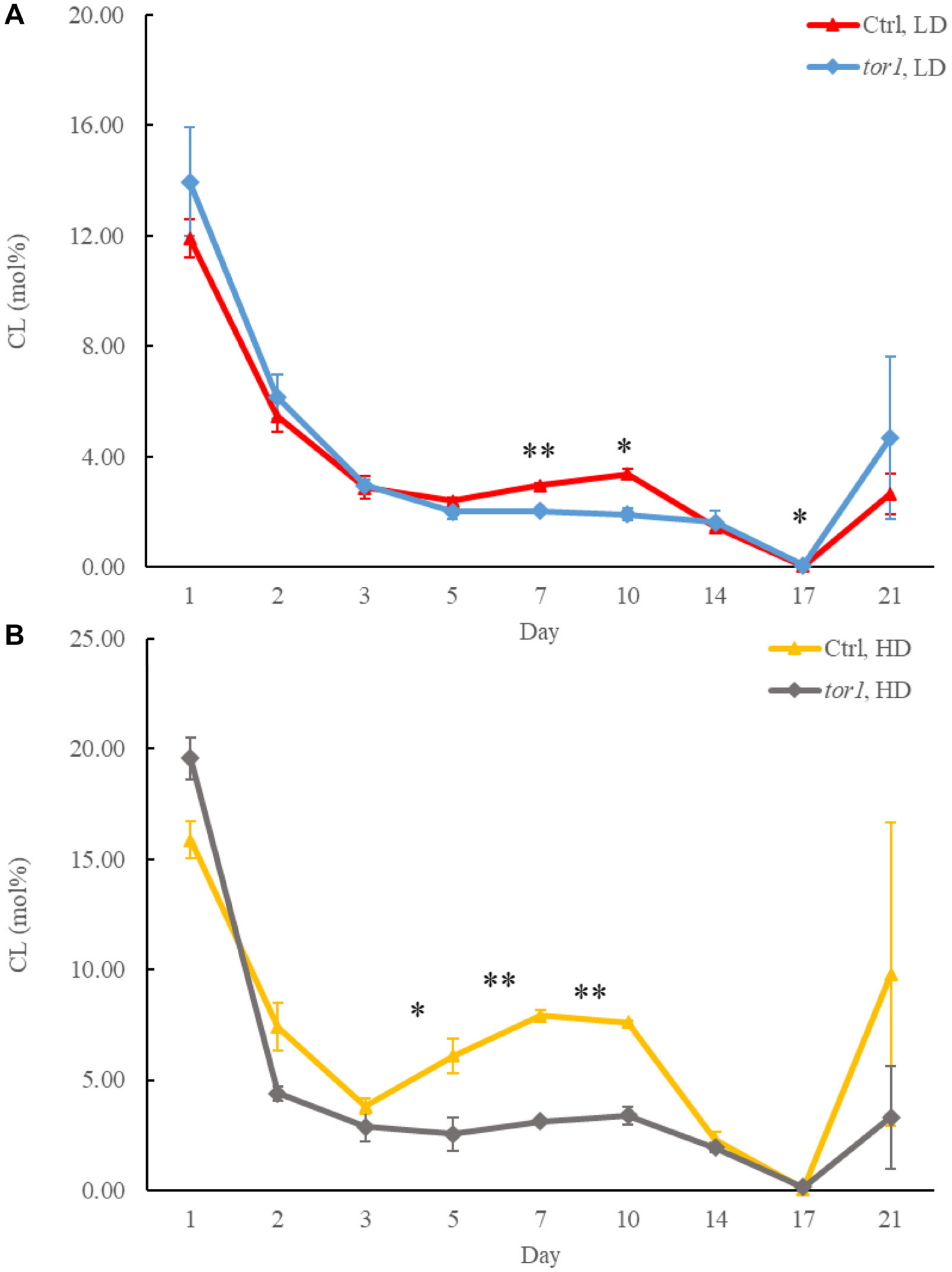

Supplementary Figure 40: The *tor1Δ* mutation does not have a significant long-lasting effect on CL concentration in HD and LD cells through the chronological lifespan. Samples of WT (control) and *tor1Δ* yeast cultured in YP medium initially containing 2% glucose (non-CR conditions) were recovered on different days of culturing and subjected to centrifugation in Percoll density gradient to purify HD and LD cell populations. CL concentrations were measured by LC-MS/MS. CL concentrations in LD (**A**) and HD (**B**) cells are shown. Data are presented as means ± SD ($n = 2$; $^{*}p < 0.05$; $^{**}p < 0.01$). Abbreviation: Ctrl: control.

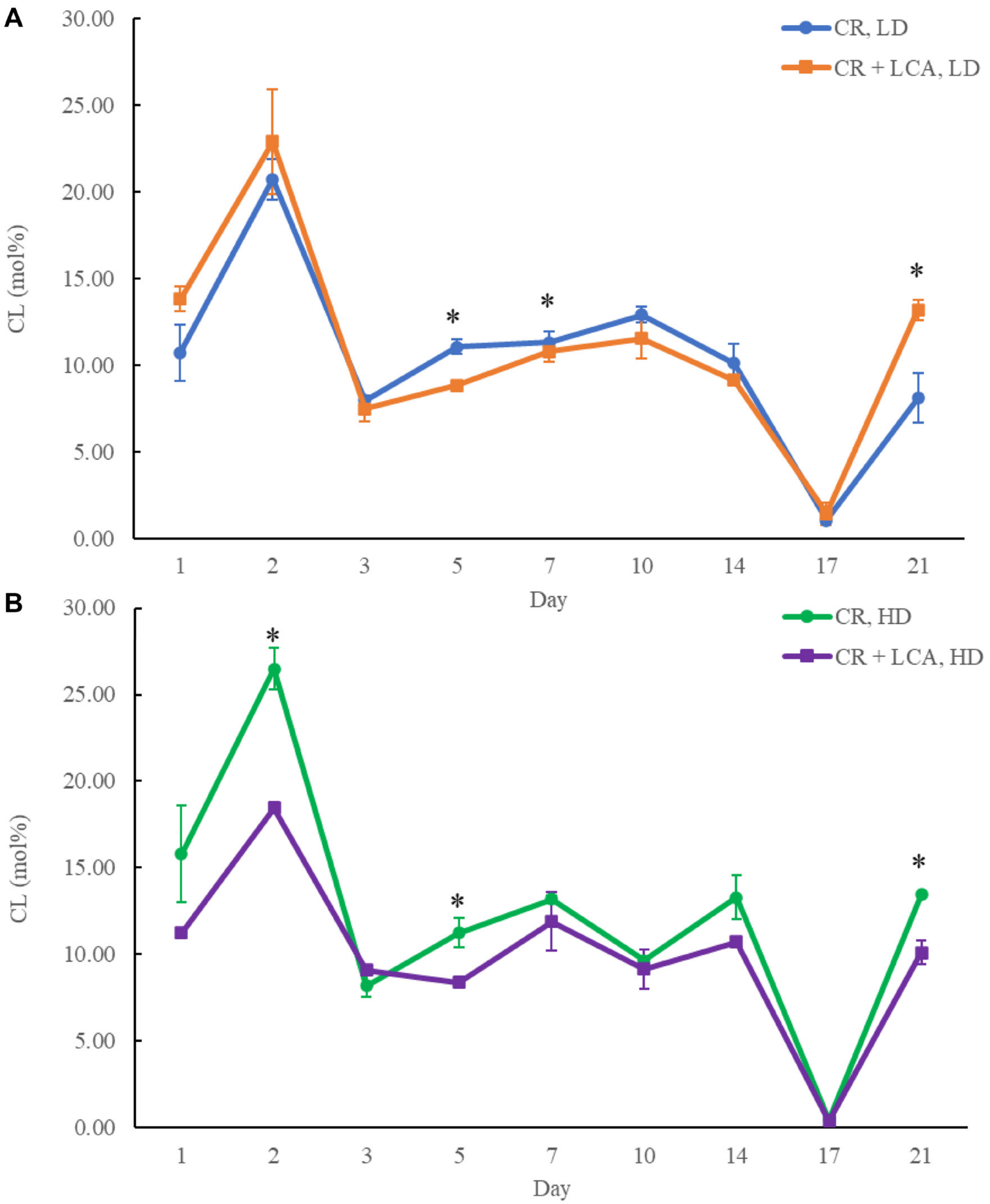

Supplementary Figure 41: LCA does not have a significant long-lasting effect on CL concentration in HD and LD cells through the chronological lifespan. Samples of WT yeast cultured in YP medium initially containing 0.2% glucose (CR conditions) with 50 µM LCA or without it (control) were recovered on different days of culturing and subjected to centrifugation in Percoll density gradient to purify HD and LD cell populations. CL concentrations were measured by LC-MS/MS. CL concentrations in LD (**A**) and HD (**B**) cells are shown. Data are presented as means ± SD ($n = 2$; $^{*}p < 0.05$).

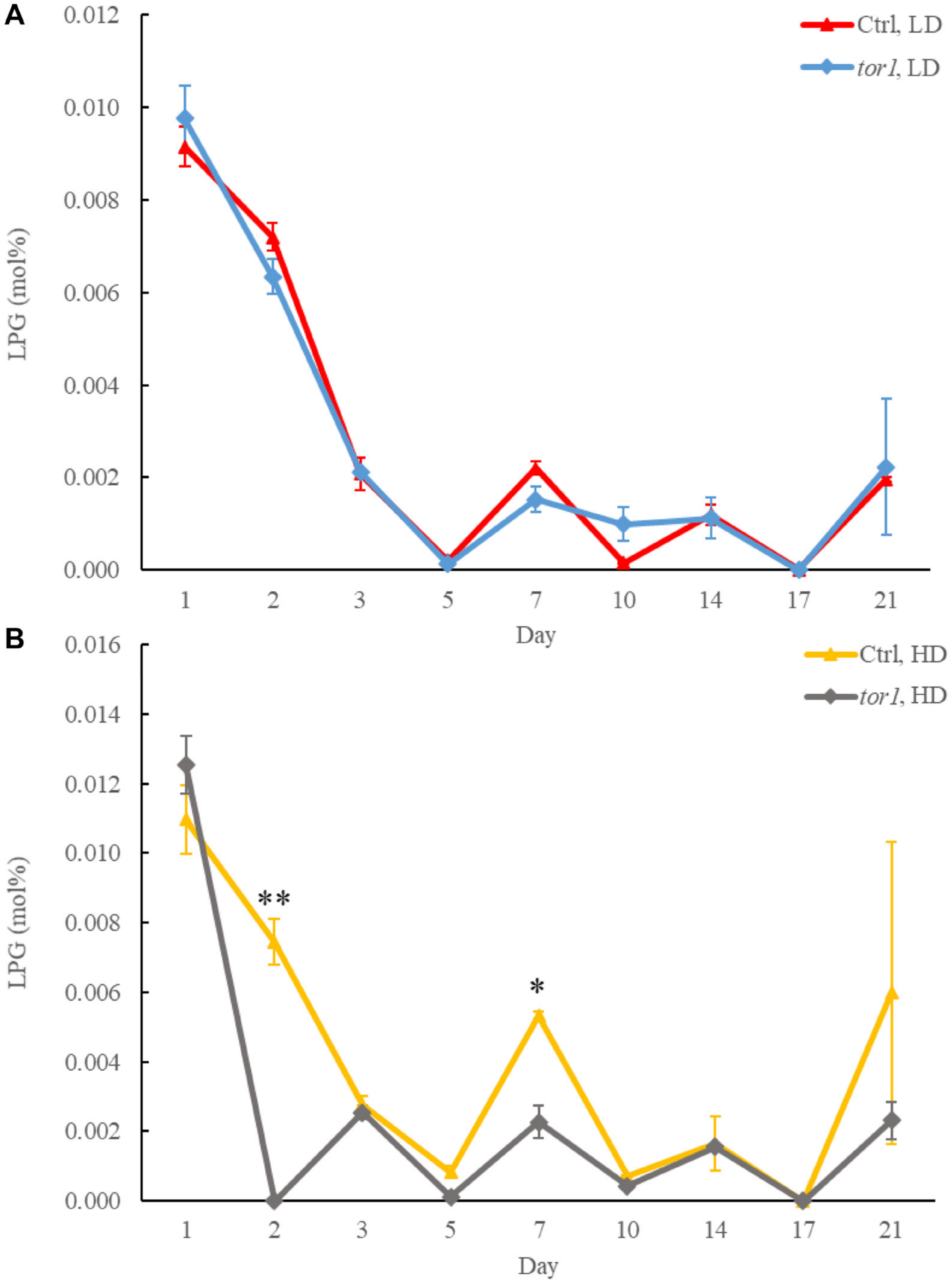

Supplementary Figure 42: The *tor1Δ* mutation does not have a significant long-lasting effect on LPG concentration in HD and LD cells through the chronological lifespan. Samples of WT (control) and *tor1Δ* yeast cultured in YP medium initially containing 2% glucose (non-CR conditions) were recovered on different days of culturing and subjected to centrifugation in Percoll density gradient to purify HD and LD cell populations. LPG concentrations were measured by LC-MS/MS. LPG concentrations in LD (**A**) and HD (**B**) cells are shown. Data are presented as means ± SD ($n = 2$; $^{*}p < 0.05$; $^{**}p < 0.01$). Abbreviation: Ctrl: control.

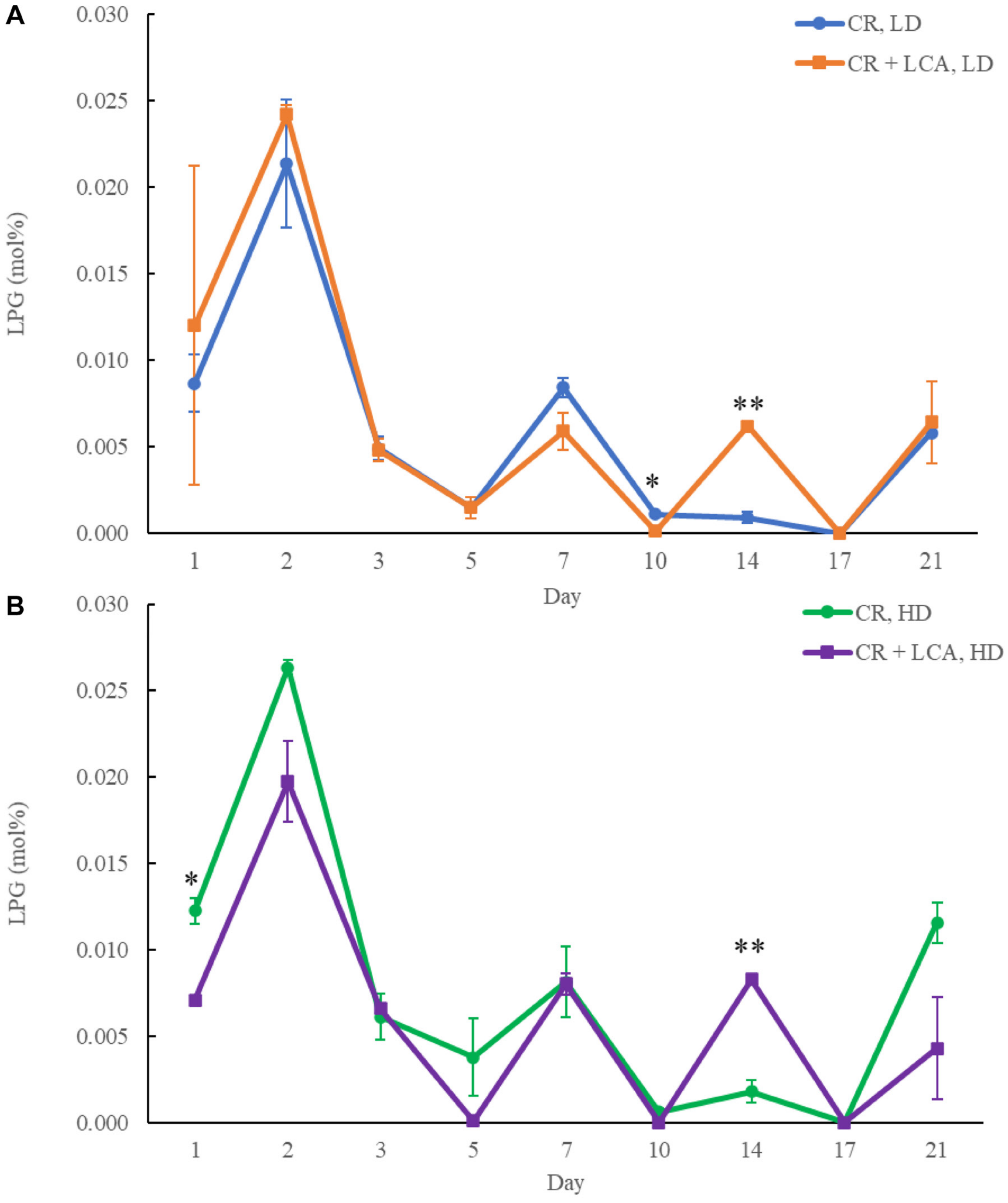

Supplementary Figure 43: LCA does not have a significant long-lasting effect on LPG concentration in HD and LD cells through the chronological lifespan. Samples of WT yeast cultured in YP medium initially containing 0.2% glucose (CR conditions) with 50 μM LCA or without it (control) were recovered on different days of culturing and subjected to centrifugation in Percoll density gradient to purify HD and LD cell populations. LPG concentrations were measured by LC-MS/MS. LPG concentrations in LD (**A**) and HD (**B**) cells are shown. Data are presented as means ± SD ($n = 2$; $^{*}p < 0.05$; $^{**}p < 0.01$).

Supplementary Table 1: The settings used for mass spectrometric analysis of lipids that were separated by LC

**FTMS - p resolution**	60000
**Mass range (dalton)**	150–2000
**Ion source type**	HESI
**Capillary temperature (°C)**	300
**Source heater temperature (°C)**	300
**Sheath gas flow**	10
**Aux gas flow**	5
**Positive polarity voltage (kV)**	3
**Negative polarity voltage (kV)**	3
**Source current (μA)**	100

Abbreviation: HESI: heated electrospray ionization.

Supplementary Table 2: The settings for detecting MS2 ions with the help of the Fourier transform analyzer

**Instrument polarity**	**Positive**	**Negative**
Activation type	High-energy-induced-collision-dissociation	Collision-induced-dissociation
Minimal signal required	5000	5000
Isolation width	2	2
Normalized collision energy	55	35
Default charge state	2	2
Activation time	0.1	10
FTMS - C resolution	7500	
5 most intense peaks were selected for ms/ms		

Supplementary Table 3: The parameters used to identify and quantify lipids from the LC-MS raw files containing full-scan MS1 data and data-dependent MS2 data

**Identification**	
Database	Orbitrap
Peak detection	Recall isotope (ON)
Search option	Product search Orbitrap
Search type	Product
Experiment type	LC-MS
Precursor tolerance	10 ppm
Product tolerance	High-energy-induced-collision-dissociation [ESI (+) mode]: 20 ppm<br>Collision-induced-dissociation [ESI (−) mode]: 0.5 Daltons

**Quantitation**	
Execute quantitation	ON
m/z tolerance	−5.0; +5.0
Tolerance type	ppm

**Filter**	
Top rank filter	ON
Main node filter	Main isomer peak
m-score threshold	5
c-score threshold	2
FFA priority	ON
ID quality filter	A: Lipid class & all fatty acids are completely identified<br>B: Lipid class & some fatty acids are identified<br>C: Lipid class or FA are identified<br>D: Lipid identified by other fragment ions (H2O, etc.)

**Lipid Class**	
High-energy-induced-collision-dissociation [ESI (+) mode]	PC, TAG
Collision-induced-dissociation [ESI (−) mode]	CER, CL, FFA, PE, PG, PI, PS

**Ions**	
High-energy-induced-collision-dissociation [ESI (+) mode]	+ H; + NH4; + Na
Collision-induced-dissociation [ESI (−) mode]	− H; − 2H; − HCOO
